# Memory of growth promotes early Ras GTPase activation to establish dominance of inherited cell ends in *Schizosaccharomyces pombe*

**DOI:** 10.1242/jcs.264600

**Published:** 2026-10-12

**Authors:** Walker H. Vickers, Maitreyi E. Das

**Affiliations:** Department of Biology, Boston College, 140 Commonwealth Ave, Chestnut Hill, MA 02467, USA

**Keywords:** Ras GTPase, Memory of growth, Cdc42, Polarity, Rax1, Rax2

## Abstract

Many eukaryotic cells maintain their polarity pattern and shape across multiple rounds of division. This requires that the daughter cells re-establish polarity at the correct site after cell division. Fission yeast cells (*Schizosaccharomyces pombe*) are bipolar; however, after division, growth initiates first at the old end of the cell that was inherited from the mother. This old-end dominance is dependent on that end having grown in the previous generation, suggesting that cells must recognize when an end has a history of growth. We find that this memory of growth is imparted by the growth-dependent localization of Rax1 (encoded by *SPAC23G3.05c*) and Rax2 proteins. Rax1 and Rax2 promote early activation of Ras1 GTPase at dividing cell ends by recruitment of its guanine nucleotide exchange factor Efc25. This enables timely transition of Cdc42 activity from the division site to the old end, thus giving this end an advantage in polarized growth. Consistently, modulating Ras1 activation via disruption of its regulators Efc25 and Gap1 leads to corresponding changes in old-end dominance. Here, we demonstrate the importance of memory of growth in maintaining persistent sites of growth from one generation to the next.

## Teaser

**Summary:** Memory of growth proteins Rax1 and Rax2 promote early activation of Ras1 at dividing cell ends to ensure timely re-initiation of growth at previously growing sites, maintaining cell polarity across division.

## INTRODUCTION

The importance of structure in determining function is a fundamental principle throughout biology, including in proteins, cells and multicellular organisms. The structure of a cell is established by polarized growth regulated by spatial cues that remodel the cytoskeleton to alter cell shape (Drubin and Nelson, 1996). In eukaryotes, polarity is primarily regulated through GTPases such as Cdc42, Rac and Rho (Etienne-Manneville, 2004; Johnson, 1999; Mosaddeghzadeh and Ahmadian, 2021; Nobes and Hall, 1999). These GTPases act as molecular switches that are activated by binding of GTP, promoted by guanine nucleotide exchange factors (GEFs) and deactivated when the hydrolysis of GTP to GDP is facilitated by GTPase-activating proteins (GAPs) (Bos et al., 2007). Polarization occurs through the localized activation of these GTPases to form the shape of the cell. Cells must not only establish polarization at the proper site, but also maintain it over time and across multiple generations. Polarity is often disrupted during cell division by processes such as cell rounding or redirection of polarity factors to the division site. This requires newly formed daughter cells to repolarize to initiate growth at the correct site and form their proper shape. Cells must therefore have mechanisms to recognize the appropriate site for growth re-initiation that can be maintained through division.

The fission yeast *Schizosaccharomyces pombe* is a well-established model for studying cell polarity (Chiou et al., 2017). Its bipolar growth and rod-like shape allow for quantification of changes in polarity as well as investigation of the regulation of competition between multiple sites of growth. Although *S. pombe* cells eventually become bipolar, they first grow only from the old end that is inherited from the mother cell. Growth from the new end formed by the division site is delayed until cells are approximately 9.5 μm in length, when bipolar growth begins by a process known as new-end take-off (NETO) (Mitchison and Nurse, 1985). Polarized growth is initiated by activation of Cdc42 by its GEFs, Gef1 and Scd1 (Chang et al., 1994; Coll et al., 2003; Hercyk et al., 2019; Kelly and Nurse, 2011). The two ends compete for Cdc42 activity as evidenced by its anti-correlated oscillatory dynamics (Das et al., 2012; Khalili et al., 2020; Xu and Jilkine, 2018). The old end initially outcompetes the new end and continues to have an advantage, maintaining a higher growth rate and increased Cdc42 activity even during bipolar growth (Das et al., 2012). This bias toward initiating growth at the old end is despite Cdc42 being primarily active at the division site (the future new end) and deactivated at the old ends during division (Tatebe et al., 2008; Wei et al., 2016). This suggests a mechanism of old-end dominance that promotes activation of Cdc42 and initiation of growth at that end. One factor important for old-end dominance is a previous history of growth. A cell end with a history of growth in one generation will grow in the next, even in most cell polarity mutants (Huisman and Brunner, 2011). Conversely, in many monopolar mutants, the cells that inherit an end that failed to grow in the previous generation lose old-end dominance. In some cases, daughter cells that inherit a non-growing end are precociously bipolar, such as in *gef1*Δ (Hercyk et al., 2019). In another category of monopolar mutants such as *tea1*Δ, the old end in daughter cells with no history of growth fails and, instead, growth occurs only at the new end (Niccoli et al., 2003). The dominance of the old end must therefore involve a memory of growth that allows the cell to recognize when an end has grown in a previous generation.

Polarity markers that act as a memory across generations have been identified in the regulation of bud-site selection in *Saccharomyces cerevisiae*. The proteins Rax1 and Rax2 (collectively referred to as Rax1/2) localize to the mother-bud neck and then remain at the former division site, marking bud scars through multiple cell cycles (Chen et al., 2000; Fujita et al., 2004). Rax1/2 then recruit the GAP Rga1 to these bud scars, inhibiting Cdc42 activity and preventing re-budding at the same site (Meitinger et al., 2014; Miller et al., 2017). However, in diploid cells, they promote a bipolar budding pattern through interaction with the polarity landmarks Bud8 and Bud9 (collectively Bud8/9), leading to activation of the Ras-like GTPase Rsr1 (Bi and Park, 2012; Kang et al., 2004; Krappmann et al., 2007). Rsr1 then promotes activation of Cdc42 through its GEF Cdc24 to establish the polarity site (Kang et al., 2010; Lee et al., 2015). Homologs of Rax1/2 in fission yeast have been identified and shown to play a role in regulation of polarity (Choi et al., 2006; Johnson, 2019); however, their specific function and the mechanism of their regulation in fission yeast are not well understood.

We find that the proteins Rax1/2 act as a memory of growth in fission yeast, localizing to cell ends in a growth-dependent manner and acting as a polarity landmark in newly divided daughter cells. When the Rax1/2 complex was disrupted, old-end dominance decreased and competition for Cdc42 activation between the two ends shifted to become more equal. The Rax1/2 complex promotes old-end dominance by recruitment of the Ras1 GTPase GEF Efc25 to dividing cell ends, increasing localized Ras1 activation. Ras1 has been shown to act as a functional homolog to Rsr1, promoting activation of Cdc42 through its GEF Scd1 (Lamas et al., 2020). Furthermore, we find that although the decrease in Cdc42 and Ras1 activity at the old end due to disruption of the Rax1/2 complex was only present during a short time window at cell separation, this delay was sufficient to decrease the advantage of the old end and allow for early new-end growth. Our work demonstrates the role of Rax1/2 and Ras1 in promoting initiation of growth at sites inherited from the previous cell cycle to maintain cell shape from one generation to the next.

## RESULTS

### Rax1 regulates the timing and rate of new-end growth

Previous reports on Rax1/2 in fission yeast found increased bipolarity and premature NETO in both *rax2*Δ and *rax1*Δ cells (Choi et al., 2006; Johnson, 2019). These findings are consistent with what we would expect of a memory-of-growth factor, as its loss should decrease old-end dominance. To determine the role of Rax1/2 in memory of growth, we deleted *rax1* (*SPAC23G3.05c*) and investigated cell polarity. Consistent with the previously described work, *rax1*Δ increased the percentage of cells with bipolar growth as shown by Calcofluor staining (Fig. 1A,B). We then measured the timing of NETO and the growth rate of each end using timelapse imaging. Cells lacking *rax1* had no apparent growth or cell cycle defect (Fig. S1) but did undergo premature NETO (Fig. 1C,D). Although the total growth rate of the cell did not change, growth at the old end decreased in *rax1*Δ, whereas growth rate of the new end increased, demonstrating a loss of old-end dominance (Fig. 1E–G). However, the dominance of the old end was not fully abolished by deletion of *rax1*, as the old end continued to initiate growth first and grew at a higher rate than the new end (Fig. 1G). Instead, loss of *rax1* decreased the advantage of the old end, allowing the new end to better compete for growth.

**Fig. 1.**
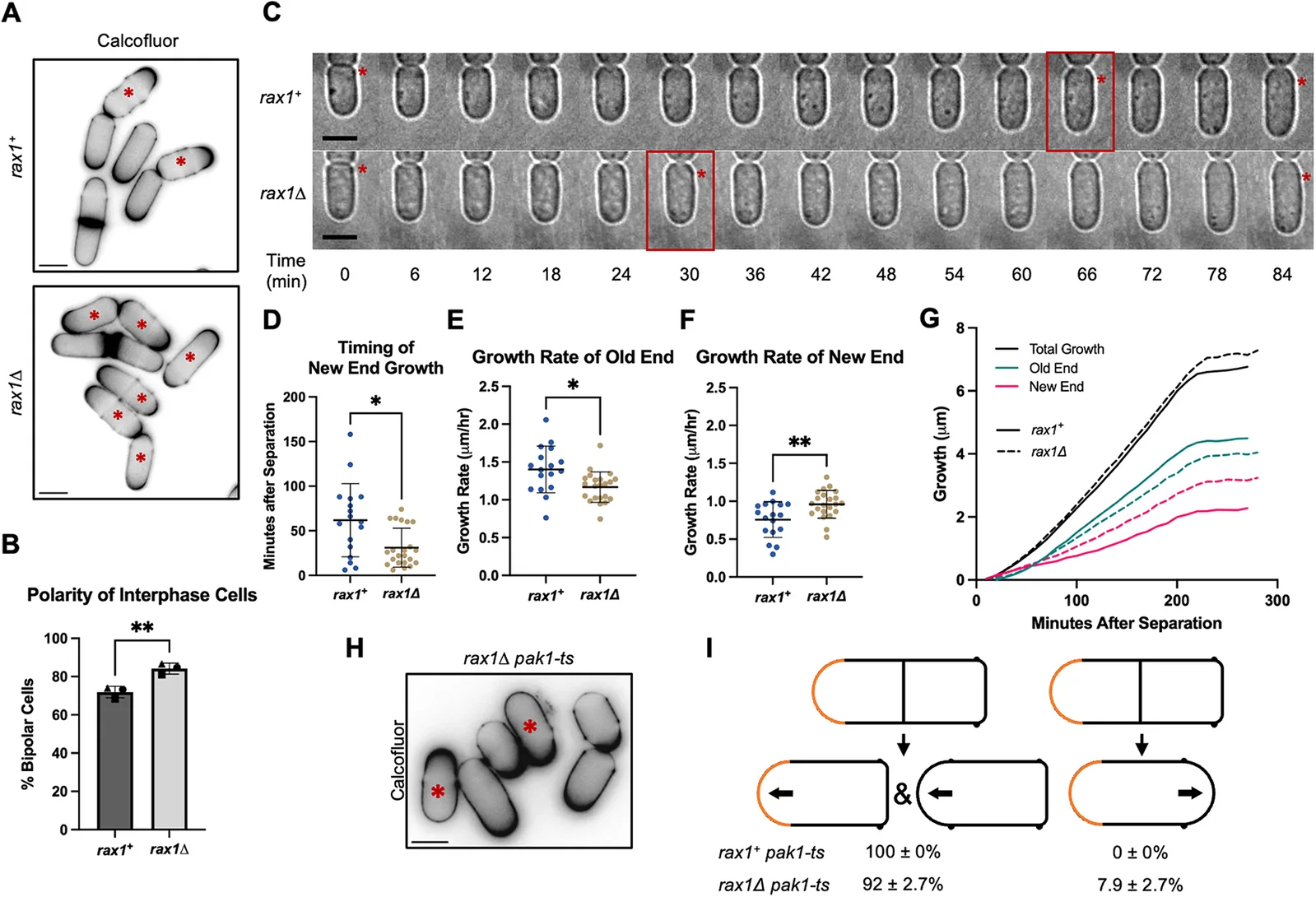
**Rax1 promotes old-end dominance and delays new-end growth.** (A) Polarized growth of *rax1^+^* and *rax1*Δ cells stained with Calcofluor White. Asterisks mark bipolar cells. (B) Percentage of interphase cells displaying bipolar growth as determined by Calcofluor staining. Each point corresponds to an independent experiment (*n*≥183 cells, *N*=3). (C) Montage of brightfield images of *rax1^+^* and *rax1*Δ cells. Time ‘0’ marks the onset of cell separation, red boxes mark onset of new-end growth, and asterisks mark the birth scar of the new end. (D) Quantification of the timing of onset of new-end growth in *rax1^+^* and *rax1*Δ cells. Each point represents a cell from one of three independent experiments (*n*≥17). (E,F) Average growth rate after onset of new-end growth at the old end (E) and new end (F) of *rax1^+^* and *rax1*Δ cells. Each point represents a cell imaged in one of three independent experiments (*n*≥17). (G) Mean increase in old-end, new end and total cell length over the cell cycle in *rax1^+^* and *rax1*Δ cells (*n*≥17). (H) Calcofluor staining of *rax1*Δ *pak1-ts* cells. Asterisks mark cells with monopolar growth at the new end that inherited an old end with a history of growth, which were identified as cells that lacked a birth scar near the non-growing end. (I) Growth patterns of cells with and without *rax1* in *pak1-ts* mutants. Orange marks previously growing ends, arrows mark new growth. Percentages were determined by Calcofluor staining and are mean±s.d. from three independent experiments (*n*≥158 cells, *N*=3). Scale bars: 5 μm. Error bars are mean±s.d. Significance was determined using an unpaired, two-tailed *t*-test with Welch's correction. *P<0.05; **P<0.01.

This decreased old-end dominance was also apparent when *rax1*Δ was combined with deletions of other polarity regulators, including mutants with increased monopolarity such as *gef1*Δ and *scd2*Δ. In most cases, deletion of *rax1* increased the percentage of bipolar cells relative to that in the mutant background (Table S1). Although loss of *rax1* failed to rescue bipolar growth in the strictly monopolar *orb2-34* (*pak1-ts*) mutant, we did observe a change in growth pattern. In the *pak1-ts* mutant, the daughter cell that inherits the previously growing end grows only from the old end, whereas the daughter that inherits a non-growing end initiates growth at the new end (Kim et al., 2003). However, when observing Calcofluor-stained *rax1*Δ *pak1-ts* cells, we identified a small percentage of cells that appeared to have inherited an end with a history of growth yet failed to re-establish growth at that site. Instead, the daughter cells grew from the former division site as if they inherited a non-growing end. (Fig. 1H,I). This supports the hypothesis that Rax1 acts as the memory of growth and deletion of *rax1* decreases the ability of the cell to recognize previously growing ends.

### Rax1/2 localize to cell ends in a growth-dependent manner

The function of a memory-of-growth marker requires that its localization to the ends be dependent on growth. It must then remain stable at those sites of growth through division. Both Rax1 and Rax2 have been reported to localize to cell ends, including in dividing cells (Choi et al., 2006; Johnson, 2019). We visualized Rax1 and Rax2 by tagging them with an N-terminal mNeonGreen (mNG) or a C-terminal GFP, respectively. Both appeared as puncta along the cell membrane clustered at cell ends in both growing and dividing cells (Fig. 2A). mNG–Rax1 and Rax2–GFP also localized in a non-constricting ring at the division site (Fig. S2A). Consistent with their *S. cerevisiae* homologs and previous reports (Johnson, 2019; Kang et al., 2004), localization of Rax2 to the cell ends was dependent on Rax1, with Rax2–GFP failing to localize to the cell ends in a *rax1*Δ background (Fig. S2B). We also found that Rax1 and Rax2 interact with one another to form a complex, as mNG–Rax1 co-immunoprecipitated with Rax2–GFP (Fig. 2B).

**Fig. 2.**
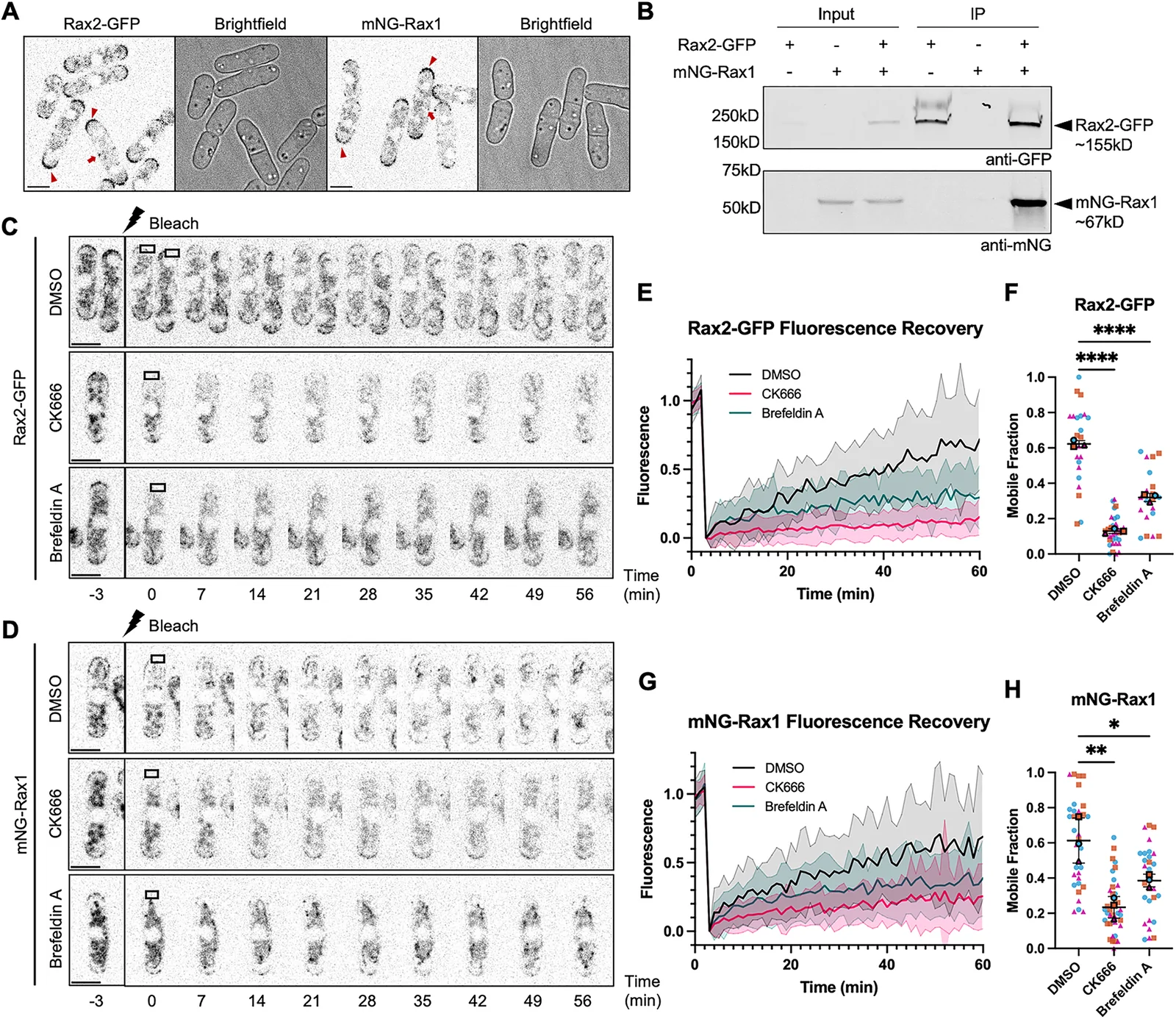
**Localization of Rax1/2 to cell ends is growth dependent.** (A) Localization of Rax2–GFP and mNG–Rax1. Arrowheads show localization to the old ends of dividing cells and newly formed daughter cells, whereas arrows mark division site localization. (B) Co-immunoprecipitation (IP) of mNG–Rax1 with Rax2–GFP. Membrane fractions from cells expressing Rax2–GFP, mNG–Rax1 or both were immunoprecipitated with anti-GFP beads. Western blotting was then performed with anti-GFP and anti-mNG antibodies. *N*=1 independent experiment. (C,D) Montage showing fluorescence recovery after photobleaching of Rax2–GFP (C) or mNG–Rax1 (D) in cells treated with DMSO (1%), CK666 (100 μM) or brefeldin A (100 μg/ml). Time ‘0’ is the first timepoint after photobleaching. Boxes show photobleached regions. (E) Mean and s.d. of normalized fluorescence recovery of Rax2–GFP over time (*n*≥20 cells collected over three independent experiments). (F) Quantification of the mobile fraction of fluorescence recovery of Rax2–GFP after 60 min. Outlined points represent the mean from one independent experiment with small points corresponding to individual cells (*n*≥6 cells, *N*=3). (G) Mean and s.d. of normalized fluorescence recovery of mNG–Rax1 over time (*n*≥20 cells collected over three independent experiments). (H) Quantification of the mobile fraction of fluorescence recovery of mNG–Rax1 after 60 min. Outlined points represent the mean from one independent experiment with small points corresponding to individual cells (*n*≥7 cells, *N*=3). Scale bars: 5 μm. Error bars are mean±s.d. Significance was determined by one-way ANOVA followed by Dunnett's multiple comparisons test. *P<0.05; **P<0.01; ****P<0.0001.

To confirm that localization of the Rax1/2 complex is growth dependent, we performed fluorescence recovery after photobleaching (FRAP) at cell ends. Growth was inhibited by treatment with CK666, an Arp2/3 inhibitor (Nolen et al., 2009), or brefeldin A, an ER-to-Golgi transport inhibitor (Turi et al., 1994), with DMSO as a control. Both mNG–Rax1 and Rax2–GFP displayed recovery at the ends of DMSO-treated interphase cells, consistent with a slow accumulation during growth (Fig. 2C,D). When growth was arrested with CK666 or brefeldin A, fluorescence recovery was lost, with a significant decrease in the mobile fraction of Rax2–GFP in both conditions (Fig. 2E,F). mNG–Rax1 showed a similar trend, though with a smaller effect, likely due to increased bleaching during image acquisition (Fig. 2G,H). We also examined fluorescence recovery of Rax1/2 at dividing cell ends in control cells, as growth arrests during division (Ray et al., 2010). Rax2–GFP and mNG–Rax1 both showed decreased recovery in dividing cells, further supporting the growth-dependent localization of Rax1/2 (Fig. S2C,D). A previous report indicated that Rax2–GFP is lost from cell ends when the actin or microtubule cytoskeleton is disrupted (Choi et al., 2006). However, we found that localization of mNG–Rax1 and Rax2–GFP was unchanged even when the cytoskeleton was disrupted, as they remained at cell ends after treatment with either latrunculin B or methyl-2-benzimidazole carbamate (MBC) (Fig. S3A). To further investigate the role of actin in Rax1/2 localization, we also performed FRAP of Rax2–GFP in *sty1*Δ cells, which continue to grow even after actin depolymerization (Mutavchiev et al., 2016). We found that Rax2–GFP showed recovery after photobleaching at *sty1*Δ cell ends even after CK666 treatment, although recovery was lower compared to that in the DMSO-treated control, likely due to a decreased growth rate in these cells (Fig. S3B–D).

### Rax1 promotes the timely transition of Cdc42 activity from the division site to the old ends of separating cells

Although our data show that Rax1 and Rax2 act as memory-of-growth markers, we wanted to identify the mechanism by which they promote old-end dominance. Therefore, we examined the localization of the major regulator of polarized growth, Cdc42. The dominance of the old end is evident in the competition for active Cdc42, with the old end maintaining higher levels of Cdc42 activation until late in the cell cycle (Das et al., 2012). When visualizing Cdc42–GTP using the fluorescent probe CRIB–3×GFP (Tatebe et al., 2008), a higher fraction of the total end intensity of CRIB–3×GFP was present at the old end than at the new end. However, *rax1*Δ increased the proportion of Cdc42 activity present at the new end, leading to a more equal distribution between the two ends (Fig. 3A,B; Fig. S4A). We also observed this shift in distribution towards the new end in the localization of the Cdc42 GEF Scd1 and its scaffold Scd2 (Fig. 3A,C,D; Fig. S4B). Interestingly, although the growth rate at the old end of *rax1*Δ cells decreased along with the increase in new-end growth, there was no significant change in the level of Cdc42–GTP or its activators at the old end. Instead, the change in distribution between the two ends was primarily due to an increase at the new end (Fig. S4C–H). Growth rate is therefore not directly determined by the amount of Cdc42–GTP but must be regulated by another limiting factor.

**Fig. 3.**
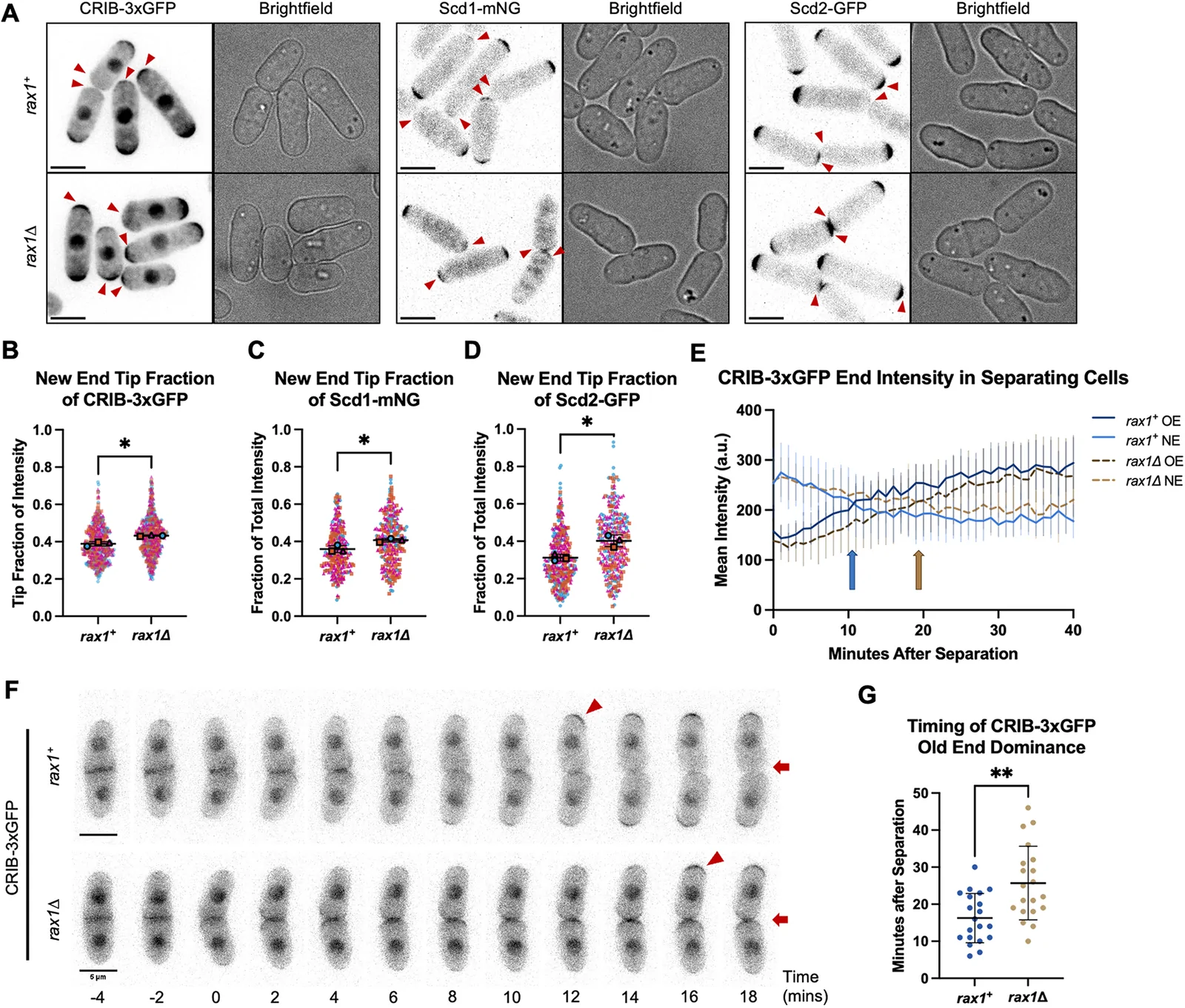
**Loss of *rax1* decreases old-end dominance by delaying Cdc42 activation at that end during cell separation.** (A) Localization of CRIB–3×GFP, Scd1–mNG and Scd2–GFP at the ends of *rax1^+^* and *rax1*Δ cells. Images are sum projections of *z*-series. Arrowheads mark new ends, which were identified by their proximity to the birth scar. (B–D) Quantification of the fraction of total tip intensity of CRIB–3×GFP (B), Scd1–mNG (C) and Scd2–GFP (D) at the new ends of *rax1^+^* and *rax1*Δ interphase cells. Outlined points represent the mean from one independent experiment, with small points corresponding to individual cells (*n*≥54 cells, *N*=3). (E) Quantification of mean and s.d. of CRIB–3×GFP intensity over time at the old ends (OE) and new ends (NE) of separating *rax1^+^* and *rax1*Δ cells (*n*=20 cells imaged over three independent experiments). Arrows mark the point where old-end intensity becomes higher than new-end intensity. (F) Montage of CRIB–3×GFP in separating *rax1^+^* and *rax1*Δ cells. Arrowheads mark localization of CRIB–3×GFP at old ends, whereas arrows mark the new ends. Time ‘0’ is the onset of cell separation. (G) Quantification of the timepoint at which old-end CRIB–3×GFP intensity becomes higher than new-end intensity. Data points correspond to individual cells (*n*=20 cells collected over three independent experiments). Scale bars: 5 μm. Error bars are mean±s.d. a.u., arbitrary units. Significance was determined using an unpaired, two-tailed *t*-test with Welch's correction. *P<0.05; **P<0.01.

To further understand how *rax1*Δ cells decrease in old-end dominance and initiate early new-end growth, we also measured the dynamics of Cdc42 activity in separating daughter cells by timelapse imaging of CRIB–3×GFP. In the wildtype, Cdc42 was active at the division site until just before cell separation, when Cdc42 activity shifted to the old end and left the recently formed new end. However, CRIB–3×GFP remained longer at the new end of *rax1*Δ cells, whereas accumulation was delayed at the old end (Fig. 3E,F). The timepoint at which the old end began to outcompete the new end, determined by the time when CRIB–3×GFP intensity at the old end became higher than that at the new end, was delayed by ∼9 min in *rax1*Δ (Fig. 3E, gold arrow) compared to that in *rax1^+^* (Fig. 3E, blue arrow). We found that in *rax1^+^* cells, CRIB–3×GFP intensity at the old end overcame the intensity at the new end 16 min after cell separation, whereas in *rax1Δ* cells, this occurred after 26 min (Fig. 3G). This delay in old-end localization was also present for Scd1–mNG and Scd2–GFP (Fig. S5). Loss of *rax1* does not abolish competition for active Cdc42, as the anti-correlation and period of CRIB–3×GFP oscillations remained similar to that of *rax1^+^* cells (Fig. S6A,B). Instead, it equalizes the competition between the two ends, leading to more symmetric oscillations (Fig. S6C). These results show that Rax1 promotes timely re-initiation of Cdc42 activation at the cell ends following division.

### Ras1 GTPase activity at the ends of dividing cells is dependent on Rax1

Next, we investigated how the Rax1/2 complex regulates Cdc42 activity. In *S. cerevisiae*, polarized activation of Cdc42 downstream of Rax1/2 is through the activity of Rsr1 (Kang et al., 2010; Bi and Park, 2012). The Rax1/2 complex promotes localization of the polarity landmarks Bud8/9, which recruit the GEF of Rsr1, Bud5 (Kang et al., 2004; Krappmann et al., 2007). Active Rsr1 then interacts with the GEF Cdc24, which activates Cdc42 (Chiou et al., 2017). Although homologs of Bud8/9 have not been identified in fission yeast, the Ras GTPase in fission yeast, Ras1, has been found to promote activation of Cdc42 through interaction with Scd1, acting in a functionally similar pathway to that of Rsr1 (Chang et al., 1994; Lamas et al., 2020). We therefore hypothesized that Rax1/2 regulate Cdc42 through Ras1. We visualized Ras1 activity in cells that expressed the fluorescent probe RasAct^GFP^, which binds to Ras1–GTP (Merlini et al., 2018). In contrast to Cdc42, Ras1 was highly active at the ends of dividing cells as well as the division site (Fig. 4A,D). Without *rax1*, RasAct^GFP^ no longer localized to the ends of dividing cells, instead increasing localization to the division site (Fig. 4A–E). In timelapse imaging of dividing cells, Ras1 activity increased at the cell ends, until it peaked right before cell separation (Fig. 4D,E, red arrowhead). However, in *rax1Δ* cells, activation of Ras1 instead peaked at the division site while failing to accumulate at the old ends (Fig. 4D,E, blue arrowhead). Interestingly, this change in Ras1 activity was limited to division, as RasAct^GFP^ intensity at the ends of interphase cells was similar in both *rax1*Δ and *rax1^+^* cells (Fig. 4F,G). If Ras1 activation at cell ends during division is dependent on Rax1, then there should be no active Ras1 at ends without a history of growth. We therefore examined RasAct^GFP^ localization in *tea1*Δ monopolar cells. Tea1 is delivered to the cell ends through microtubules and is required for the initiation of a second site of growth (Kim et al., 2003; Niccoli et al., 2003). Consistent with our hypothesis, Ras1 activity was limited to the previously growing end of dividing *tea1*Δ cells and was absent from the non-growing end (Fig. 4H).

**Fig. 4.**
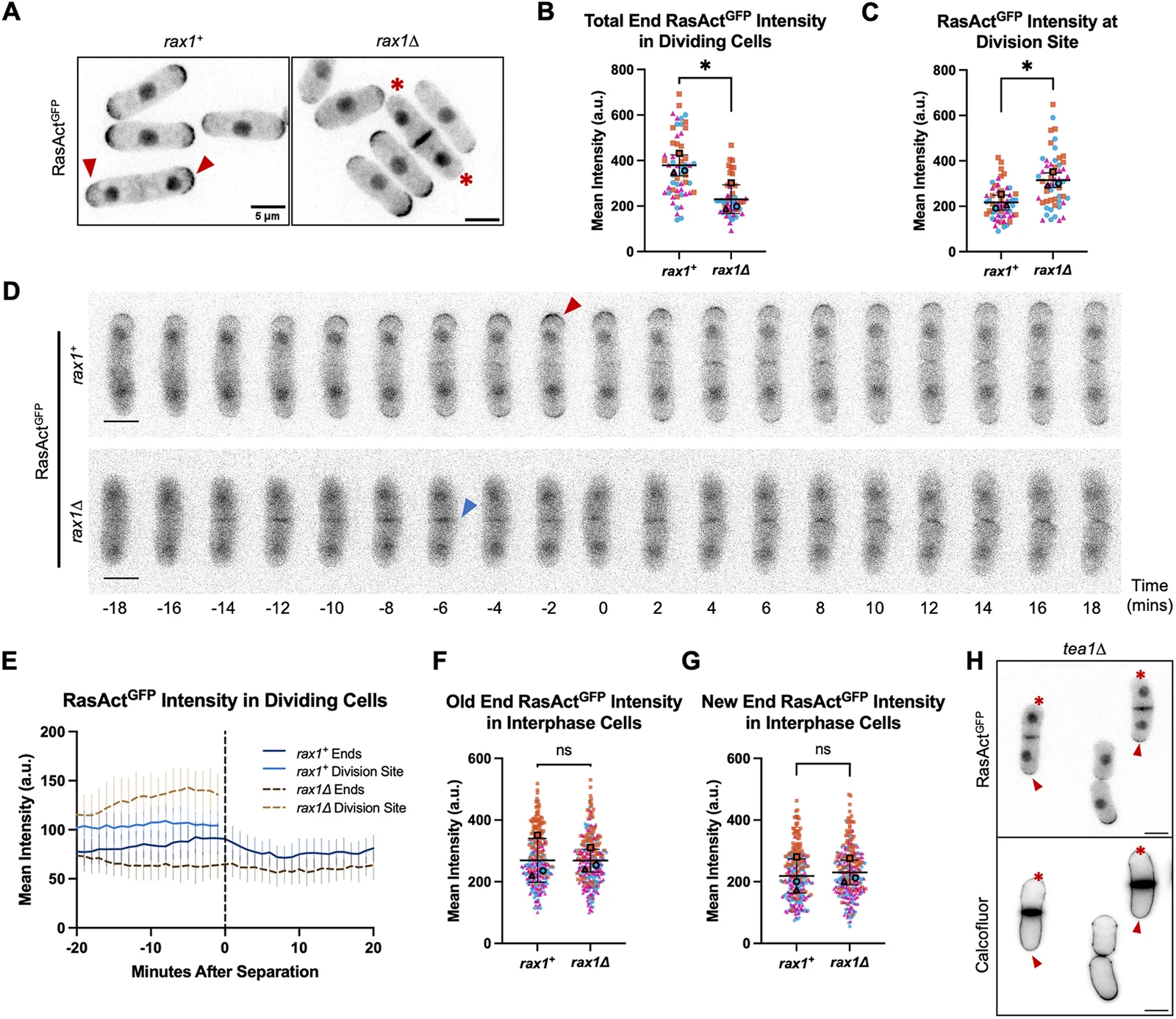
**Rax1 promotes activation of Ras1 at the ends of dividing cells.** (A) Localization of RasAct^GFP^ in *rax1^+^* and *rax1*Δ cells. Images are sum projections of *z*-series. Arrowheads mark Ras1 activity at the ends of dividing cells, whereas asterisks mark cell ends without Ras1 activity. (B,C) Quantification of total RasAct^GFP^ intensity at the ends (B) and division site (C) of dividing cells. Outlined points represent the mean from one independent experiment, with small points corresponding to individual cells (*n*≥15 cells, *N*=3). (D) Montage of RasAct^GFP^ in separating *rax1^+^* and *rax1*Δ cells. The red arrowhead marks the peak intensity of RasAct^GFP^ at the ends of *rax1^+^* cells, and the blue arrowhead marks the peak intensity at the division site in *rax1*Δ cells. Time ‘0’ is onset of cell separation. (E) Quantification of the mean and s.d. of RasAct^GFP^ intensity over time in *rax1^+^* and *rax1*Δ cells mid-separation (*n*=10 cells imaged over three independent experiments). (F,G) Quantification of mean RasAct^GFP^ intensity at the old ends (F) and new ends (G) of interphase *rax1^+^* and *rax1*Δ cells. Outlined points represent the mean from one independent experiment, with small points corresponding to individual cells (*n*≥78, *N*=3). (H) Localization of RasAct^GFP^ in *tea1*Δ cells stained with Calcofluor White. Arrowheads mark dividing cells where RasAct^GFP^ is localized to the end that shows evidence of growth in the previous generation, with asterisks marking non-growing ends lacking RasAct^GFP^ (*N*=1 independent experiment). The RasAct^GFP^ image is a sum projection of a *z*-series. Scale bars: 5 μm. Error bars are mean±s.d. a.u., arbitrary units. Significance was determined using an unpaired, two-tailed *t*-test with Welch's correction. ns, not significant; *P<0.05.

### Efc25 localization to cell ends during division is dependent on Rax1

Because Efc25 is the main GEF of Ras1 during mitotic growth and is required for the role of Ras1 in morphogenesis (Papadaki et al., 2002; Tratner et al., 1997), we hypothesized that the Rax1/2 complex regulates Ras1 activation through Efc25. Therefore, we examined localization of Efc25 with and without *rax1*. We first imaged Efc25 C-terminally tagged with GFP expressed under its native promoter. Although in dividing cells, Efc25–GFP appeared primarily at the division site, we also observed faint localization at the cell ends (Fig. 5A). When measuring Efc25–GFP intensity along the cell cortex, we found that Efc25–GFP intensity peaked at the tips of *rax1^+^* dividing cells, and that this peak was severely diminished in *rax1Δ* cells (Fig. 5B; Fig. S7A). We also observed a trend in increasing Efc25–GFP intensity at the division site of *rax1Δ* cells; however, the change was not statistically significant (Fig. 5C, *P*=0.0908). The low abundance of Efc25 and biological noise might have obscured the signal difference that we observed between these strains. To more clearly visualize Efc25 localization, we tagged it with an N-terminal mNG and expressed it under the *nmt41* thiamine-inducible promoter in cells lacking endogenous *efc25* (Basi et al., 1993). We then grew cells in minimal medium without thiamine and imaged mNG–Efc25. When overexpressed, mNG–Efc25 clearly localized to the ends of dividing cells as well as at the division site in *rax1^+^* cells (Fig. 5D). Without *rax1*, mNG–Efc25 no longer accumulated at cell ends during division and instead increased at the division site, consistent with the trends seen with endogenously tagged Efc25–GFP (Fig. 5D–F; Fig. S7B). Interestingly, total intensity of both Efc25–GFP and mNG–Efc25 was higher in *rax1*Δ cells than in *rax1^+^*, suggesting that Rax1 regulates not only Efc25 localization, but also its protein levels (Fig. 5G,H; Fig. S7). However, it appears that it is the change in distribution of Efc25 between the ends and the division site that is important for old-end dominance and not the amount of protein, as overexpression of mNG–Efc25 did not affect bipolarity in *rax1^+^* or *rax1*Δ cells (Fig. 5I).

**Fig. 5.**
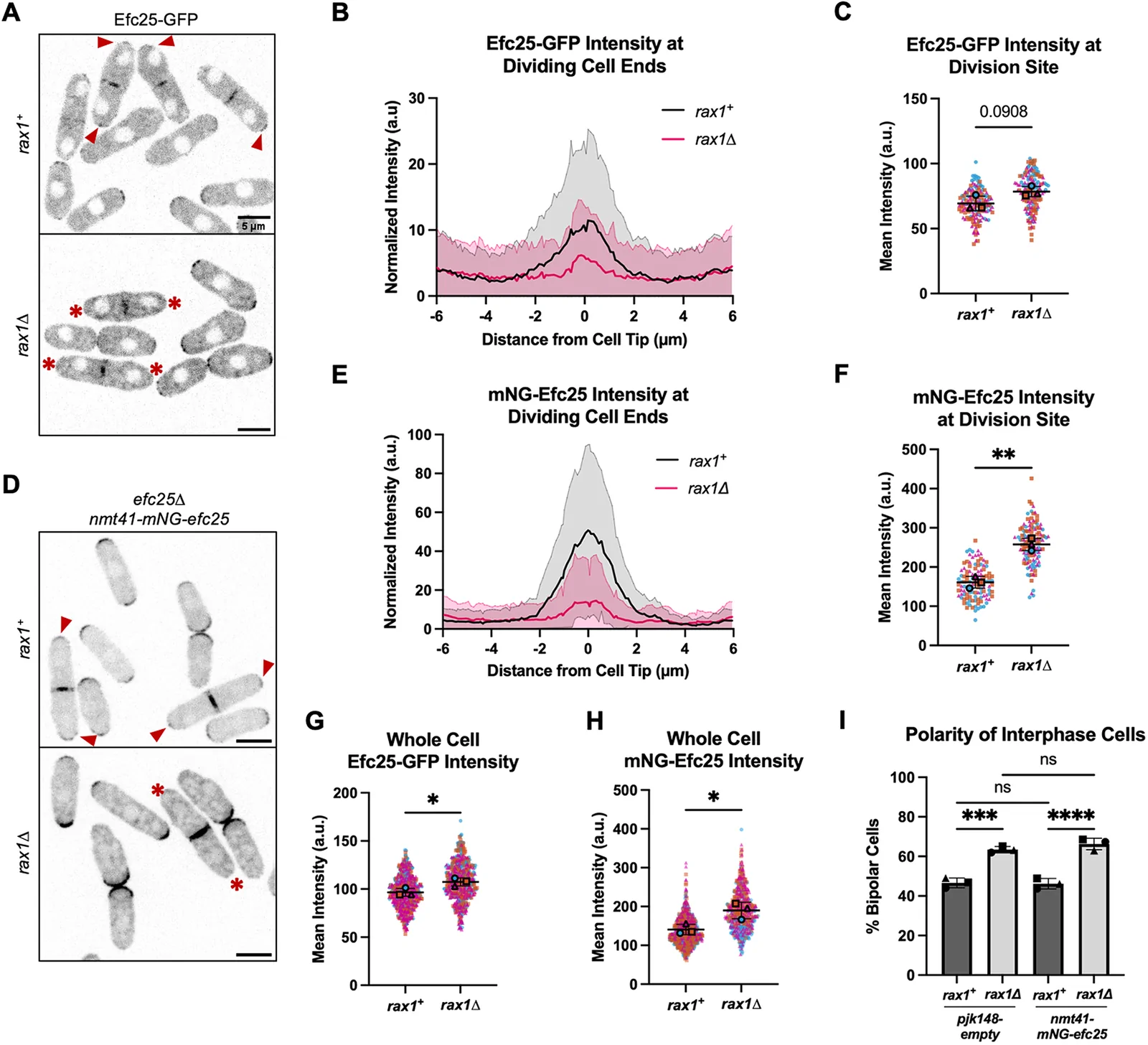
**Rax1 promotes localization of Efc25 to cell ends during division.** (A) Localization of Efc25–GFP in *rax1^+^* and *rax1*Δ cells. Images are sum projections of the middle four planes of a *z*-series. Arrowheads mark Efc25–GFP present at the ends of dividing cells, whereas asterisks mark cell ends without Efc25–GFP. (B) Plot profile of the mean and s.d. of normalized Efc25–GFP intensity along the cell cortex in dividing cells (*n*≥157 cells collected across three independent experiments). Intensity was normalized to that at the cell sides. (C) Quantification of Efc25–GFP intensity at the division site. Outlined points represent the mean from one independent experiment, with small points corresponding to individual cells. Significance was determined using an unpaired, two-tailed *t*-test with Welch's correction (*n*≥43, *N*=3). (D) Localization of mNG–Efc25 expressed from the *nmt41* promoter in *rax1^+^* and *rax1*Δ cells grown in Edinburgh minimal medium lacking thiamine. Images are sum projections of the middle four planes of a *z*-series. Arrowheads mark mNG–Efc25 present at the ends of dividing cells, whereas asterisks mark cell ends without mNG–Efc25. (E) Plot profile of the mean and s.d. of normalized mNG–Efc25 intensity along the cell cortex in dividing cells (*n*≥115 cells collected across three independent experiments). Intensity was normalized to that at the cell sides. (F) Quantification of mNG–Efc25 intensity at the division site. Outlined points represent the mean from one independent experiment, with small points corresponding to individual cells. Significance was determined using an unpaired, two-tailed *t*-test with Welch's correction (*n*≥33, *N*=3). (G,H) Quantification of whole-cell intensity of Efc25–GFP (G) or mNG–Efc25 (H) in *rax1^+^* and *rax1*Δ cells. Outlined points represent the mean from one independent experiment, with small points corresponding to individual cells. Significance was determined using an unpaired, two-tailed *t*-test with Welch's correction (*n*≥123, *N*=3). (I) Percentage of interphase cells displaying bipolar growth as determined by Calcofluor staining. Each point corresponds to the mean of an independent experiment (*n*≥308, *N*=3). Significance was determined by one-way ANOVA followed by Šídák's multiple comparisons test. Scale bars: 5 μm. Error bars are mean±s.d. a.u., arbitrary units. ns, not significant; *P<0.05; **P<0.01; ***P<0.001; ****P<0.0001.

We next investigated whether the recruitment of Efc25 by Rax1/2 is through direct interaction or by an indirect mechanism. To determine interaction between Rax1/2 and Efc25, we used Förster resonance energy transfer (FRET) measured by the increase in donor fluorescence after acceptor photobleaching. We first measured FRET efficiency between Rax1 and Rax2 to determine whether they directly bind each other to form a complex. Rax1 and Rax2 have been shown to interact with one another in other yeasts (Kang et al., 2004; Li et al., 2025), so we predicted that they would interact in fission yeast as well. mNG–Rax1 and Rax2–mScarlet did colocalize at the cell ends and had a FRET efficiency of 13.7%, indicating that they do directly interact to form a complex (Fig. S8A,B). Next, we measured the FRET efficiency between Efc25–mScarlet and either mNG–Rax1 or Rax2–GFP. Although donor fluorescence of mNG–Rax1 and Rax2–GFP increased after photobleaching of Efc25–mScarlet, the increase was mostly not significant, with a maximum FRET efficiency of 6.61% during interphase (Fig. S8C–E). The small amount of Efc25–mScarlet localized to cell ends during division as well as the low abundance of all three proteins might have impacted the ability to detect interaction through this method. To further investigate this potential interaction, we performed immunoprecipitation of Rax2–GFP from membrane fractions and found no interaction with mNG–Efc25 (Fig. S8F). Based on this negative result and the inconclusive FRET results, we cannot conclude that the Rax1/2 complex directly interacts with Efc25. Further research is needed to determine whether the low abundance and membrane localization of the proteins involved make this interaction difficult to detect or whether Rax1/2 promote Efc25 recruitment to dividing cell ends through an alternate mechanism.

### Active Ras1 promotes old-end dominance

Although our results show that Rax1 regulates the localization of Efc25 and Ras1 activity during division, our next step was to determine whether this loss of Efc25 from the ends of dividing cells is responsible for the loss of old-end dominance in *rax1*Δ cells*.* To address this, we investigated the polarity of cells lacking active Ras1. Deletion of the GEF Efc25 removes Ras1 activation during mitotic growth (Merlini et al., 2018). Thus, we crossed *efc25*Δ with *rax1*Δ and looked at the bipolarity of the single and double deletions with lectin and Calcofluor staining. Despite being much shorter and wider with clear shape defects, *efc25*Δ cells showed the same increase in bipolarity as *rax1*Δ cells (Fig. 6A,B). Furthermore, loss of *rax1* in an *efc25*Δ background did not increase bipolarity compared to that in *efc25*Δ alone (Fig. 6B). This epistatic interaction between *efc25*Δ and *rax1*Δ indicates that activation of Ras1 through Efc25 is downstream of Rax1/2. We would expect that if active Ras1 promotes old-end dominance, then cells lacking Ras1 activity should show a delay in old-end activation of Cdc42, similar to what we observed in *rax1*Δ. Timelapse imaging of CRIB–3×GFP in separating *efc25*Δ cells showed Cdc42 activity present for a longer duration at the former division site, whereas activation at the old end was significantly delayed (Fig. 6C,D). This decrease in old-end dominance was even more pronounced in *efc25*Δ than in *rax1*Δ, supporting our model that localization of Efc25 to promote Ras1 activation at cell ends during division is important for old-end dominance.

**Fig. 6.**
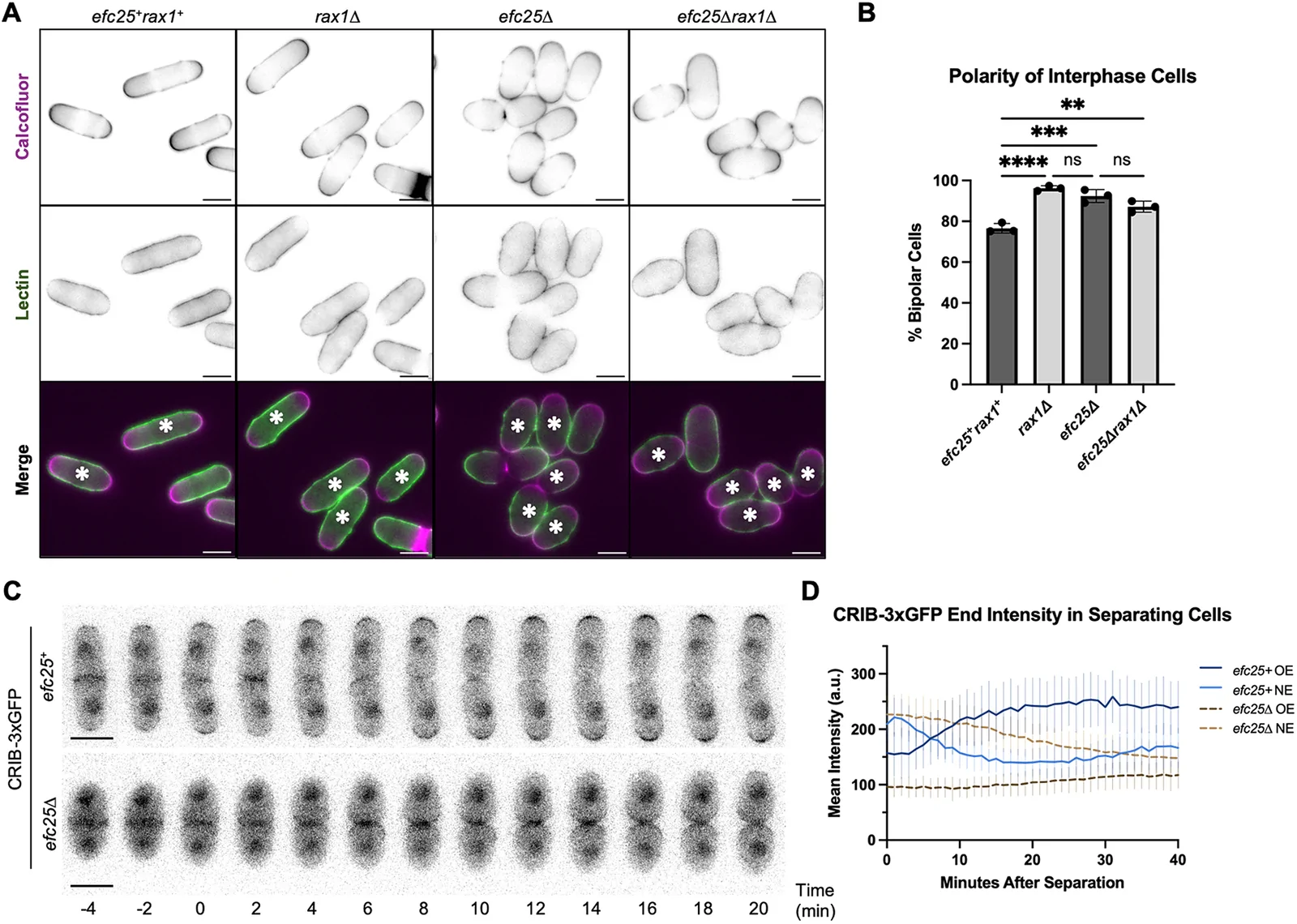
**Activation of Ras1 by its GEF Efc25 promotes Rax1-mediated old-end dominance.** (A) Polarized growth of cells stained with lectin–Alexa Fluor 488 and Calcofluor White in the indicated strains. Asterisks mark bipolar cells. (B) Percentage of interphase cells displaying bipolar growth as determined by Calcofluor and lectin staining. Each point corresponds to the mean of an independent experiment (*n*≥158, *N*=3). Significance was determined by one-way ANOVA followed by Tukey's multiple comparisons test. (C) Montage of CRIB–3×GFP in separating *efc25+* and *efc25*Δ cells. Time ‘0’ is onset of cell separation. (D) Quantification of mean and s.d. of CRIB–3×GFP intensity over time at the old ends (OE) and new ends (NE) of separating *efc25+* and *efc25*Δ cells (*n*≥46 cells imaged across three independent experiments). Scale bars: 5 μm. Error bars are mean±s.d. a.u., arbitrary units. ns, not significant; **P<0.01; ***P<0.001; ****P<0.0001.

If the loss of active Ras1 at the ends of dividing cells is responsible for the decreased old-end dominance we observed in *rax1*Δ cells, then increasing Ras1 activity should promote old-end dominance and rescue the increased bipolarity in *rax1*Δ. To investigate this, we examined the effect of loss of the GAP of Ras1, Gap1 (Imai et al., 1991). Deletion of *gap1* leads to hyperactivation of Ras1 across the cell cortex (Lamas et al., 2020; Merlini et al., 2018). We measured the proportion of bipolar cells in *gap1*Δ cells as well as in the double deletion of *gap1*Δ *rax1*Δ and found that loss of *gap1* decreased bipolarity. Furthermore, *gap1*Δ *rax1*Δ cells showed the same decrease in bipolarity as that in *gap1*Δ alone (Fig. 7A,B). This rescue of *rax1*Δ by loss of *gap1* demonstrates that hyperactivation of Ras1 bypasses the need for the Rax1/2 complex to promote old-end dominance. Timelapse imaging of CRIB–3×GFP in separating *gap1*Δ cells further supported these results, as Cdc42 was activated earlier at the old end when *gap1* was deleted (Fig. 7C,D). Interestingly, active Cdc42 remained active at the former division site longer in *gap1*Δ cells than in *gap1^+^* (Fig. 7C). Despite this increase in early new-end Cdc42 activity, *gap1*Δ cells were more monopolar than *gap1^+^* cells, suggesting that it is the timing of old-end activation of Cdc42 rather than the amount of activity at the new end that is important for old-end dominance. Our earlier finding that growth rate is not directly determined by the level of Cdc42 activity at that end is also supported by this observation.

**Fig. 7.**
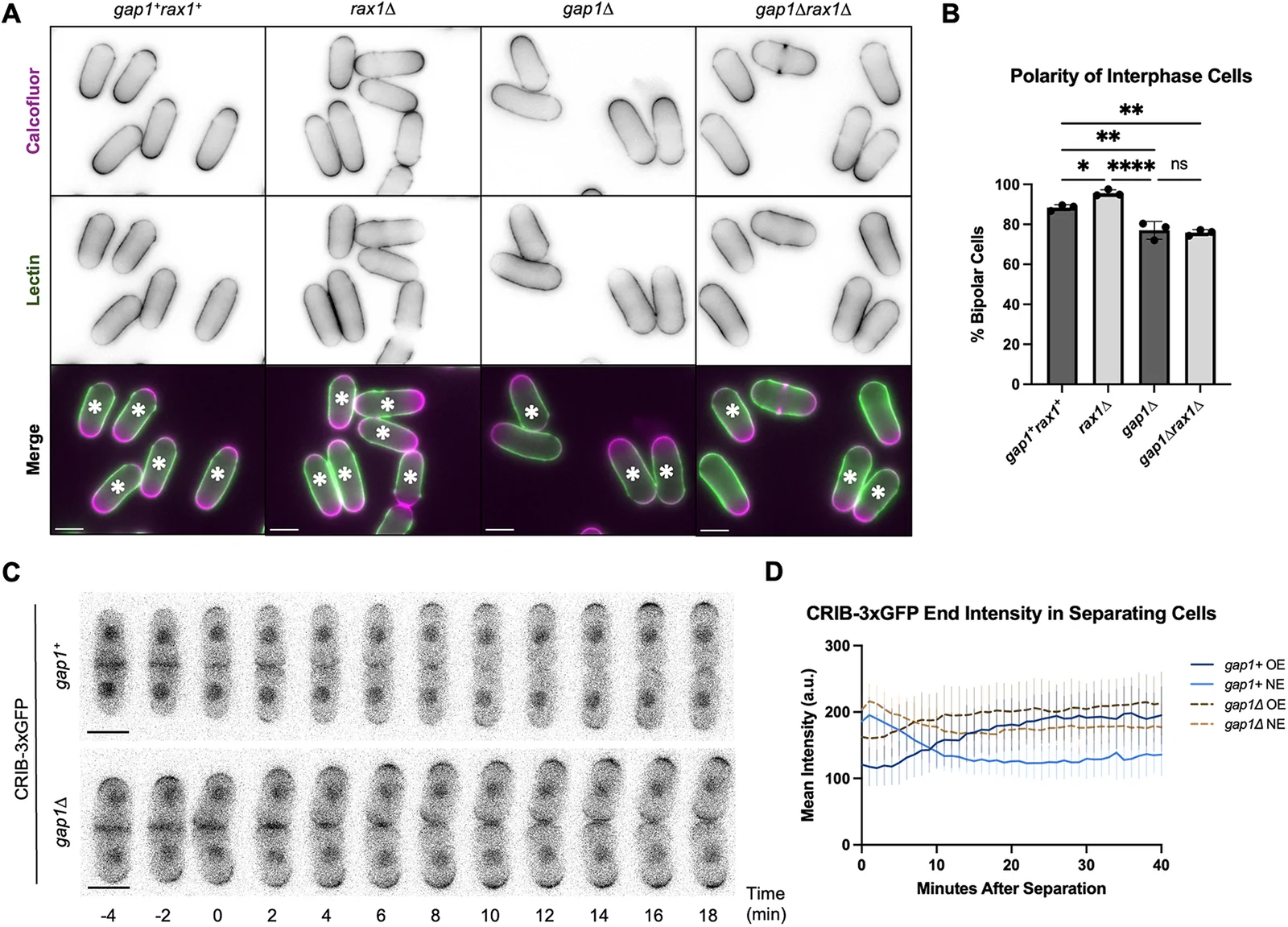
**Loss of Ras1 inhibition strengthens old-end dominance.** (A) Polarized growth of cells stained with lectin–Alexa Fluor 488 and Calcofluor White in the indicated strains. Asterisks mark bipolar cells. (B) Percentage of interphase cells displaying bipolar growth as determined by Calcofluor and lectin staining. Each point corresponds to the mean of an independent experiment (*n*≥153, *N*=3). Significance was determined by one-way ANOVA followed by Tukey's multiple comparisons test. (C) Montage of CRIB–3×GFP in separating *gap1^+^* and *gap1*Δ cells. Time ‘0’ is onset of cell separation. (D) Quantification of mean and s.d. of CRIB–3×GFP intensity over time at the old ends (OE) and new ends (NE) of separating *gap1^+^* and *gap1*Δ cells (*n*≥52 cells imaged across three independent experiments). Scale bars: 5 μm. Error bars are mean±s.d. a.u., arbitrary units. ns, not significant; *P<0.05; **P<0.01; ****P<0.0001.

Finally, we investigated how disrupting Ras1 activity through deletion of either *efc25* or *gap1* affects the distribution of Cdc42 activity in interphase cells. If old-end dominance is decreased when Ras1 activity is lost, we would expect that Cdc42 activity would be more symmetrically distributed between the two ends, similar to what we observed in *rax1*Δ cells. We found that CRIB–3×GFP intensity was more evenly divided between the two ends of *efc25*Δ cells than in the wildtype, with the old end no longer having a significantly higher fraction of intensity than that in the new end (Fig. 8A,B,G). We asked whether the symmetric distribution of CRIB–3×GFP was due to the low levels of active Cdc42 in these cells as Ras1 promotes Scd1 activity (Lamas et al., 2020) (Fig. 8C). Thus, we compared the distribution of active Cdc42 in *efc25*Δ cells with that in *scd2*Δ cells. The scaffold Scd2 is required for Scd1 recruitment and its loss decreases Cdc42 activation at the cell ends (Bendezú et al., 2015; Chang et al., 1994; Kelly and Nurse, 2011; Lamas et al., 2020). Despite similar decreases in total active Cdc42, *scd2*Δ cells displayed a highly asymmetric distribution of CRIB–3×GFP, indicating that the increased fraction of Cdc42 activity at the new end is specific to *efc25*Δ and not an artifact of less-available Cdc42–GTP (Fig. 8A–C). In addition, when Ras1 activity was increased through the loss of *gap1*, Cdc42 activity became more asymmetrically distributed (Fig. 8D,E,G). Although total Cdc42 activity at cell ends was not significantly different in *gap1*Δ cells, the fraction of activity at the old end was increased compared to that in wildtype cells (Fig. 8E,F). Therefore, our data indicate that Ras1 activity promotes old-end dominance, as this dominance decreases when Ras1 activation is lost and increases when its inhibition is removed.

**Fig. 8.**
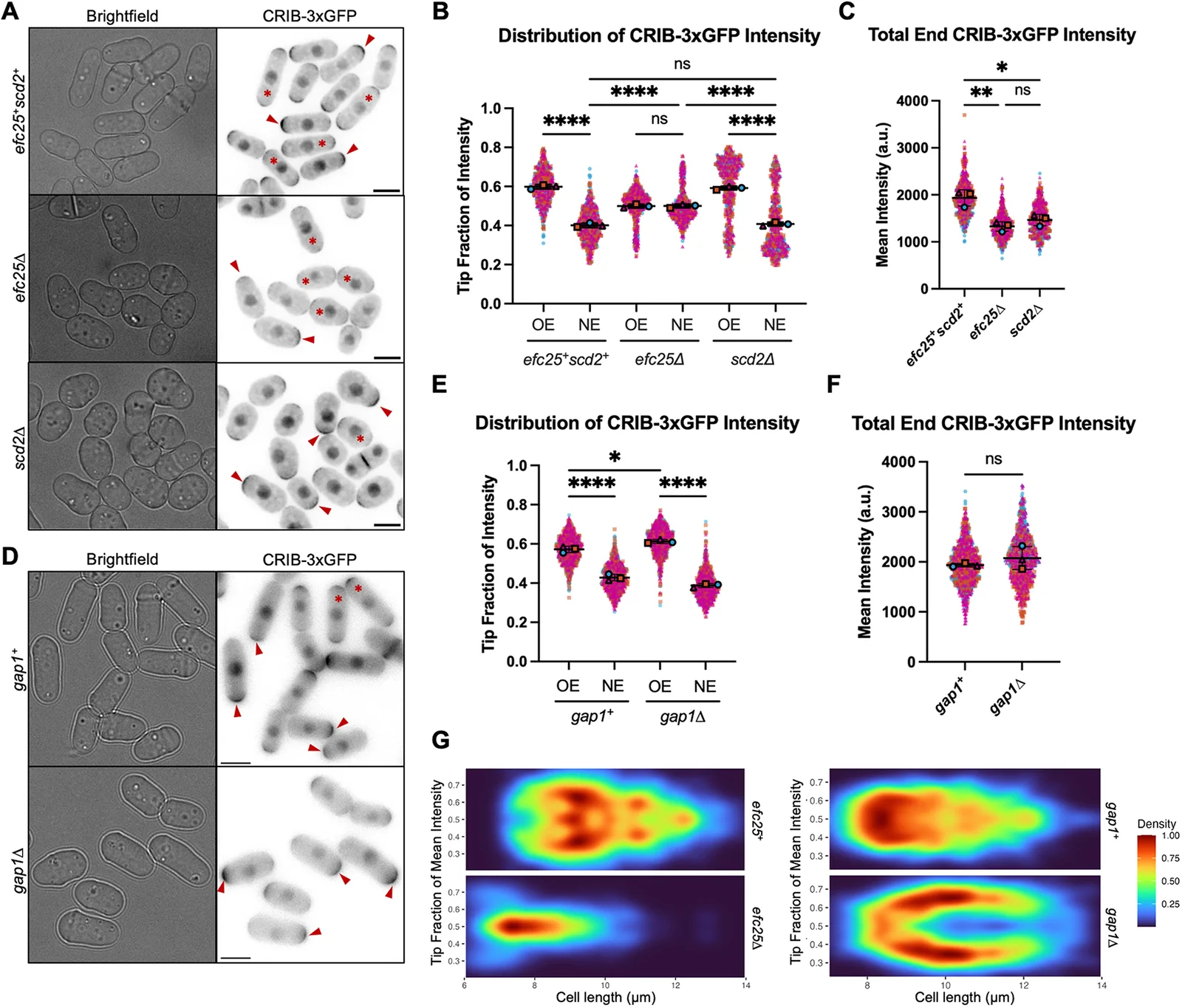
**Changes in Ras1 activity alter the distribution of Cdc42 activation between the two ends.** (A) Localization of CRIB–3×GFP in interphase cells of the indicated strains. Images are sum projections of *z*-series. Asterisks mark cells with similar levels of CRIB–3×GFP at both ends, whereas arrowheads mark examples of cells with asymmetric CRIB–3×GFP distribution. (B) Quantification of the fraction of total CRIB–3×GFP tip intensity at the old end (OE) and new end (NE). Outlined points represent the mean from one independent experiment, with small points corresponding to individual cells (*n*≥147 cells, *N*=3). Significance was determined by one-way ANOVA followed by Šídák's multiple comparisons test. (C) Quantification of the total CRIB–3×GFP intensity at the cell ends. Outlined points represent the mean from one independent experiment, with small points corresponding to individual cells. Significance was determined using an unpaired, two-tailed *t*-test with Welch's correction. (D) Localization of CRIB–3×GFP in *gap1^+^* and *gap1*Δ interphase cells. Asterisks mark cells with similar levels of CRIB–3×GFP at both ends, whereas arrowheads mark examples of cells with asymmetric CRIB–3×GFP distribution. (E) Quantification of the fraction of total CRIB–3×GFP tip intensity at the old ends (OE) and new ends (NE) of *gap1^+^* and *gap1*Δ cells. Outlined points represent the mean from one independent experiment, with small points corresponding to individual cells (*n*≥217 cells, *N*=3). Significance was determined by one-way ANOVA followed by Šídák's multiple comparisons test. (F) Quantification of the total CRIB–3×GFP intensity at *gap1^+^* and *gap1*Δ cell ends. Outlined points represent the mean from one independent experiment, with small points corresponding to individual cells. Significance was determined using an unpaired, two-tailed *t*-test with Welch's correction. (G) Heat maps of CRIB–3×GFP tip fraction versus cell length comparing *efc25^+^* versus *efc25*Δ (left, smoothed data, *n*≥488 cells collected over three independent experiments) and *gap1^+^* versus *gap1*Δ (right, smoothed data, *n*≥729 cells collected over three independent experiments). Plots were made using the ggplot2 package in R (Wickham, 2016). Note that the *x*-axis extends from 6 to 14 μm in the left plot, and from 7 to 14 μm in the right plot. Scale bars: 5 μm. Error bars are mean±s.d. a.u., arbitrary units. ns, not significant; *P<0.05; **P<0.01; ****P<0.0001.

## DISCUSSION

The delayed bipolar growth pattern in fission yeast occurs because immediately after division, the old end outcompetes the new end for Cdc42 activation. During cytokinesis, Cdc42 is inactivated at the cell ends and activated at the division site. However, just as the cells start to separate at the end of division, Cdc42 activity is redirected from the division site to the opposing end of the cell (Tatebe et al., 2008; Wei et al., 2016). Previous reports have shown that Cdc42 activation at the old end involves the cell cycle-dependent removal of an inhibitor along with the activation of protein synthesis via the NDR/Orb6 kinase pathway (Nuñez et al., 2016; Rich-Robinson et al., 2021; Verde et al., 1998). Although the mechanisms that regulate NETO to initiate the second growth site have been studied (Bohnert and Gould, 2012; Bohnert et al., 2020; Das et al., 2012; Glynn et al., 2001; Hercyk et al., 2019; Martin et al., 2005; Tatebe et al., 2005, 2008), those responsible for old-end dominance are not well understood. The loss of old-end dominance in cells that inherit non-growing ends suggests the presence of a memory of growth that allows the old end to outcompete the new end (Hercyk et al., 2019; Huisman and Brunner, 2011; Niccoli et al., 2003). Here, we identify the proteins Rax1/2 as memory-of-growth factors and demonstrate their role in promoting old-end dominance through activation of Ras1. Furthermore, we find that loss of this memory of growth delays activation of Cdc42 at the old ends of dividing cells, reducing their ability to outcompete the new end.

Rax1/2 are conserved across fungi and have been shown to regulate growth and polarity in several species. Rax1 and Rax2 interdependently promote bipolar budding in *S. cerevisiae* and *Yarrowia lipolytica*, and Rax1 has been shown to regulate growth and conidium development in *Aspergillus fumigatus* (Chen et al., 2000; Fujita et al., 2004; Igbalajobi et al., 2017; Kang et al., 2004; Li et al., 2025). Rax2 has also been shown to regulate both bud-site selection and direction of hyphal growth in *Candida albicans* (Gonia et al., 2013). Our results demonstrate that Rax1/2 regulate positioning of the site of polarization in fission yeast, supporting that this is a conserved function of Rax1/2 in fungi. Although Rax1 and Rax2 mark previous division sites in *S. cerevisiae*, thus preventing budding from a previous bud site (Chen et al., 2000; Fujita et al., 2004; Meitinger et al., 2014), in *S. pombe*, we find that they act as a memory of growth at the cell ends by localizing in a growth-dependent manner and remaining through cell division. We also find that Rax1/2 localization is primarily dependent on growth and remains during cytoskeleton disruption. To verify that Rax1/2 localization is indeed growth dependent, we analysed its localization in CK666-treated cells in a *sty1*Δ strain in which growth does not stall. However, the rate of growth was significantly reduced in these cells, thus making the intensity measurements challenging as the recovery signal was faint. However, the fluorescence recovery along the cell cortex, while faint, was distinct from the cytoplasmic signal in several cells. This, along with the continued localization of Rax2–GFP to the non-bleached growing cell ends, supports the notion that Rax2–GFP is able to localize to cell ends in the absence of branched actin. Our results demonstrate that delivery of Rax1/2 is dependent on active growth, as expected of memory-of-growth markers.

Consistent with reports in *S. cerevisiae* and *Y. lipolytica* (Kang et al., 2004; Li et al., 2025), we find that Rax1 and Rax2 colocalize and directly interact with each other to form a complex. Their interaction is likely required for their delivery to cell ends, as localization of Rax1 and Rax2 is interdependent. Our data show that without *rax1*, old-end dominance is decreased and cells become precociously bipolar. We find that loss of *rax1* weakens the advantage of the old end across different polarity mutants, including highly monopolar strains, demonstrating the importance of memory of growth to old-end dominance. However, the Rax1/2 complex does not appear to be the only mechanism promoting old-end dominance, as old-end growth still initiates first in *rax1*Δ cells, and deletion of *rax1* only partially rescues bipolar growth in monopolar mutants such as *scd2*Δ and *bud6*Δ (Table S1). This alternative pathway promoting old-end growth is likely Tea1 dependent, as deletion of both *rax2* and *tea1* causes both daughter cells to only grow from their new ends (Choi et al., 2006; Johnson, 2019). The transition from monopolar to bipolar growth is also cell cycle dependent. Cells arrested in G1 are mostly monopolar. Previous reports have shown that *rax2*Δ *cdc10-129* cells arrested in G1 show only a minor increase in bipolarity, indicating that *rax2*Δ does not regulate polarity due to a cell cycle defect (Choi et al., 2006). Indeed, our data with *rax1*Δ do not show a cell cycle or growth defect. It should be noted that previous work in *rax2*Δ cells showed decreased growth (Choi et al., 2006). Rax2 contains a much larger extracellular region than Rax1 that appears to be heavily glycosylated (Johnson, 2019); thus, it is possible that this difference reflects a *rax2*-specific role in cell growth, although additional research would be required to confirm this difference and identify the mechanism.

Here, we show that the Rax1/2 complex promotes old-end dominance by activating Ras1 at cell ends during division through recruitment of its GEF Efc25. This pathway is similar to the bud-site selection pathway in *S. cerevisiae*, wherein Rsr1 and its GEF Bud5 are recruited downstream of Rax1/2 (Bi and Park, 2012; Chiou et al., 2017; Kang et al., 2004). We find that Efc25 is recruited to dividing cell ends in a Rax1-dependent manner. However, whether the Rax1/2 complex interacts with other landmarks that play a similar role to Bud8/9 is not yet clear. Although the function and basic pathway of the Rax1/2 complex appear to be conserved, many of its interactors identified in *S. cerevisiae* lack homologs in fission yeast. Even Efc25 is not the ortholog of Bud5 but is instead more similar in sequence to Cdc25, a different Ras GEF in *S. cerevisiae* (Tratner et al., 1997). These differences suggest that the Rax1/2 complex might have adapted to interact with different proteins while maintaining its role as a memory of growth. Our results fail to show an interaction between Rax1/2 and Efc25, suggesting that Efc25 localization is regulated by Rax1/2 through an alternate mechanism. Indeed, even in *S. cerevisiae*, Bud5 is not directly recruited by Rax1/2 but rather via the recruitment of Bud8/9 (Kang et al., 2004; Krappmann et al., 2007). Further research is required to identify the mechanistic details of Rax1-dependent recruitment of Efc25.

Our results also demonstrate a role for Ras1 in initiating the site of polarization in fission yeast. Ras GTPases regulate the site of polarization in multiple fungal species, with the Ras-like GTPase Rsr1 also regulating bud-site selection in *C. albicans* and *Y. lipolytica* (Hausauer et al., 2005; Li et al., 2014). Rsr1 is also important to maintain the site and direction of polarized growth in fungal hyphae, as loss of Rsr1 leads to random branch initiation and curved hyphae in *C. albicans* and *Ashbya gosspyii* (Bauer et al., 2004; Hausauer et al., 2005)*.* Furthermore, Ras activity has been shown to determine the direction of cell migration in *Dictyostelium* and neutrophils (Kortholt et al., 2013; Pal et al., 2023; Sasaki et al., 2004; Zhang et al., 2008). This suggests that initiation and maintenance of the site of polarization are a conserved function of Ras GTPases in eukaryotes.

We find that Rax1 regulates Efc25 localization and Ras1 activity primarily in dividing cells, as the level of active Ras1 at interphase cell ends does not change with loss of *rax1*. The timing of Ras1 activation at the old end of dividing cells that we observe is similar to that of the reactivation of the NDR/Orb6 kinase pathway after cytokinesis (Gupta et al., 2014). Previous work has found that Orb6 regulates Ras1 activity by inhibiting translational repression of Efc25 (Chen et al., 2019; Nuñez et al., 2016). Rax1-dependent recruitment of Efc25 might coincide with de-repression of its translation at the end of cell division through increased Orb6 activity, ensuring that newly synthesized GEF is targeted to the old end. Our results suggest that rather than the total level of Efc25 at the old end determining old-end dominance, the direction and timing of Efc25 recruitment might be an important factor, as we do not observe any effect on bipolarity from overexpression of Efc25 alone.

Ras1 has previously been shown to promote polarization of Cdc42 through recruitment and activation of Scd1 (Chang et al., 1994; Lamas et al., 2020). The Ras1–Cdc42 morphogenesis pathway is also conserved in another fission yeast, *Schizosaccharomyces japonicus*, which requires Ras1, Efc25 and the Cdc42 pathway for proper cell shape and hyphal development (Nozaki et al., 2018). We find that in cells lacking Ras1–GTP at the ends of dividing cells, Cdc42 activation is delayed and the competition between the two ends is shifted, decreasing the dominance of the old end. With a weaker old end, the new end can activate Cdc42 and initiate growth earlier, leading to the two ends becoming more equal. In contrast, increasing Ras1 activity leads to early Cdc42 activation at cell ends during division, strengthening old-end dominance. Interestingly, growth rate is not directly correlated with the amount of Cdc42–GTP at the end, as there was no significant change in Cdc42 activity at the old end despite the lower growth rate in *rax1*Δ cells. This is consistent with other work that found that although increased Cdc42 activity correlated with increased growth, the speed of growth varied across cell ends with similar levels of active Cdc42 (Taheraly et al., 2020). Although we are confident in our categorization of the new end by its proximity to the birth scar (Mitchison and Nurse, 1985), these measurements were not performed in timelapse images in which the old and new ends could be identified with full certainty. However, we continued to see a more equal distribution of Cdc42–GTP and Scd2 in *rax1*Δ cells when the distribution was plotted without categorization of old versus new ends, supporting our findings that old-end dominance is lost in this mutant. Our results suggest that in the presence of competition between the cell ends, the timing of growth is a significant factor in determining the growth rate of each end, with a delay in onset of growth decreasing the maximal growth rate of that end. This pattern can be seen even in the natural variation of growth among wildtype fission yeast cells, with the old-end growth rate increasing as NETO is delayed (Taheraly et al., 2020). The increased monopolarity of many cytokinetic mutants is also consistent with these findings, as the competitive advantage of the old end increases while new-end growth is inhibited by incomplete division (Bohnert and Gould, 2012).

While *S. pombe* demonstrates a preference for initiating growth at ends with an inherited history of growth, the biological significance of this pattern is unclear. Precocious bipolarity has been associated with decreased cell wall integrity, suggesting that early growth at the recently divided end weakens the cell wall (Koyano et al., 2015). Another possibility is that old-end dominance is beneficial under low-nutrient conditions. The timing of NETO is altered in different media, with new-end growth occurring later in minimal medium compared to that in medium supplemented with yeast extract (Mitchison and Nurse, 1985). Recent work in our laboratory has found that stress response pathways prevent precocious bipolarity and that these pathways are downregulated in the transition to bipolar growth (Pathak et al., 2026). Furthermore, during growth in high cell densities and decreased nitrogen availability, *S. pombe* can form pseudohyphal invasive filaments that are highly monopolar at the ends (Amoah-Buahin et al., 2005; Dodgson et al., 2010). Fission yeast mutants that are more monopolar due to a failure to initiate NETO show more invasive pseudohyphal growth, whereas cells with premature NETO are less able to form pseudohyphae (Bohnert and Gould, 2012; Bohnert et al., 2020). Initiating growth at the old end might bias growth outwards, away from other cells and potentially towards fresh nutrient sources. Persistence in the direction of migration has been shown to allow chemotaxing cells to search a larger area with higher efficiency (Li et al., 2008). Directed outward growth of filaments might function in a similar way to allow for broader exploration of the environment.

The ability to retain a memory of polarity after it is altered or lost is found across eukaryotic life in many different forms. This memory allows cells to maintain correct polarization through changes in development and the surrounding environment. Even with cell rounding, cortical landmarks can remain asymmetrically localized during division, allowing for positioning of the mitotic spindle, determining cell fate or re-establishing polarity based on the shape of the mother cell (Akanuma et al., 2016; Boubakar et al., 2017; Minc and Piel, 2012; Théry et al., 2005). Memory is also important in migrating cells, allowing for directed migration towards a chemical signal even during fluctuations or temporary loss of the signal (van Haastert, 2021; Prentice-Mott et al., 2016; Skoge et al., 2014). Our work characterizes how fission yeast utilizes Rax1/2 as a landmark to reinitiate polarized growth at sites where growth has previously occurred. Through this memory of growth, Ras1 and Cdc42 are activated at the old end early in cell separation, ensuring its advantage over the new end and maintaining cell shape from one generation to the next.

## MATERIALS AND METHODS

### Strains and cell culture

The *S. pombe* strains used in this study are listed in Table S2. All strains are isogenic to the original strain PN567. Standard methods for genetic manipulation and analysis were used (Moreno et al., 1991). Cells were grown exponentially in rich yeast extract (YE) medium (Sunrise Science, 2011) at 25°C unless otherwise specified. To express constructs containing the *nmt41* promoter, cells were grown in Edinburgh minimal medium (EMM) (Sunrise Science, 2005) with added adenine and uridine (225 μg/ml). Cultures were grown for at least three rounds of eight generations before imaging.

### Strain construction

Deletion of *rax1* was performed as described previously (Krawchuk and Wahls, 1999). Primers were designed to amplify flanking regions of homology 270 bp immediately upstream of the start codon (forward, 5′-GTATCAAGTAAAGCTTACTA-3′; reverse, 5′-TCAGACAACCACCTCAAGAC-3′) and 250 bp immediately downstream of the stop codon (forward, 5′-CCTGTCGATTTCGTTCCATT-3′; reverse, 5′-CAAAACAGTATAGAATGATT-3′) of *rax1* (*SPAC23G3.05c*). Coding sequences for all genetic manipulations of *S. pombe* were obtained from PomBase (Rutherford et al., 2024; Wood et al., 2012). The reverse primer for the 5′ flanking region and the forward primer for the 3′ flanking region also contained pFA6a-specific sequences as described previously (Krawchuk and Wahls, 1999). The megaprimers were then used to amplify *kanMX* from the pFA6a-kanMX vector (Bähler et al., 1998) and the product was transformed into wildtype and *gap1*Δ cells. Deletion of *rax1* was confirmed by PCR.

C-terminal tagging of Rax2 with GFP and mScarlet was performed by PCR amplification of 491 bases of the *rax2* coding sequence directly upstream of the stop codon using primers with a 5′ overlap homologous to the pFA6a vector (GFP forward, 5′-GCCAGCTGAAGCTTCTGTTGAACTCTGAAGGG-3′; GFP reverse, 5′-TTAATTAACCCGGGGATCCGCTGGGTCTTTAATTCTTTTA-3′; mScarlet forward, 5′-GAACGCGGCCGCCAGTGTTGAACTCTGAAGGG-3′; mScarlet reverse, 5′-CGTTAATTAACCCGGGGCCTCCGCCCCCTCCACCGCCACCCTGGGTCTTTAATTCTTTTA-3′). The amplified fragments were then assembled with either pFA6a-GFP-kanMX digested with BsiWI and SalI or pFA6a-mScarlet-natMX digested with PvuII and BamHI using the NEBuilder HiFi DNA Assembly Cloning Kit (New England Biolabs, E5520S). The assembled plasmids were then linearized by digestion with SwaI and transformed into wildtype cells. To obtain a *sty1*Δ Rax2–GFP strain, a cross was performed using a *sty1*Δ strain containing *pREP4x-sty1+:ura4+* (JM1168, gift from James Moseley, Dartmouth College, NH, USA). Positively selected colonies were re-streaked on YE to promote loss of the plasmid, which was confirmed by plating on EMM lacking uracil.

N-terminal tagging of Rax1 with mNG was performed using the CRISPR/Cas9 method described in Fernandez and Berro (2024). The CRISPR target site and 20 bp guide RNA sequence (reverse strand, 5′-AACTTCTGAAACTCTCGGGG-3′) near the start of the *rax1* coding sequence were found using CRISPRdirect (Naito et al., 2015). The donor DNA sequence consisted of regions of homology directly upstream and downstream of the *rax1* coding sequence start codon with point mutations in the PAM and gRNA sequence flanking the coding sequence of mNG, along with a 5-glycine linker. pJB166 (Addgene #86998, deposited by Julien Berro) digested with CspCI, the assembled fragment containing the gRNA sequence with flanking regions homologous with pJB166, and the donor DNA were transformed into a *fex1*Δ *fex2*Δ strain (JB355, a gift from Julien Berro, Yale University, CT, USA). Successful N-terminal tagging was confirmed by PCR. The resulting mNG–Rax1 strain was then crossed to obtain a *fex1^+^ fex2^+^* mNG–Rax1 strain that was used for future experiments.

C-terminal tagging of Efc25 with mScarlet was performed by PCR amplification of 630 bases of the *efc25* coding sequence directly upstream of the stop codon using primers with 5′ overlap homologous to the pFA6a vector (forward, 5′-GAACGCGGCCGCCAGTTTAGAAGCTGGGTTAGTCG-3′; reverse, 5′-CGTTAATTAACCCGGGGCCTCCGCCCCCTCCACCGCCACCAGAAGAACGAGGTTCAAGTG-3′). The amplified fragment was then assembled with pFA6a-yomRuby3-natMX (a gift from James Moseley, Dartmouth College, NH, USA) digested with PvuII and AscI to remove the yomRuby3 sequence, and the mScarletI coding sequence amplified from pFA6a-mScarletI-kanMX (Addgene #136690, deposited by Julien Berro) (forward, 5′-CGGATCCCCGGGTTAAT-3′; reverse, 5′-CGCTTATTTAGAAGTGGCG-3′) by NEBuilder HiFi DNA Assembly. The assembled plasmid was linearized by MfeI and transformed into wildtype and mNG–Rax1 cells.

To overexpress *efc25*, the vector pJK148-nmt41-mNeonGreen-Efc25-leu1+ was constructed and packaged by VectorBuilder (vector ID: VB240703-1004qpp). The plasmid was linearized with Bsu36I and integrated into the *leu1-32* locus of *efc25*Δ cells.

### Microscopy

Unless otherwise stated, microscopy was performed at room temperature using a Zeiss AxioObserver microscope with an integrated Yokogawa spinning disk (Yokogawa CSU-X1 A1 spinning disk scanner) and a 100×/1.49 NA objective. Images were acquired with a Teledyne Photometrics Prime 95b back-illuminated sCMOS camera (serial no. A20D203014) using SlideBook (3i Intelligent Imaging Innovations).

Imaging of Calcofluor staining, Rax2–GFP in Fig. S2B and *gap1*Δ CRIB–3×GFP in Fig. 8D–G, and timelapse imaging of CRIB–3×GFP, Scd2–GFP and RasAct^GFP^ was performed using a Nikon Ti2 Eclipse wide-field microscope with a 100×/1.49 NA objective with fluorophores excited using an AURA Light Engine system (Lumencor). Images were acquired with an ORCA-FusionBT digital camera (Hamamatsu, model C15440-20UP, serial no. 500428) using Nikon NIS Elements (Nikon).

Brightfield imaging of cells for growth analysis was performed using an Olympus IX83 microscope with a VTHawk two-dimensional array laser-scanning confocal microscopy system (Visitech International) and a 100×/1.49 NA UAPO lens (Olympus). Images were acquired with a Hamamatsu electron-multiplying charge-coupled device digital camera (Hamamatsu EM-CCD Digital Camera ImageM, model C9100-13, serial no. 741262) using MetaMorph (Molecular Devices).

### Calcofluor staining

To visualize cell wall growth, cells were stained in YE liquid containing 50 μg/ml of Calcofluor White (18909, Sigma-Aldrich) at room temperature. Cells were categorized as monopolar or bipolar based on the intensity of staining at the ends and the position of the most recent birth scar. Dividing cells, identified by the presence of a septum stained by Calcofluor, were excluded from analysis. For Figs 6 and 7, cells were first stained with 5 μg/ml lectin from *Griffonia simplicifolia* (Isolectin GS-IB_4_-Alexa Fluor 488, I21411, Invitrogen), washed three times, then grown for an additional 45 min before Calcofluor staining and imaging. Bipolar cells were identified based on the intensity of Calcofluor staining and the relative decrease in lectin at the cell ends.

### Growth analysis

To measure the timing of new-end growth and the growth rate at each end, cells were imaged for 8 h with an interval of 2 min to capture multiple generations. Cells were plated on 35 mm glass-bottomed dishes (MatTek) overlaid with YE containing 0.7% agarose and 100 μM ascorbic acid to prevent phototoxicity. To determine the growth rate, the total length of the cell and the length of the new end were measured at each time point. The birth scar was used as the reference point to measure new-end length. NETO was defined as the timepoint when new-end length began consistently increasing. Average growth rate was calculated by dividing the change in length from the onset of NETO to the final length of the cell before division by the amount of time elapsed. For Fig. 1G, the measurements of total, old-end and new-end growth were averaged by binning the data in 10-min bins and calculating the mean and standard deviation. To correct for varying cell cycle lengths, the time for each cell was scaled to the average generation time of the genetic background.

### FRAP imaging and analysis

To inhibit cell growth, cells were treated with YE liquid containing 100 μM CK666 (SML006, Sigma-Aldrich) dissolved in DMSO (317275, EMD Millipore) or 100 μM brefeldin A (B7450, Invitrogen) dissolved in DMSO for 30 min prior to imaging. Cells were treated with 1% DMSO as a control. After 30 min, cells were plated on 35 mm glass-bottomed dishes overlaid with YE containing 0.7% agarose, 100 μM ascorbic acid and the appropriate drug treatment or control. Cells were then imaged for 60 min at 1 min intervals. Rax2–GFP was imaged with 200 ms exposure and 50% laser power at 488 nm wavelength, and mNG–Rax1 was imaged with the same settings except using 70% laser power. After the third timepoint, a 10 pixel×20 pixel area (1.1 μm×2.2 μm) at one of the cell ends was bleached with 20% laser power for 5 ms with three repetitions (15 ms total). Acquisition then resumed until the end of the 60-min timelapse. For the experiments in Fig. S3, cells were imaged for 120 min at 2 min intervals, with 200 ms exposure and 40% laser power at 488 nm. After the third timepoint, a 20 pixel×30 pixel area (2.2 μm×3.3 μm) at one of the cell ends was bleached with 10% laser power for 5 ms with three repetitions (15 ms total). Intensity was measured using a 4-pixel-wide segmented line along the cell cortex at the tip of the cell, with the non-bleached cell end measured as the control. Full-scale normalization of fluorescence recovery data and calculation of the mobile fraction were performed using EasyFRAP-web (Koulouras et al., 2018).

### Fluorescence intensity image acquisition and analysis

Image analysis was performed in Fiji (Schindelin et al., 2012). For still images of fluorescent proteins, *z*-series of 16 slices at an interval of 0.4 μm were imaged. To measure localization of CRIB–3×GFP, Scd1–mNG, Scd2–GFP and RasAct^GFP^ at cell ends, images were sum projected and a cell-free region was used for background subtraction. Freehand regions of interest (ROIs) were drawn around the cell ends and the mean intensity was measured. The tip fraction was calculated as the mean intensity at each end divided by the total end intensity, which was the sum of the mean intensity at both ends of the cell. Assignment of the old and new end of each cell was determined by the location of the most recent birth scar. Interphase cells were identified by the presence of a single, non-dividing nucleus, and new daughter cells that had not fully separated and still overlapped at the new ends were not measured. Dividing cells were identified by the presence of a visible septum in brightfield images.

To measure the timing of mitosis and cell separation, timelapse images of Rlc1–tdTomato and Sad1–mCherry were taken as *z*-series of 11 slices at an interval of 0.6 μm every 2 min for up to 3 h. Spindle pole body distance was measured as a straight line between the two points of Sad1–mCherry localization. Ring constriction onset was determined by the start of decreasing diameter of the Rlc1–tdTomato-labeled ring. The length of cell separation was measured from the timepoint at which Rlc1–tdTomato was only a single point in the middle of the cell that was no longer constricting, to the timepoint at which separation of the daughter cells was evident due to a sudden increase in distance between the cell ends.

To examine mNG–Rax1 and Rax2–GFP localization when actin or microtubules were depolymerized, cells were treated with either 250 μM latrunculin B (428020, EMD Millipore) or 25 μg/ml MBC (378674, Sigma-Aldrich) for 30 min before imaging, with 1% DMSO as a control.

To measure Efc25–GFP and mNG–Efc25 intensity, the four middle slices of the *z*-series were sum projected and background subtraction was performed as described above. The mean intensity was measured using a 4-pixel-wide segmented line along the cell cortex from the edge of the division site, across the cell tip, to the opposite side. The centers of the plot profile from each end were then aligned and averaged by binning length into 0.1 μm intervals and calculating the mean and standard deviation for each bin. To account for the increase in total intensity in *rax1*Δ cells (Fig. 5G,H), intensity was normalized by calculating the mean intensity along the cell side between 3.5 and 5.5 μm from the cell tip for each individual end and subtracting it from the mean intensity, setting negative intensity values to zero. Graphs of the non-normalized data are included in Fig. S7. Division site intensity was measured using a 10 pixel (1.1 μm)-wide line through the middle of the cell. To calculate whole-cell intensity, the full *z*-series was sum projected, then cells were segmented using the Trainable Weka Segmentation Fiji plugin (Arganda-Carreras et al., 2017) and the mean intensity of each cell was measured.

For timelapse imaging of fluorescent proteins, images were taken of a single *z*-plane in the middle of the cell. Cells were plated on 35 mm glass-bottomed dishes overlaid with YE containing 0.7% agarose and 100 μM ascorbic acid, then imaged for 1 h with 1 min intervals. A cell-free region of the image was used for background subtraction. Freehand ROIs drawn at the cell ends and a polygon ROI surrounding the division site were used to measure mean intensity.

To measure CRIB–3×GFP oscillations, a region in the cytoplasm was used to perform a second background subtraction for each individual cell, then tip intensity was measured as previously described. The time intervals for each cell to be used for further analysis were limited to a continuous section where the intensity of each end fluctuated around a relatively stable mean. The cross-correlation coefficient of the two ends was then calculated using R (https://www.r-project.org/), and the period of oscillation was defined as the mean distance between the first two peaks of the cross-correlation function.

### FRET analysis

FRET efficiency was determined by measuring the increase in donor fluorescence after acceptor photobleaching. Cells were plated on 35 mm glass-bottomed dishes overlaid with YE containing 0.7% agarose and 100 μM ascorbic acid. A single *z*-plane image was acquired, then photobleaching was performed using 50% laser power at 565 nm wavelength for three repetitions of 6 ms each (18 ms total) in freehand ROIs drawn around whole cells. A final image was then acquired after photobleaching. To calculate FRET efficiency, 4-pixel-wide segmented line ROIs were drawn along the cell cortex at regions of colocalization pre-bleach and mean intensity was measured in the 488 nm channel in both images acquired before and after acceptor photobleaching. Unbleached cells in the same image were also measured as controls. Post-acceptor photobleaching intensities were corrected for photobleaching of the donor fluorophore. The percentage of FRET efficiency was calculated by dividing the initial donor fluorescence intensity by the post-bleach intensity, subtracting the result from 1 and multiplying by 100.

### Co-immunoprecipitation

Membrane fractionation was performed as described previously (Sieber et al., 2026). 50 ml of cells were lysed in lysis buffer [LB; 50 mM Tris-HCl pH 7.4, 200 mM NaCl, 1 mM EDTA, 100 μM PMSF, Halt protease and phosphatase cocktail (Thermo Fisher Scientific)] by six cycles of bead-beating at 5500 rpm for 45 s, with 2 min breaks on ice between cycles. Cell lysates were eluted by piercing the tubes, followed by collection into fresh tubes by centrifugation at 400 ***g*** at 4°C for 20 min. The membrane fraction was obtained by centrifugation at 21,000 ***g*** at 4°C for 1 h. The soluble fraction was transferred to a new tube and the membrane pellet was resuspended in LB containing 1% lauryl maltose neopentyl glycol (LMNG; A50940, Thermo Fisher Scientific) and 0.1% cholesteryl hemisuccinate (CHS; C6512, Sigma-Aldrich). Lysates were then cleared by centrifugation at 13,000 ***g*** at 4°C for 20 min.

Protein concentration was determined by Bradford assay and lysates diluted in LB containing 1% LMNG and 0.1% CHS to 1 mg/ml. Lysates were added to pre-washed GFP-Trap magnetic agarose beads (ChromoTek) and incubated on a rotor at 4°C for 1 h. Beads were washed three times in LB containing 1% NP-40 and eluted in 2× Laemmli sample buffer (Bio-Rad) with 5% β-mercaptoethanol heated at 95°C for 5 min.

### SDS-PAGE and western blotting

20 μg of protein from the input and flowthrough from co-immunoprecipitation were combined with equal volumes of 2× Laemmli sample buffer with 5% β-mercaptoethanol and denatured by heating at 95°C for 5 min. Samples were run on a 4–20% Mini PROTEAN TGX precast gel (Bio-Rad) in Tris–glycine–SDS buffer (Bio-Rad). Proteins were transferred to 0.45 μm PVDF membranes (Millipore). Blots were blocked in Intercept (TBS) Protein-Free Blocking Buffer (LICORbio). Primary antibodies were diluted in Intercept (TBS) Protein-Free Blocking Buffer with 0.2% Tween-20, with 0.02% SDS added to secondary antibody dilutions. TBS with 0.2% Tween-20 was used for washing. The primary antibodies used were: anti-GFP mouse monoclonal antibody (1:1000, Sigma-Aldrich, 11814460001, RRID:AB_390913) and anti-mNG rabbit monoclonal antibody (1:1000, Cell Signaling Technology, 55074, RRID:AB_3741755). The secondary antibodies used were: goat anti-mouse IRDye 680RD (1:10,000, LICORbio, 926-68070, RRID:AB_10956588) and goat anti-rabbit IRDye 800CW (1:10,000, LICORbio, 926-32211, RRID:AB_621843). Blots were imaged on an Odyssey M imaging system (LICORbio). Full western blots are included in Fig. S9.

### Data analysis and statistical tests

Data were processed using the tidyverse R package (Wickham et al., 2019). Statistical significance was calculated using GraphPad Prism. Unless otherwise specified, a two-tailed unpaired Student's *t*-test with Welch's correction was used when comparing two conditions. A one-way ANOVA corrected for multiple comparisons was used when comparing three or more conditions. Graphs were made in GraphPad Prism unless otherwise noted.

## Supplementary Material



10.1242/joces.264600_sup1 Supplementary information
