# Supplementary material for: Memory of growth promotes early Ras GTPase activation to establish dominance of inherited cell ends in Schizosaccharomyces pombe
Source: J Cell Sci. 2026 Oct 12;139(19):jcs264600. doi: 10.1242/jcs.264600 (PMC13741952; doi:10.1242/jcs.264600)
Supplement: Supplementary file 1 [file joces-139-264600-s1.pdf]

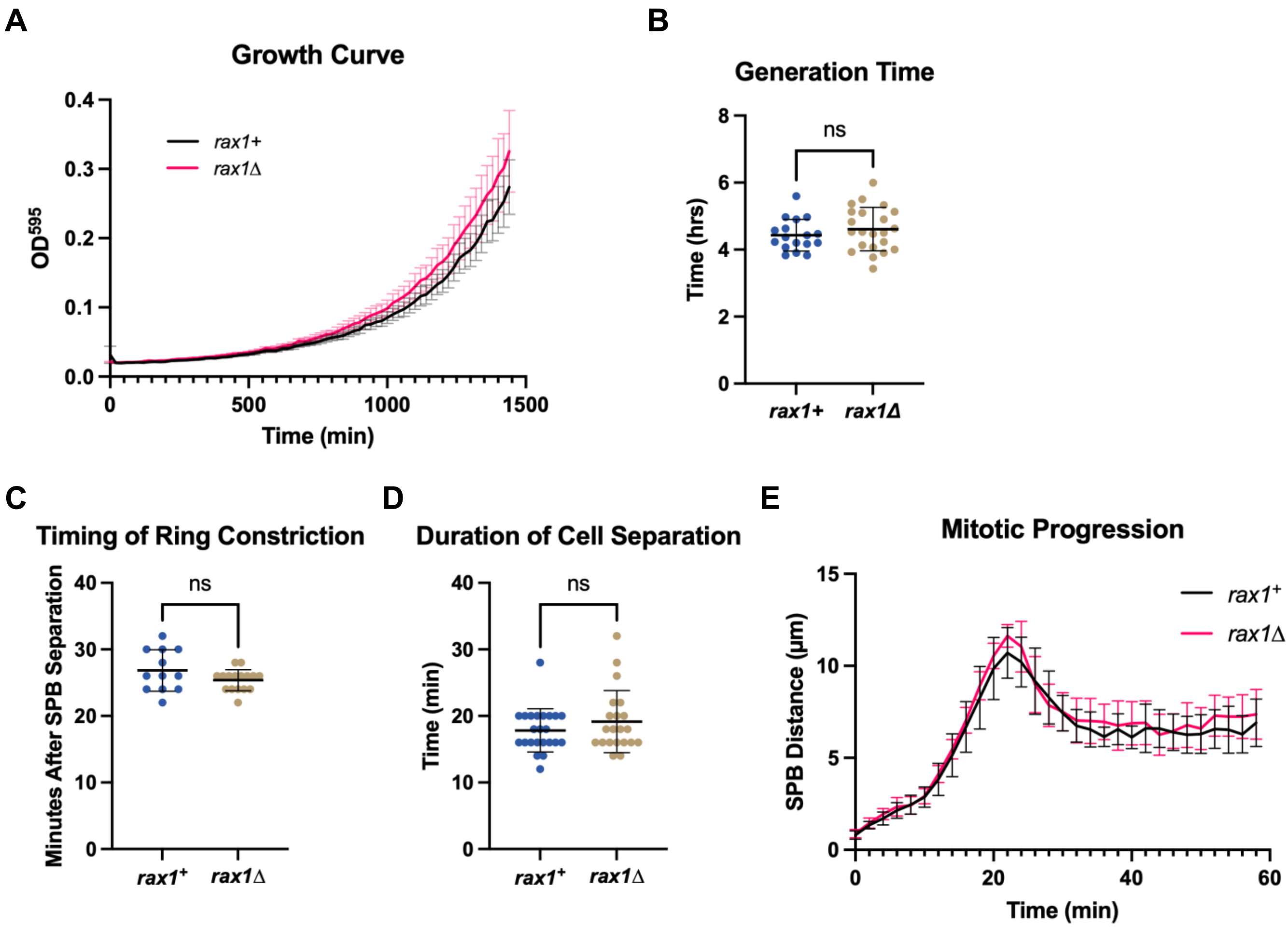

Fig. S1. Loss of *rax1* does not affect overall growth. (A) Growth curve of *rax1*+ and *rax1*Δ cell cultures. Cells were grown in YE in a 96-well plate and OD at 595 nm was measured every 20 minutes. Mean ± s.d. (n = 4). **(B)** Generation time of *rax1*+ and *rax1*Δ cells. Each point represents a cell imaged in one of three independent experiments (n ≥ 17). **(C)** Timing of onset of ring constriction from spindle pole body (SPB) separation. Each point represents a cell imaged in one of three independent experiments (n ≥ 12). **(D)** Timing of cell separation from the end of ring constriction. Each point represents a cell imaged in one of three independent experiments (n ≥ 21). **(E)** Distance between spindle pole bodies of mitotic cells over time. Time '0' is the onset of spindle pole body separation. (n ≥ 12). Error bars are mean±s.d. Significance was determined using an unpaired, two-tailed t-test with Welch's correction. ns = not significant.

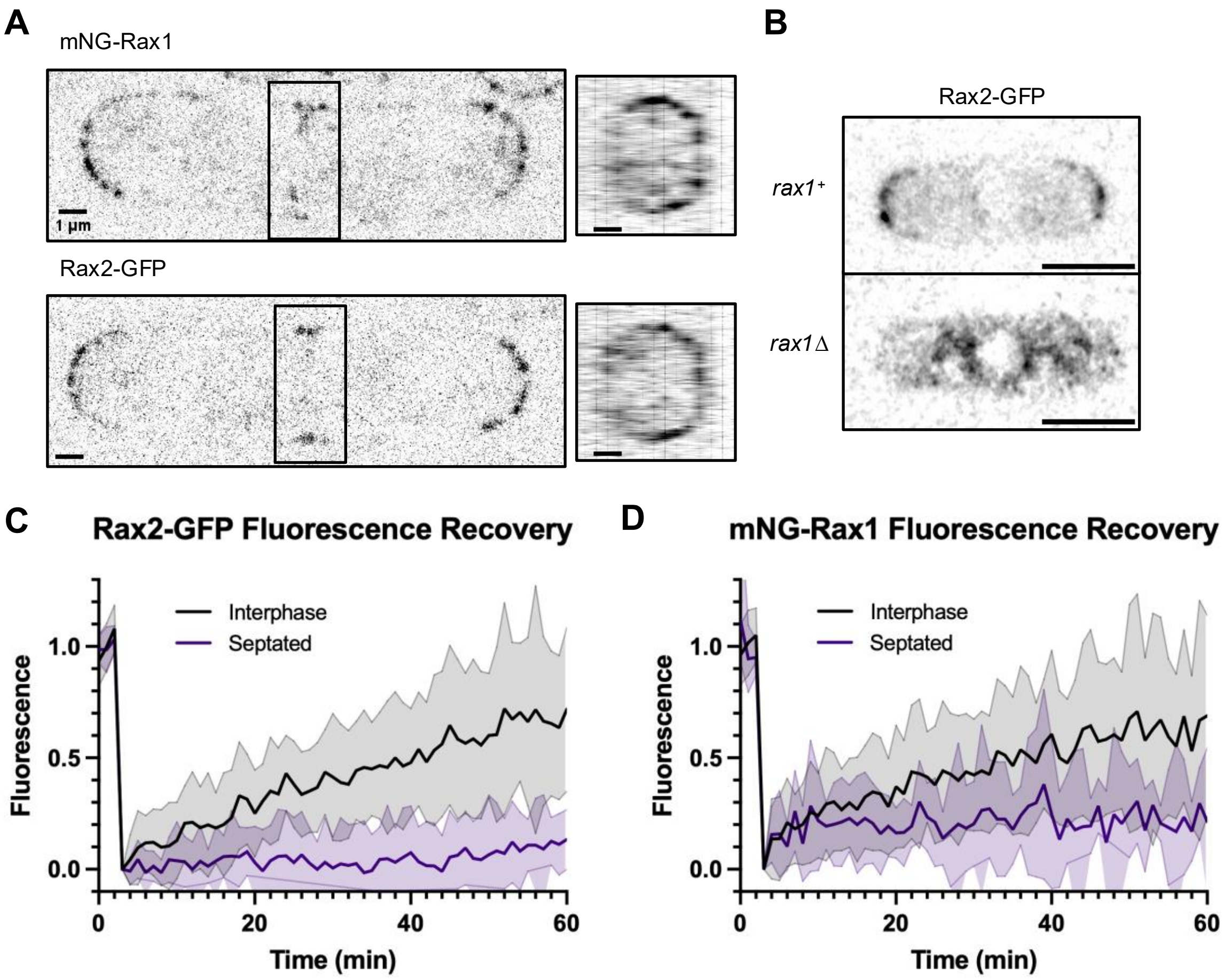

Fig. S2. Localization and fluorescence recovery of Rax1 and Rax2. (A) SoRa images of mNG-Ras1 and Rax2-GFP in dividing cells, with a 3D projection of the inset rotated 90º. 2.8x magnification. Scale bars are 1 µm. **(B)** Localization of Rax2-GFP in *rax1⁺* and *rax1Δ* cells. Scale bars are 5 µm. **(C,D)** Mean ± s.d. of normalized fluorescence recovery over time of Rax2-GFP (C) and mNG-Rax1 (D) in DMSO treated cells. (n ≥ 6 cells). Data from interphase cells is the same as the corresponding DMSO treated control in Fig. 2E,G.

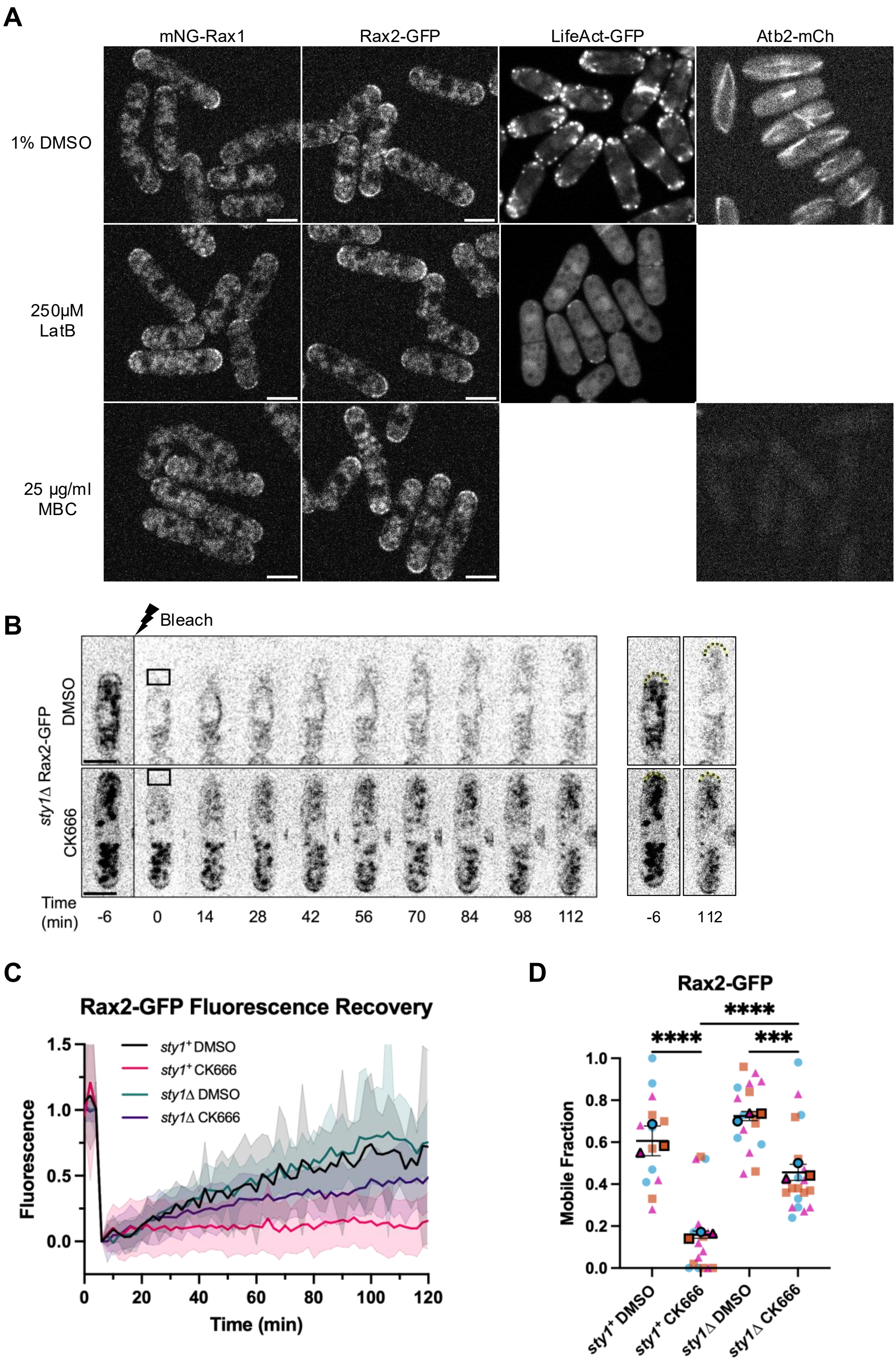

Journal of Cell Science • Supplementary information

Fig. S3. Rax1 and Rax2 continue to localize to cell ends during disruption of the cytoskeleton. (A) Localization of mNG-Rax1 and Rax2-GFP after a 30-minute treatment of DMSO (1%), Latrunculin B (250 µM), or MBC (25 µg/ml). LifeAct-GFP Atb2-mCh cells were also included to verify disruption of the cytoskeleton by drug treatment. **(B)** Montage showing fluorescence recovery after photobleaching of Rax2-GFP in *sty1*Δ cells treated with either DMSO (1%) or CK666 (100 µM). Time '0' is the first timepoint after photobleaching. Boxes show the photobleached region. Examples of the ROI used for intensity measurements are shown on the right. Scale bars are 5 µm. **(C)** Mean ± s.d. of normalized fluorescence recovery of Rax2-GFP over time in DMSO (1%) and CK666 (100 µM) treated cells. ($n \geq 13$ cells). **(D)** Quantification of the mobile fraction of fluorescence recovery of Rax2-GFP after 120 minutes. Outlined points represent the mean from one independent experiment while small points correspond to individual cells ($n \geq 4$ cells, $N = 3$). Error bars are mean±s.d. Significance was determined by one-way ANOVA followed by a Šídák's multiple comparison test, *** = $p < 0.001$, **** = $p < 0.0001$.

Journal of Cell Science • Supplementary information

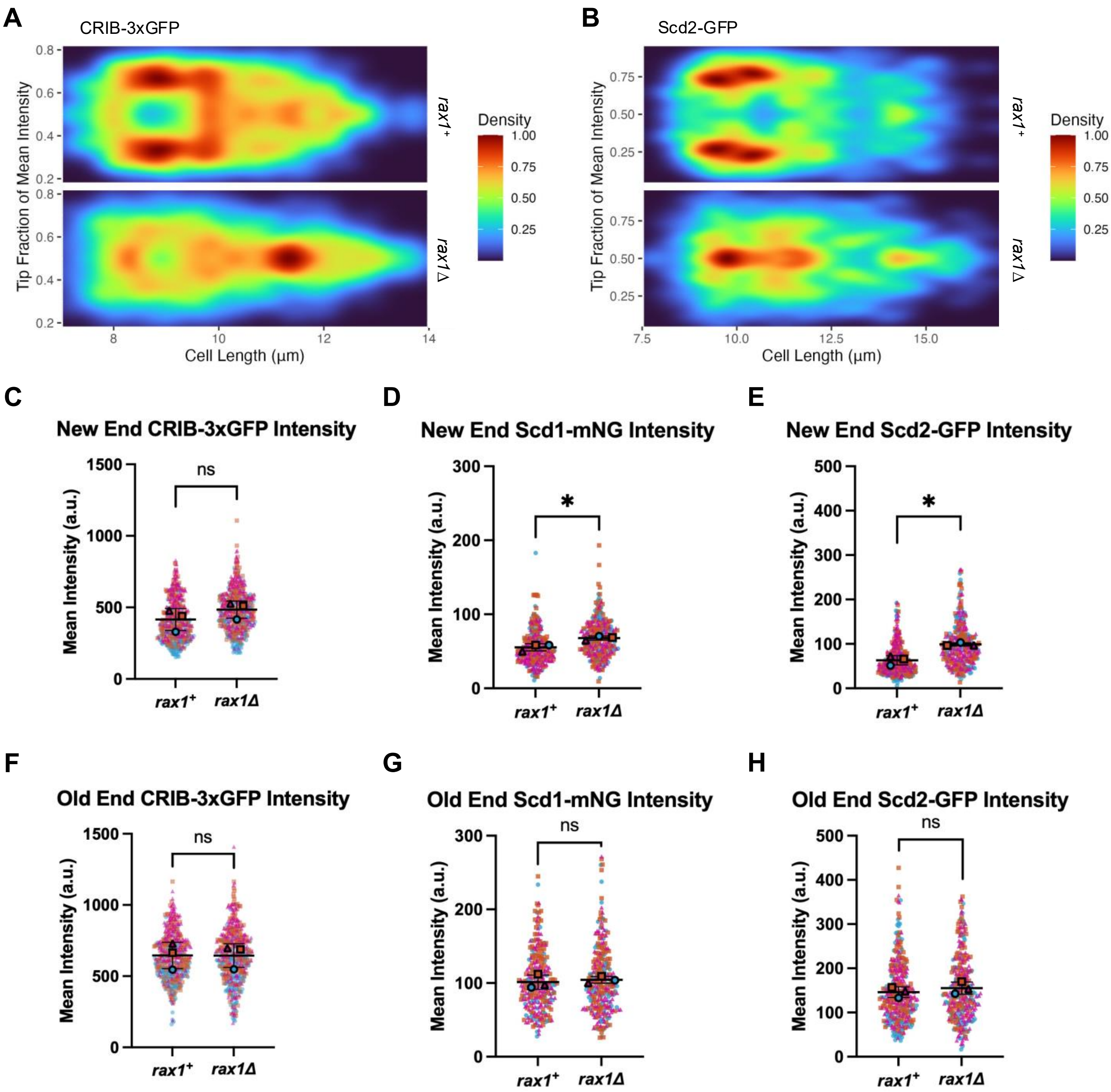

Fig. S4. Loss of *rax1* increases activation of Cdc42 at the new ends. (A) Heat-map of CRIB-3xGFP tip fraction versus cell length (smoothed data, n ≥ 486 cells). **(B)** Heat-map of Scd2-GFP tip fraction versus cell length (smoothed data, n ≥ 322 cells). Plots made using the ggplot2 package in R (Wickham, 2016). **(C-H)** Intensity of CRIB-3xGFP (C,F), Scd1-mNG (D,G), and Scd2-GFP (E,H) at the new ends (C-E) or old ends (F-H) of interphase *rax1⁺* and *rax1*Δ cells. Outlined points represent the mean from one independent experiment, with small points corresponding to individual cells (n ≥ 54 cells, N = 3). Error bars are mean±s.d. Significance was determined using an unpaired, two-tailed t-test with Welch's correction. * = $p < 0.05$, ns = not significant.

Journal of Cell Science • Supplementary information

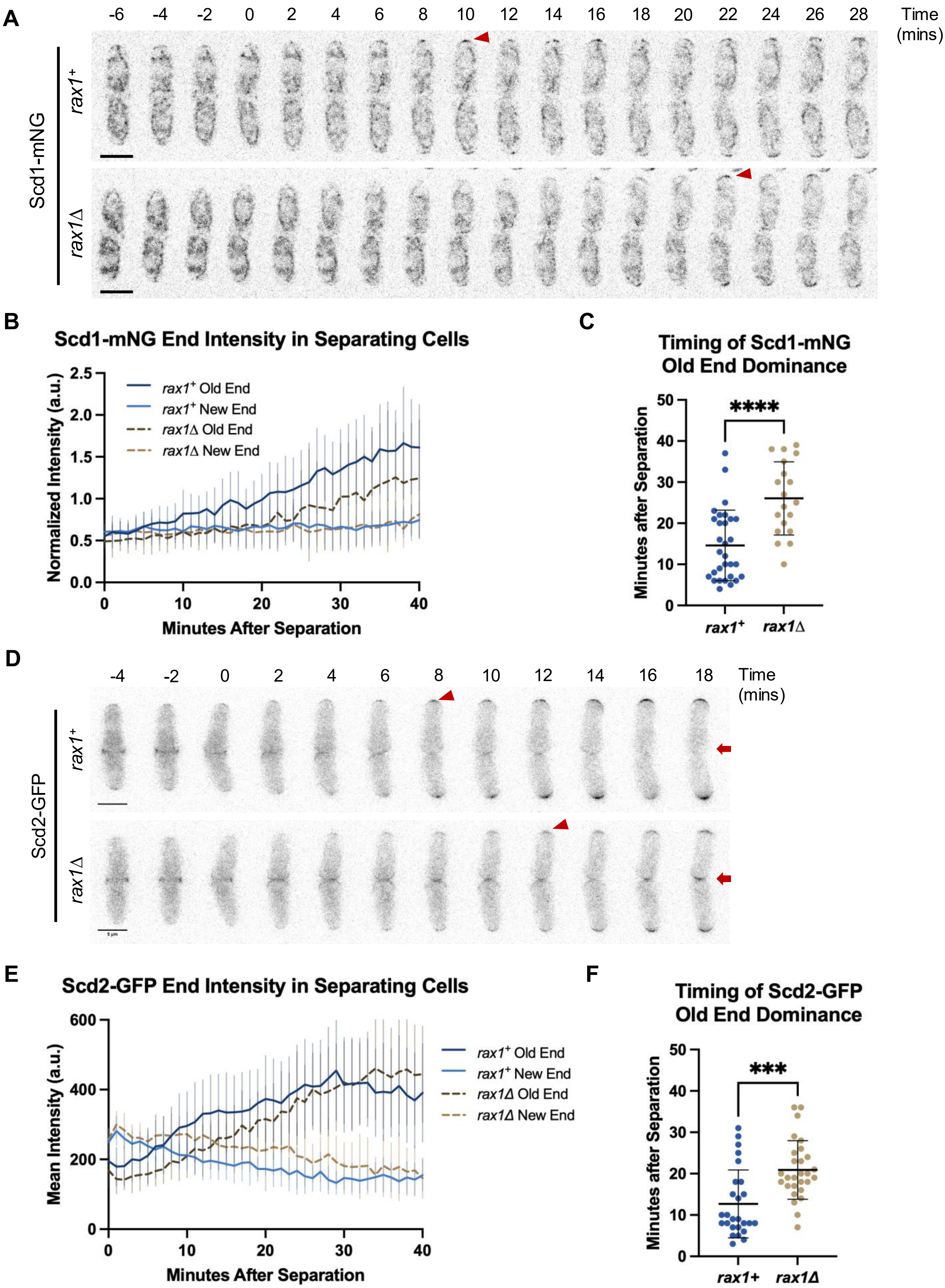
A
-6 -4 -2 0 2 4 6 8 10 12 14 16 18 20 22 24 26 28
Time (mins)
Scd1-mNG
rax1+
rax1Δ
B
Scd1-mNG End Intensity in Separating Cells
Normalized Intensity (a.u.)
Minutes After Separation
rax1+ Old End
rax1+ New End
rax1Δ Old End
rax1Δ New End
C
Timing of Scd1-mNG Old End Dominance
Minutes after Separation
****
rax1+
rax1Δ
D
-4 -2 0 2 4 6 8 10 12 14 16 18
Time (mins)
Scd2-GFP
rax1+
rax1Δ
E
Scd2-GFP End Intensity in Separating Cells
Mean Intensity (a.u.)
Minutes After Separation
rax1+ Old End
rax1+ New End
rax1Δ Old End
rax1Δ New End
F
Timing of Scd2-GFP Old End Dominance
Minutes after Separation
***
rax1+
rax1Δ

Fig. S5. Recruitment of Scd1-mNG and Scd2-GFP to the old ends after cell separation is delayed in *rax1*Δ cells. (A) Montage of Scd1-mNG in separating *rax1*$^{+}$ and *rax1*Δ cells. Arrowheads mark localization of Scd1-mNG at old ends. Time '0' is onset of cell separation. **(B)** Quantification of mean and s.d. of normalized Scd1-mNG intensity over time at the old and new ends of separating *rax1*$^{+}$ and *rax1*Δ cells ($n \geq 20$). Intensity was normalized by cytoplasmic intensity. **(C)** Quantification of the timepoint at which old-end Scd1-mNG intensity becomes higher than new-end intensity. Data points correspond to individual cells. **(D)** Kymographs of Scd2-GFP in separating *rax1*$^{+}$ and *rax1*Δ cells. Arrowheads mark localization of Scd2-GFP at old ends, while arrows mark the new ends. Time is in minutes from cell separation. **(E)** Quantification of mean and s.d. of Scd2-GFP intensity over time at the old and new ends of separating *rax1*$^{+}$ and *rax1*Δ cells ($n \geq 18$). **(F)** Quantification of the timepoint at which old-end Scd2-GFP intensity becomes higher than new-end intensity. Data points correspond to individual cells. Scale bars are 5 µm. Error bars are mean±s.d. Significance was determined using an unpaired, two-tailed t-test with Welch's correction. *** = $p < 0.001$, **** = $p < 0.0001$.

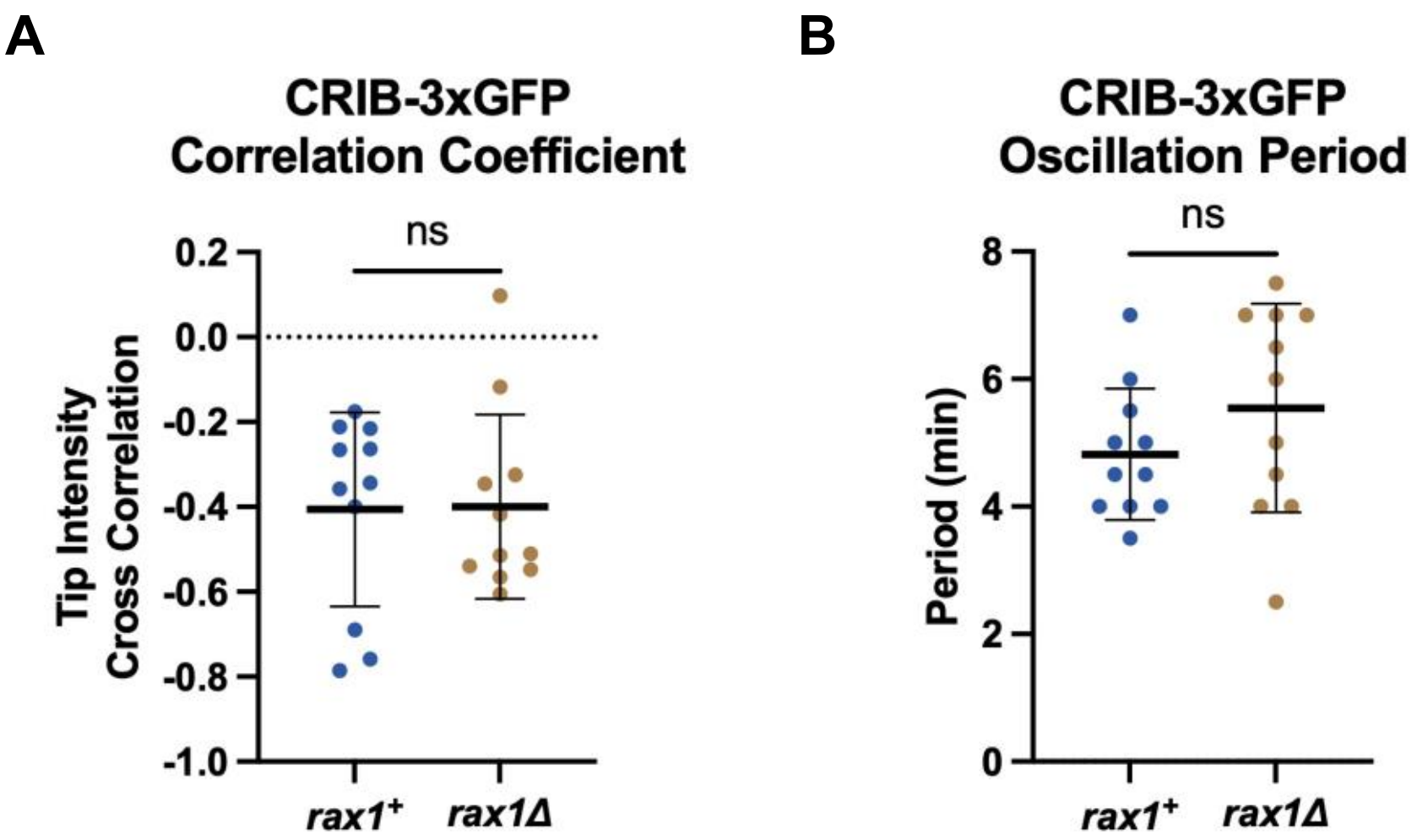

C

Strain	% Symmetric Oscillations
rax1^{+}	18.2%
*rax1*Δ	45.4%

Fig. S6. Loss of *rax1* leads to more symmetric anti-correlated oscillations of active Cdc42. (A) The cross-correlation coefficient of CRIB-3xGFP intensity over time at old and new ends in *rax1*$^{+}$ and *rax1*Δ cells. Data points correspond to individual cells (n = 11). **(B)** Quantification of the period between CRIB-3xGFP oscillations in *rax1*$^{+}$ and *rax1*Δ cells. Data points correspond to individual cells (n = 11). Significance was determined using an unpaired, two-tailed t-test with Welch's correction. Error bars are mean±s.d. **(C)** The percentage of cells with symmetric CRIB-3xGFP oscillations between the two ends (n = 11). Cells were classified as having symmetric oscillations if the ratio of the average intensity of the new end over the average intensity of the old end was between 0.5 and 1.5.

Journal of Cell Science • Supplementary information

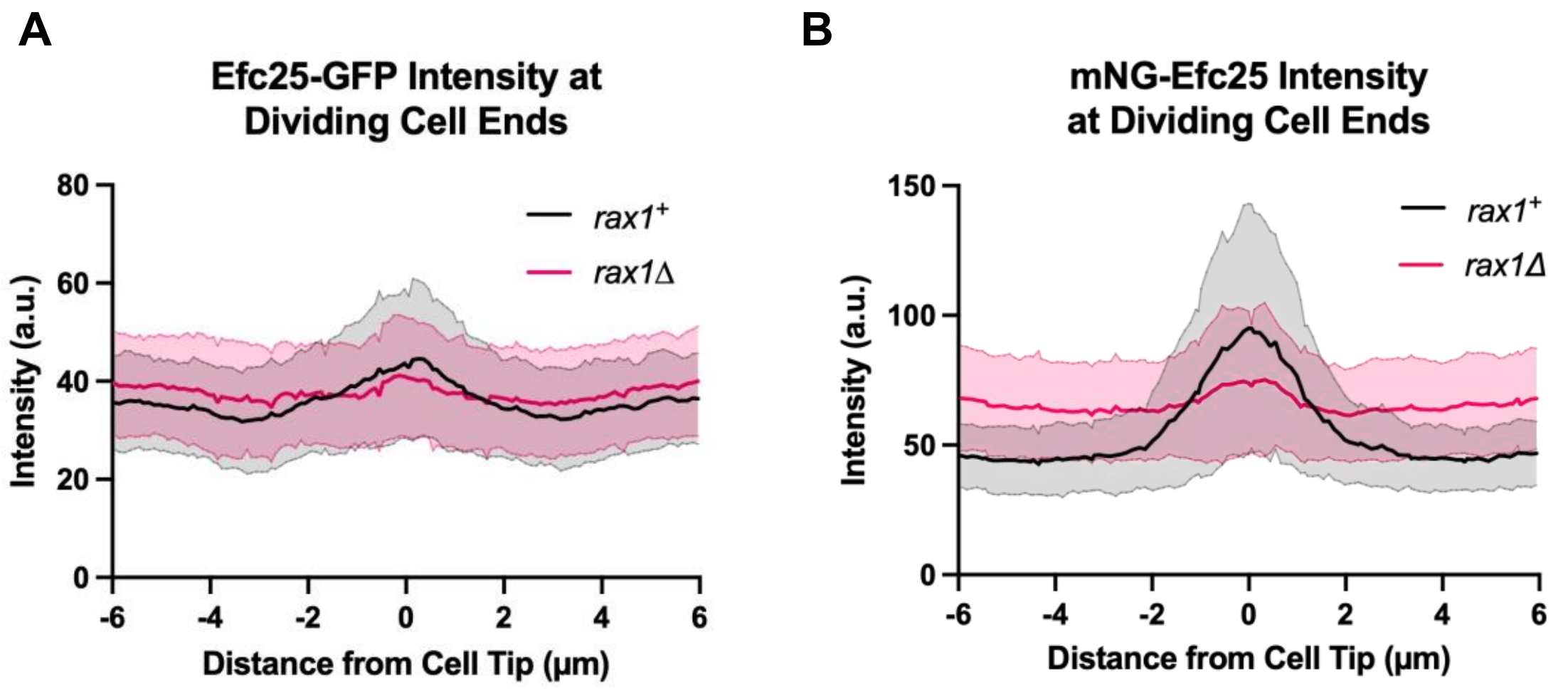

Fig. S7. Loss of *rax1* decreases localization of Efc25 to dividing cell ends. (A) Plot profile of the mean ± s.d. of Efc25-GFP intensity along the cell cortex in dividing cells (n ≥ 157). Non-normalized values of the data in Fig. 5B. **(B)** Plot profile of the mean ± s.d. of mNG-Efc25 intensity along the cell cortex in dividing cells (n ≥ 115). Non-normalized values of the data in Fig. 5E.

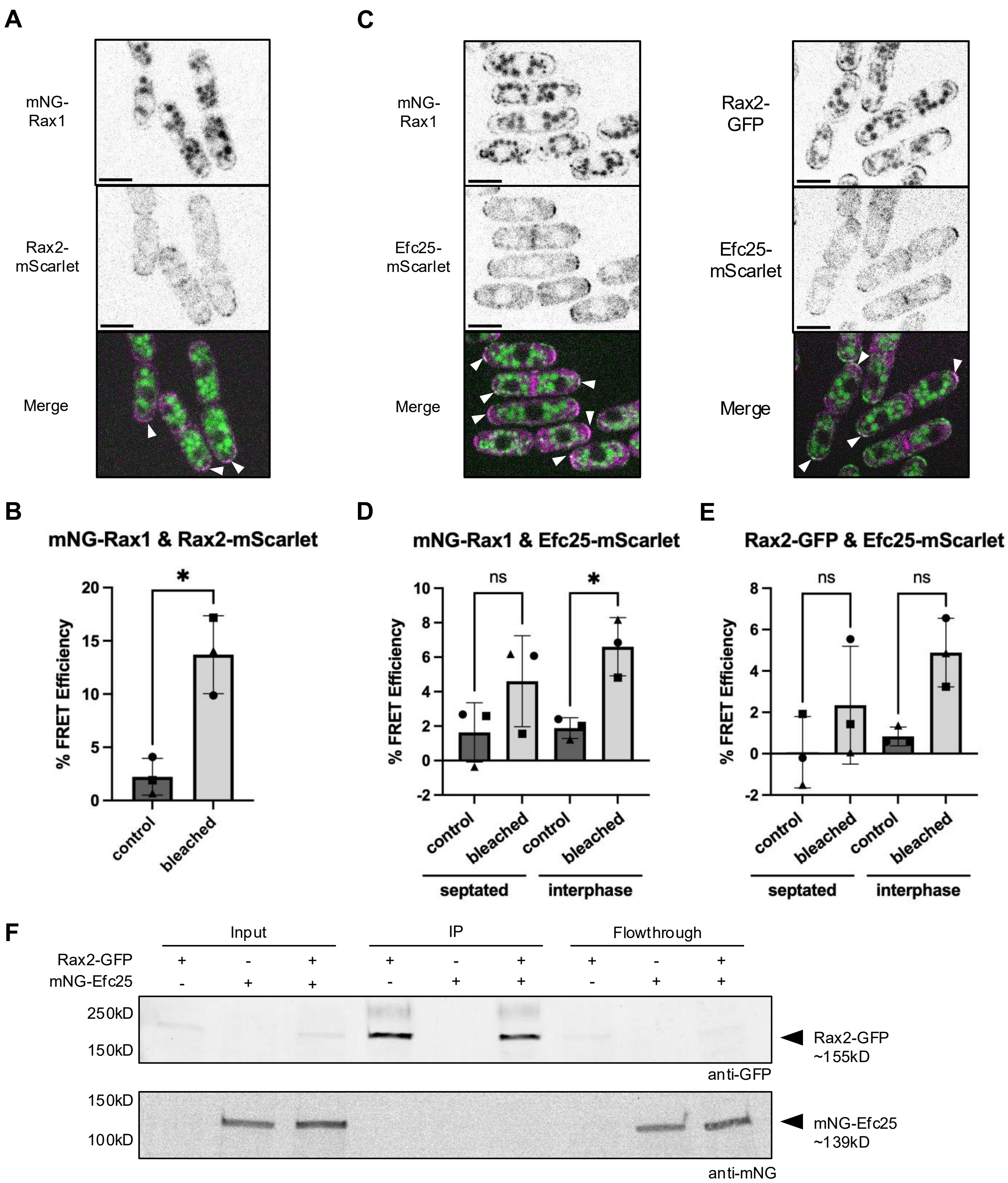
A
mNG-Rax1
Rax2-mScarlet
Merge
C
mNG-Rax1
Efc25-mScarlet
Merge
Rax2-GFP
Efc25-mScarlet
Merge
B
mNG-Rax1 & Rax2-mScarlet
% FRET Efficiency
20
15
10
5
0
*
control
bleached
D
mNG-Rax1 & Efc25-mScarlet
% FRET Efficiency
10
8
6
4
2
0
-2
ns
*
control
bleached
control
bleached
septated
interphase
E
Rax2-GFP & Efc25-mScarlet
% FRET Efficiency
8
6
4
2
0
-2
ns
ns
control
bleached
control
bleached
septated
interphase
F
Input
IP
Flowthrough
Rax2-GFP + - + + - + + - +
mNG-Efc25 - + + - + + - + +
250kD
150kD
Rax2-GFP ~155kD
anti-GFP
150kD
100kD
mNG-Efc25 ~139kD
anti-mNG

Fig. S8. Rax1 and Rax2 form a complex but show no interaction with Efc25.
(A) Colocalization of mNG-Rax1 and Rax2-mScarlet. Arrows mark regions of colocalization. **(B)** Quantification of FRET efficiency between mNG-Rax1 and Rax2-mScarlet. Each point represents the mean of an independent experiment ($n \geq 118$, $N = 3$). Significance determined by a paired one-tailed t-test. **(C)** Colocalization of Efc25-mScarlet with mNG-Rax1 or Rax2-GFP. Arrows marks regions of colocalization. **(D,E)** Quantification of FRET efficiency between Efc25-mScarlet and mNG-Rax1 (F) or Rax2-GFP (G). Each point represents the mean of an independent experiment ($n \geq 21$, $N=3$). Significance determined by repeated measures one-way ANOVA followed by Šídák's multiple comparisons test. % FRET efficiency is the increase in donor fluorescence after photobleaching of the acceptor. Control measurements were taken from unbleached cells in the same field. Scale bars are 5 µm. Error bars are mean±s.d. * = $p < 0.05$, ns = not significant. **(F)** mNG-Efc25 does not co-immunoprecipitate with Rax2-GFP. Membrane fractions from cells expressing Rax2-GFP, mNG-Efc25, or both were immunoprecipitated with anti-GFP beads. Western blotting was then performed with anti-GFP and anti-mNG antibodies.

Journal of Cell Science • Supplementary information

A

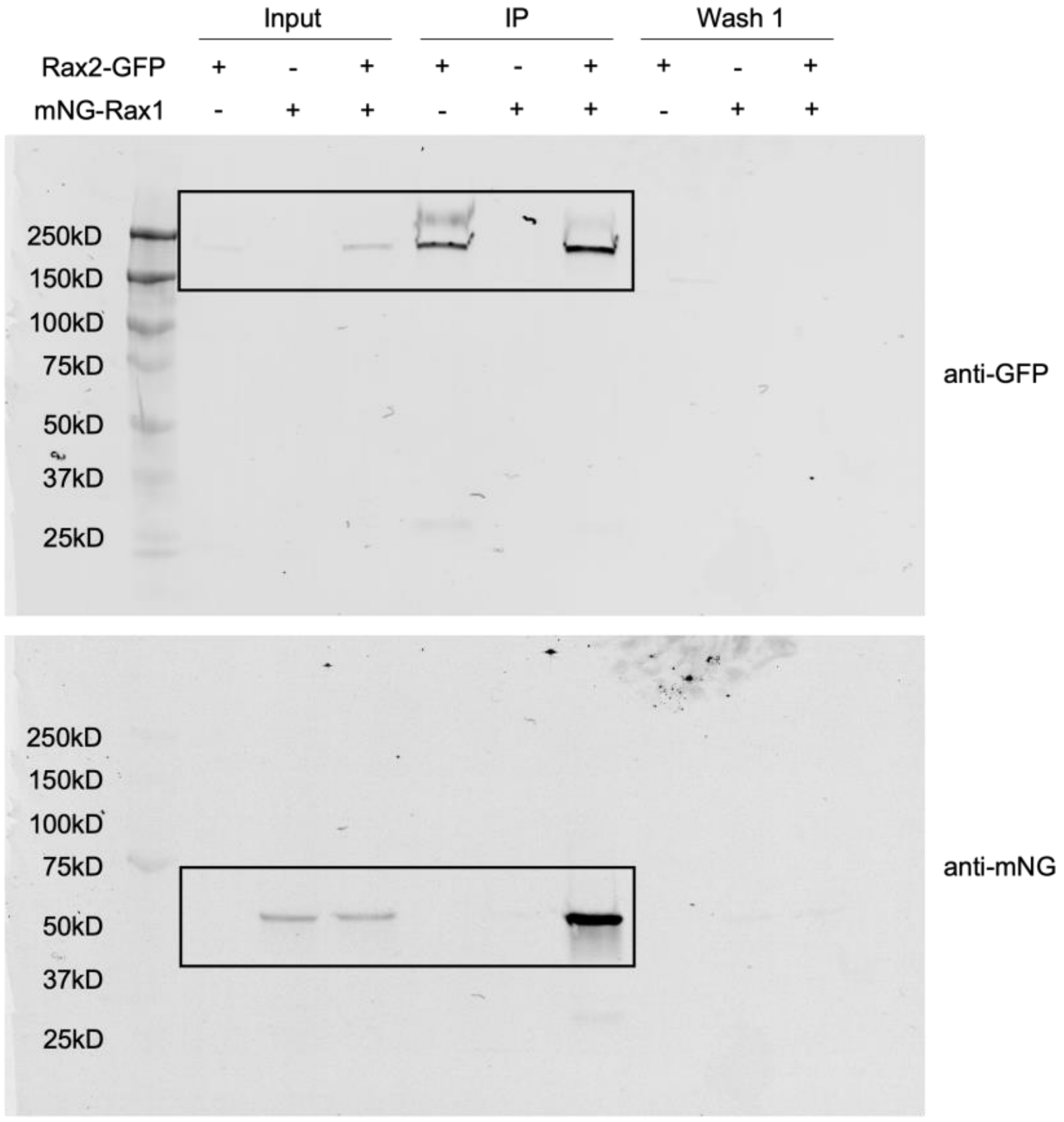

B

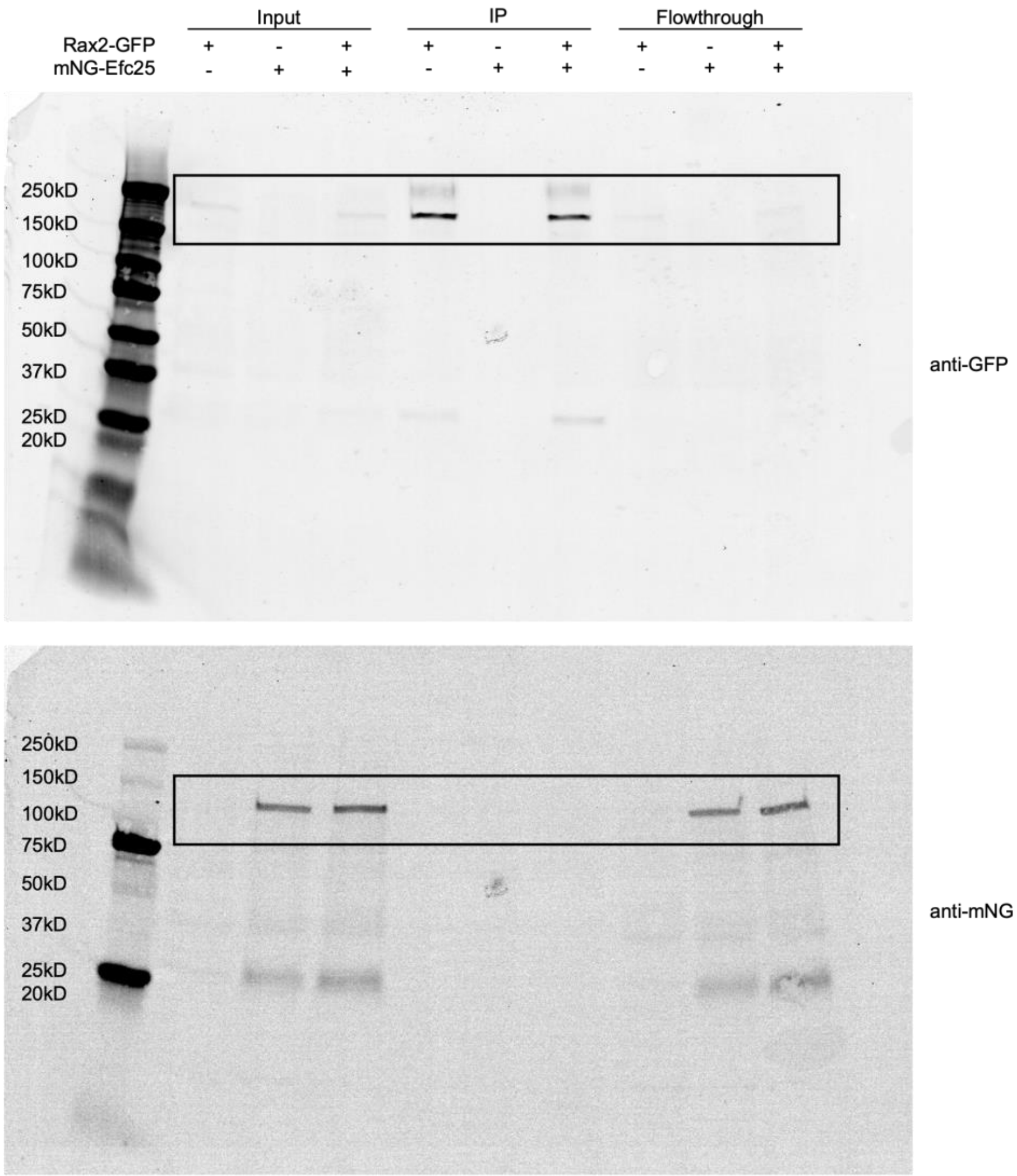

Journal of Cell Science • Supplementary information

Fig. S9. Blot transparency. Full western blots from Fig. 2B (A) and Fig. S8 (B). Boxes mark the areas shown in the figures. Ladder is Precision Plus Protein Dual Color Standards (BioRad). For each panel, both images are of the same blot with the top imaged in the 700nm channel and the bottom imaged in the 800nm channel.

Table S1. Percentage of bipolar cells in *rax1⁺* and *rax1*Δ strains.

	% Bipolarity	
Background	*rax1⁺*	*rax1*Δ
wt	71.9 ± 3.06	84.2 ± 2.88
*gef1*Δ	54.5 ± 5.61	79.0 ± 1.52
*scd2*Δ	22.7 ± 1.89	42.0 ± 1.68
*rga3*Δ	77.6 ± 0.910	84.5 ± 0.985
*rga4*Δ	73.5 ± 1.29	85.4 ± 3.53
*rdi1*Δ	57.8 ± 8.41	81.5 ± 6.97
*bud6*Δ	57.2 ± 6.40	67.3 ± 2.16

The mean and standard deviation of the percentage of bipolar cells as determined by calcofluor staining. Means calculated from 3 independent experiments (n ≥ 97 cells, N = 3). Values for the wildtype *rax1⁺* and *rax1*Δ strains are the mean and standard deviation of the plot in Fig. 1B.

Journal of Cell Science • Supplementary information

Table S2. List of strains used in this study.

Strain	Genotype	Origin	Used
PN567	*h+ ade6-704 leu1-32 ura4-D18*	P. Nurse	Figs 1A-G, 6A,B, S1, & Table S1
YMD1866	*h- rax1Δ::kanMX6 ade6-704 leu1-32 ura4-D18*	This study	Figs 1C-G, 6A,B, S1
YMD1882	*h+ rax1Δ::kanMX6 ade6-704 leu1-32 ura4-D18*	This study	Fig. 1A,B & Table S1
FV0001	*h+ orb2-34 ade6-M216 leu1-32*	F. Verde	Fig. 1H,I
YMD2346	*rax1Δ::kanMX6 orb2-34 ade6- leu1-32*	This study	Fig. 1H,I
YMD2351	*h- rax2-GFP:kanMX6 ura4-D18 ade6-704 leu1-32*	This study	Figs 2A-C,E,F, S2A,C, S3A,C,D, S9A
YMD2732	*h- mNeonGreen-gly8-rax1 fex1+ fex2+ his3+ ura4-D18 ade6- leu1-32*	This study	Figs 2A,B,D,G,H, S2A,D, S3A, S9A
YMD2717	*mNeonGreen-gly8-rax1 rax2-GFP:kanMX6 fex1+ fex2+ his3+ ura4-D18 ade6- leu1-32*	This study	Figs 2B, S9A
FV1335	*h+ CRIB-3xGFP:ura4+ ura4-D18 leu1-32*	F. Verde	Figs 3A,B,E-G, 6C,D, 7C,D, 8, S4A,C,F, S6
YMD1913	*CRIB-3xGFP:ura4+ rax1Δ::kanMX6 ura4-d18 leu1-32*	This study	Figs 3A,B,E-G, S4A,C,F, S6
YMD1176	*h+ scd1-mNG:kanMX6 ade6-704 leu1-32 ura4-D18*	Lab strain	Figs 3A,C, S4D,G, S5A-C
YMD1909	*h+ rax1Δ::kanMX6 scd1-mNG:kanMX6 ade6-704 leu1-32 ura4-D18*	This study	Figs 3A,C, S4D,G, S5A-C
FV1118	*h+ scd2-GFP:kanMX6 leu1-32 ura4-D18*	F. Verde	Figs 3A,D, S4B,E,H, S5D-F
YMD1911	*h- rax1Δ::kanMX6 scd2-GFP:kanMX6 leu1-32 ura4-D18*	This study	Figs 3A,D, S4B,E,H, S5D-F
YSM3098	*h- ura4-D18 leu1-32:RasAct-3GFP:leu1+*	S. Martin (Merlini et al., 2018)	Fig. 4A-G
YMD2397	*rax1Δ::kanMX6 leu1-32::RasAct-3xGFP:leu1+ ura4-D18*	This study	Fig. 4A-G
YMD2621	*tea1Δ::ura4+ leu1-32::RasAct-3xGFP:leu1+ ura4-D18*	This study	Fig. 4H

Journal of Cell Science • Supplementary information

YMD2679	*rax1Δ::kanMX6 efc25-gfp:kanMX6 ura4-D18*	This study	Figs 5A-C,G, S7A
YMD2680	*efc25-gfp:kanMX6 ura4-D18*	This study	Figs 5A-C,G, S7A
YMD2640	*h+ leu1-32::pjk148-Pnmt41-mNeonGreen-gly8-efc25:leu1+ efc25Δ::kanMX6 ade6-704 ura4-D18*	This study	Figs 5D-F,H,I, S7B, S8F, S9B
YMD2664	*h+ leu1-32::pjk148-Pnmt41-mNeonGreen-gly8-efc25:leu1+ efc25Δ::kanMX6 rax1Δ::kanMX6 ade6-704 ura4-D18*	This study	Figs 5D-F,H,I, S7B
YMD1547	*h+ leu1-32::pjk148-Pnmt41-leu1+*	Lab strain	Fig. 5I
YMD2856	*h- rax1Δ::kanMX6 leu1-32::pjk148-Pnmt41-leu1+*	This study	Fig. 5I
YMD2525	*h- efc25Δ::kanMX6 rax1Δ::kanMX6 ura4-D18 leu1+*	This study	Fig. 6A,B
YMD2546	*h+ efc25Δ::kanMX6 ade6-704 leu1-32 ura4-D18*	This study	Fig. 6A,B
YMD2613	*efc25Δ::kanMX6 CRIB-3xGFP:ura4+ ura4-*	This study	Figs 6C,D, 8A-C,G
YSM4482	*h90 gap1Δ::hphMX ura4+ ade6+ leu1+ his+*	S. Martin	Fig. 7A,B
YMD2467	*h+ ura4+ ade6-704 leu1-32*	This study	Fig. 7A,B
YMD2873	*rax1Δ::kanMX6 ura4+ ade6-704 leu1-32*	This study	Fig. 7A,B
YMD3131	*h90 rax1Δ::kanMX6 gap1Δ::hphMX ura4+ ade6+ leu1+ his+*	This study	Fig. 7A,B
YSM3421	*h- gap1Δ::hphMX CRIB-3xGFP:ura4+*	S. Martin (Lamas et al., 2020)	Figs 7C,D, 8D-G
YMD1268	*scd2Δ::kanMX6 CRIB-3xGFP:ura4+ ura4-*	Lab strain	Fig. 8A-C
YMD525	*h- rlc1-tdTomato:natMX6 sad1-mCherry:kanMX6*	Lab strain	Fig. S1C-E
YMD2573	*rax1Δ::kanMX6 rlc1-tdTomato:natMX6 sad1-mCherry:kanMX6*	This study	Fig. S1C-E
YMD2246	*h+ rax2-GFP:kanMX6 ura4-D18 ade6-704 leu1-32*	This study	Figs S2B, S8F, S9B
YMD2285	*h- rax1Δ::kanMX6 rax2-GFP:kanMX6 ade6-704 leu1-32 ura4-D18*	This study	Fig. S2B
YMD2410	*atb2-mCherry:natMX lifeact-GFP:leu1+*	Lab strain	Fig. S3A
YMD3086	*sty1Δ::natMX rax2-GFP:kanMX6 ura4-D18 leu1-32 ade6-*	This study	Fig. S3B-D
YMD2847	*mNeonGreen-gly8-rax1 rax2-gly8-mScarlet:natMX6 fex1+ fex2+ his3+ ura4-D18 ade6- leu1-32*	This study	Fig. S8A,B

Journal of Cell Science • Supplementary information

YMD2733	*mNeonGreen-gly8-rax1 efc25-mScarletI:natMX6 fex1+ fex2+ his3+ ura4-D18 ade6- leu1-32*	This study	Fig. S8C,D
YMD2739	*efc25-gly8-mScarletI:natMX6 rax2-gfp:kanMX6 ura4-D18 leu1-32 ade6-*	This study	Fig. S8C,E
YMD2665	*leu1-32::pjk148-Pnmt41-mNeonGreen-gly8-efc25:leu1+ efc25Δ::kanMX6 rax2-GFP:kanMX6 ade6-704 ura4-D18*	This study	Figs S8F, S9B
YMD776	*h- rdi1Δ::kanMX6 ura4-D18*	Lab strain	Table S1
YMD1894	*rax1Δ::kanMX6 rdi1Δ::kanMX6*	This study	Table S1
YMD476	*h- gef1Δ::ura4+ ura4-*	Lab strain	Table S1
YMD1887	*gef1Δ::ura4+ rax1Δ::kanMX6 ura4-*	This study	Table S1
YSM3131	*h- rga3Δ::kanMX6 ade6- leu1-32 ura4+*	S. Martin (Gallo Castro and Martin, 2018)	Table S1
YMD1929	*rax1Δ::kanMX6 rga3Δ::kanMX6 ade6- leu1-32 ura4+*	This study	Table S1
FV244	*h90 ura4-D18 scd2Δ::ura4+*	F. Verde	Table S1
YMD2443	*rax1Δ::kanMX6 scd2Δ::ura4+ ura4-D18 leu1- ade6-*	This study	Table S1
FV862	*h- bud6Δ::kanMX*	F. Verde	Table S1
YMD2619	*rax1Δ::kanMX6 bud6Δ::kanMX6*	This study	Table S1
YMD664	*h+ rga4Δ::ura4+ ura4-*	Lab strain	Table S1
YMD2868	*rax1Δ::kanMX6 rga4Δ::ura4+ ura4-*	This study	Table S1
YLM153	*h- efc25-gfp:kanMX6 ura4+ leu1+ ade6+*	S. Martin	Strain construction
FV2895	*h- efc25Δ::kanMX6 ura4+ leu1+*	F. Verde (Chen et al., 2019)	Strain construction
JB355	*h- fex1Δ fex2Δ his3-D1 ura4-D18 leu1-32 ade6-M216*	J. Berro (Fernandez and Berro, 2024)	Strain construction
FV467	*h+ tea1Δ::ura4+ ura4-D18 ade6-M210*	F. Verde	Strain construction
PN972	*h- ura4+ ade6+ leu1+*	Paul Nurse	Strain construction
JM1168	*h+ sty1Δ::natMX ura4-D18 leu1-32 ade6-M21X [pREP4X-sty1+:ura4+]*	J. Moseley (Schutt and Moseley, 2017)	Strain construction

Journal of Cell Science • Supplementary information

References

Chen, C., Rodriguez Pino, M., Haller, P. R. and Verde, F. (2019). Conserved NDR/LATS kinase controls RAS GTPase activity to regulate cell growth and chronological lifespan. *Mol Biol Cell* **30**, 2598–2616.

Fernandez, R. and Berro, J. (2024). CRISPR-Cas9 editing efficiency in fission yeast is not limited by homology search and is improved by combining gap-repair with fluoride selection. *microPublication Biology*.

Gallo Castro, D. and Martin, S. G. (2018). Differential GAP requirement for Cdc42-GTP polarization during proliferation and sexual reproduction. *J Cell Biol* **217**, 4215–4229.

Lamas, I., Merlini, L., Vještica, A., Vincenzetti, V. and Martin, S. G. (2020). Optogenetics reveals Cdc42 local activation by scaffold-mediated positive feedback and Ras GTPase. *PLoS Biol* **18**, e3000600.

Merlini, L., Khalili, B., Dudin, O., Michon, L., Vincenzetti, V. and Martin, S. G. (2018). Inhibition of Ras activity coordinates cell fusion with cell–cell contact during yeast mating. *J Cell Biol* **217**, 1467–1483.

Schutt, K. L. and Moseley, J. B. (2017). Transient activation of fission yeast AMPK is required for cell proliferation during osmotic stress. *Mol Biol Cell* **28**, 1804–1814.

Wickham, H. (2016). *ggplot2: Elegant Graphics for Data Analysis*. Springer-Verlag New York.
