# Supplementary material for: Memory of growth promotes early Ras GTPase activation to establish dominance of inherited cell ends in Schizosaccharomyces pombe
Source: J Cell Sci. 2026 Oct 12;139(19):jcs264600. doi: 10.1242/jcs.264600 (PMC13741952; doi:10.1242/jcs.264600)
Supplement: Supplementary file 2 [file joces-139-264600_review_history.pdf]

Memory of growth promotes early Ras GTPase activation to establish dominance of inherited cell ends in *Schizosaccharomyces pombe*

Walker H. Vickers and Maitreyi E. Das
DOI: 10.1242/jcs.264600

Editor: Michael Way

Review timeline
Original submission: 14 November 2025
Editorial decision: 20 December 2025
First revision received: 30 June 2026
Editorial decision: 10 August 2026
Second revision received: 26 August 2026
Accepted: 27 August 2026

Original submission

First decision letter

MS ID#: jcs.264600

MS TITLE: Memory of growth promotes early Ras GTPase activation to establish dominance of inherited cell ends in S. pombe

AUTHORS: Walker H Vickers; Maitreyi Das

ARTICLE TYPE: Research Article

Dear Dr Das,

We have now reached a decision on the above manuscript.

As you will see, the reviewers raise a number of substantial criticisms that prevent me from accepting the paper at this stage. Collectively, the feeling is the study is too preliminary and better evidence is required to support your conclusions. If you think that you can deal satisfactorily with the reviewers criticisms and comments on revision, I would be pleased to see a revised manuscript. We would then return it to the reviewers.

Reviewer 1

SUMMARY OF THE ADVANCE MADE IN THIS PAPER AND ITS POTENTIAL SIGNIFICANCE TO THE FIELD

The manuscript "Memory of growth promotes early Ras GTPase activation to establish dominance of inherited cell ends in S. pombe" examines how fission yeast cells initially grow from their old ends immediately after division, before growth initiates at the new ends formed by cytokinesis. Although several mutants defective in this growth pattern have been identified in fission yeast, the underlying mechanisms remain unclear. The authors follow up on prior reports by other groups on Rax1 and Rax2 to investigate their roles in conferring dominance to old end growth. They show that old end growth depends on Rax1-Rax2, which recruit the Ras GEF Efc25, and suggest that Ras1 activation enables the transition of Cdc42 activity from the division site to the old end. This manuscript addresses an important question of broad interest to cell biologists and presents several

© 2026. Published by The Company of Biologists under the terms of the Creative Commons Attribution License (https://creativecommons.org/licenses/by/4.0/).

intriguing observations. However, the mechanism remains suggestive rather than fully defined at the molecular level. Additional characterization of these observations and more precise descriptions would make the findings more compelling. The major and minor issues are summarized below.

SUGGESTIONS TO AUTHORS

Major comments [Please request additional experiments only if they are essential for supporting the conclusions; authors should be encouraged to highlight any claims that are preliminary or speculative, or to discuss any pitfalls or alternative interpretations in a 'Limitations' section]

1. The difference in Ras activity at cell ends and division sites in dividing cells between wild type and rax1Δ is clearly shown. However, the difference in Cdc42 activation between these seems less clear. Is the few-minute difference in CRIB-GFP localization at the ends in rax1Δ versus wild-type cells (Fig 3E) significant? The results shown in Fig. 3F & G seem to be based on 10 cells per strain. Given the high cell-to-cell variation within the same strain and the relatively small differences between the two strains, the sample size is too small. If overall growth or cell cycle progression is slower in rax1Δ cells than in wild type (although Fig 1E indicates there is little difference in total growth between rax1+ and rax1Δ), such differences might be observed even if Rax1 does not specifically affect the timing of the arrival of these polarity factors at the cell ends. Furthermore, it remains unclear how Ras activation at cell ends is linked to Cdc42 activation to promote old end growth.

2. There are some overstatements, and a more precise description would help clarify several confusing concepts. The expressions "history of growth" and "memory of growth" appear multiple times in the manuscript, including the abstract: "we demonstrate the importance of memory of growth in the establishment of polarity and maintaining persistent sites of growth across generations." However, it is unclear whether Rax1-Rax2 are long-lasting proteins that mark the growth site for multiple generations (as in their budding yeast homolog) or whether they are re-established at old ends every cell division cycle. None of the presented data demonstrates that the Rax1-Rax2 complex remains persistently at the growth site across generations. This claim would be more convincing if Rax1 or Rax2 localized to old ends throughout multiple division cycles and if these proteins indeed exhibited slow turnover lasting multiple generations.

Despite referring to "memory of growth", the authors compare only the role of the budding yeast Rax1-Rax2 complex in recruiting the bipolar landmark Bud8 (which is linked to the downstream GTPases Rsr1 and Cdc42). However, the function of Rax1-Rax2 as a 'cytokinesis remnant' in budding yeast is indeed more important as a form of cellular memory transmitted across multiple generations. Rax1-Rax2 (a cytokinesis remnant that persistently remains at old division sites) recruits the negative polarity cues Nba1 and Nis1, which in turn recruit Rga1, a Cdc42 GAP, via direct interactions. Rga1 plays a critical role in inactivating Cdc42 at these old division sites, in addition to clearing Cdc42-GTP at the division site soon after cytokinesis. While Rax1-Rax2 in fission yeast may function via a distinct mechanism, their results do not clearly demonstrate that Rax1 functions as a persistent site marker for cell end growth across generations.

3. Although Rax1 has been suggested to interact with Efc25, the results shown in Fig 7 are not very convincing. Because Tea1-GFP localizes to both cell ends and the division site, the data using GBP-Etc25 and Tea1-GFP (Fig 7A -B) are confusing and do not provide additional insight. While the Rax1-Rax2 interaction is convincing, with higher FRET efficiency and a statistically significant difference compared to the negative control, the Rax1-Efc25 interaction is not compelling. The co-localization data are very weak (Fig 7E), and the FRET efficiency (Fig 7F) is also low. Could the authors use an alternative method to test the physical interaction between Rax1 and Efc25?

Minor comments

1. Fig. 1D. It is not clear how NETO is determined from these bright-field images. Isn't a fixed reference point necessary to monitor new end growth, such as a fluorescent marker indicating the division site? If these were Calcofluor-stained cells, it would be easier to interpret the data, so including the corresponding fluorescence images would help.

2. On page 8, the subtitle (Rax1 promotes the timely transition of Cdc42 activity from the division site to the old end of separating cells) and related figures are confusing. Rax1 does not

© 2026. Published by The Company of Biologists under the terms of the Creative Commons Attribution License (https://creativecommons.org/licenses/by/4.0/).

affect Cdc42-GTP levels at the old end but instead affects Cdc42-GTP or polarity factors only at the new end (Fig. 3B). However, in Fig S2A, there is no significant difference in Cdc42-GTP levels at the new ends between rax1+ and rax1Δ cells, despite the clear differences in Scd1 and Scd2 levels at the new end between these strains. In addition, how do the data in Fig 3B differ from those in Fig S2, A-C? Are Cdc42-GTP levels at the new end significantly different between rax1+ and rax1Δ cells when presented as tip fraction, but not when directly compared as mean intensity? It might be helpful to compare these polarity factors at each end side-by-side: e.g., by comparing CRIB-3XGFP levels at the new and old ends in each strain to determine if there is indeed a more equal distribution between the two ends in rax1Δ cells. There seem still significant higher levels of these polarity factors at the old end in rax1D cells. It is also unclear which specific timepoints were chosen for these comparisons and how cells at the specific stages were identified for analyses.

3. Images are often unclear. In Fig. S3A, the localization of Scd1-mNG to the old end is not very clear. It seems that there are some signals before the arrowhead-marked time points.
If there is an increase in these polarity factors' localization to the new end in rax1Δ (as suggested in Figs 3 & S2), how do those time points compare to Fig S3B?
In Fig. 6A, Efc25-GFP signals at cell ends are very unclear. Although the authors suggest that "the signal in rax1+ cells Efc25-GFP intensity peaks at the tips of the cell ends, and that this peak is severely diminished in rax1Δ cells (Fig 6B)", the signals at cell ends are unclear, even in rax1+ cells, making it somewhat uncomfortable to rely solely on the normalized plots.

Reviewer 2

The manuscript "Memory of growth promotes early Ras GTPase activation to establish dominance of inherited cell ends in S. pombe" from Vickers and Das, proposes a role for Rax1/Rax2 proteins as landmarks that promote growth from the old cell ends after division in fission yeast. Rax2 had been previously shown to localize to cell poles and promote growth at the old end (Choi et al 2006). In the first three figures, the authors analyse the phenotype of rax1ï¤„ mutants, reporting a subtle polarity defect, and (re-)investigate the localization of Rax1 and Rax2. These data are consistent with previous studies. Indeed, it was previously shown that rax2ï¤„ cells display a mild defect in old end growth after division and that Rax2 localization to the cell tips depends on both actin and microtubule cytoskeletons (Choi et al., 2006). As detailed below, there are however important issues with the methodology and quantifications that need to be addressed.

In the second part of the paper, the authors show that Rax1/2 promote the accumulation of active Ras1 GTPase and its GEF activator Efc25 at the tips of dividing cells (the old end after division). Because Ras1 acts upstream of Cdc42, the authors propose that Rax1/2-mediated localization of Ras1-GTP facilitates the rapid transition of Cdc42 to the old end following cell division. While this is an interesting idea, it is not well supported by the experimental findings. In fact, both the epistasis data between *rax1Δ* and *efc25Δ* and the forced recruitment of Efc25 appear to contradict the proposed model.

In the final figure, the authors perform FRET experiments to test a potential interaction between Rax1/2 and Efc25, suggesting a possible direct interaction between Rax1 and Efc25. However, based on the data presented, no interaction between these proteins can be conclusively demonstrated.

Overall, the manuscript contains some preliminary and potentially interesting observations, but the phenotypes are weak and the experiments too preliminary to support a cohesive, publishable story.

Specific comments:

- Quantification of bipolarity in Figures 1, 5, 6 and 7.

The reported differences are very small, and the current quantification methods lack sufficient sensitivity, due to difficulty with the calcofluor staining. It is very difficult to distinguish bipolar from non-bipolar cells. Take for example Figure 5A, where asterisks indicate bipolar cell. In the bottom-left panel, the right-most cell without asterisk is not - to my eyes - significantly different from the one above, which is marked with an asterisk. Given the very small differences in % of monopolar vs bipolar cells throughout the manuscript, one needs a more reliable, quantitative method to assess the phenotype. Previous studies, including the previous study on Rax2, quantified

© 2026. Published by The Company of Biologists under the terms of the Creative Commons Attribution License (https://creativecommons.org/licenses/by/4.0/).

similar phenotypes using an assay where cells are first stained with FITC-lectin, followed by washout, regrowth and calcofluor staining (Arellano et al., 2002; Choi et al., 2006). This leaves FITC-clear regions at growth sites, which makes quantification more reliable.

- A second problem is that almost all measurements are done on non-synchronized cell cultures. Because the transition between monopolar and bipolar growth in fission yeast cells is controlled by the cell cycle, any variation in the length of one cell cycle phase would result in changes in the proportion of bipolar cells, independently of any effect on cell polarity. In fact, previous data had shown that rax2*Δ* have cell size and cell growth defects (Choi et al, 2006). Thus, it is critical to assess whether the observed differences are not due to cell cycle differences between WT and rax1*Δ* (or any other mutant investigated). At least some analyses should be repeated in synchronized cell populations.

- Quantifications of signals at growing/non-growing ends after division in Figure 1H, 3 and 5: There is a similar issue here. Old/new ends cannot be reliably defined from still images of unsynchronized cultures. These measurements should be performed using time-lapse movies, to accurately distinguish the two cell ends after division and to reliably identify the new. Given the small differences reported, cell synchronization and precise identification of the new end would significantly strengthen the analysis.

- FRAP experiments in Figure 2.
The differences in fluorescence recovery between treated and untreated cells appear convincing. However, it is not always clear whether the entire signal was bleached. Given the weakness of the signal, we recommend bleaching a larger region (the entire tip), to ensure that recovery does not result from lateral diffusion of residual, unbleached protein at the membrane. Furthermore, the authors interpret their data as Rax1/2 localizing in a growth-dependent manner, whereas previous work, which had shown that LatB and MBC treatments also cause Rax2 mislocalization from cell tips (Choi et al., 2006), interpreted localization dependent on the cytoskeleton. To distinguish between the two, the authors could perform similar experiments in sty1*Δ* cells treated with CK666 (or LatA), as these cells continue to grow even upon actin depolymerization.

- Model that Rax1-dependent maintenance of Ras1-GTP at cell poles during division promotes old end re-start:
As stated, the model is interesting but is not supported by the data provided. First, the epistasis analysis between rax1 and efc25 is not convincing, as efc25*Δ* and rax1ï¤„ efc25ï¤„ double mutants would be expected to phenocopy the rax1ï¤„ mutant. Second, the forced recruitment of Efc25 to cell poles (by Tea1) - though it seems to yield a functional Efc25 (cells are rod shaped indicating that GBP-Efc25 rescues the efc25*Δ*) - does not appear to rescue the rax1ï¤„ phenotype, and in fact causes a rax1*Δ*-like phenotype in the WT background. This is inconsistent with the proposed model. The authors could also investigate the effect of constitutively active Ras1 mutants on bipolar growth. In any case, in current state, this model is not supported.

- The FRET experiment does not convince of an interaction between Rax1 and Efc25. This sort of inconclusive data is not helpful at this stage. If an interaction exists, it will need to be established by other approaches.

In summary, the absence of Ras1-GTP and Efc25 at the poles of dividing cells in rax1*Δ* is clear, but the functional outcome of this delocalization remains to be established

---

First revision

<u>Author response to reviewers' comments</u>

We thank the reviewers for their comments and suggestions. We have incorporated the suggested changes and this has significantly enhanced the quality of this document. We have addressed the individual comments as described below.

© 2026. Published by The Company of Biologists under the terms of the Creative Commons Attribution License (https://creativecommons.org/licenses/by/4.0/).

Reviewer 1: SUMMARY OF THE ADVANCE MADE IN THIS PAPER AND ITS POTENTIAL SIGNIFICANCE TO THE FIELD

The manuscript "Memory of growth promotes early Ras GTPase activation to establish dominance of inherited cell ends in S. pombe" examines how fission yeast cells initially grow from their old ends immediately after division, before growth initiates at the new ends formed by cytokinesis. Although several mutants defective in this growth pattern have been identified in fission yeast, the underlying mechanisms remain unclear. The authors follow up on prior reports by other groups on Rax1 and Rax2 to investigate their roles in conferring dominance to old end growth. They show that old end growth depends on Rax1-Rax2, which recruit the Ras GEF Efc25, and suggest that Ras1 activation enables the transition of Cdc42 activity from the division site to the old end. This manuscript addresses an important question of broad interest to cell biologists and presents several intriguing observations. However, the mechanism remains suggestive rather than fully defined at the molecular level. Additional characterization of these observations and more precise descriptions would make the findings more compelling. The major and minor issues are summarized below.

SUGGESTIONS TO AUTHORS

Major comments [Please request additional experiments only if they are essential for supporting the conclusions; authors should be encouraged to highlight any claims that are preliminary or speculative, or to discuss any pitfalls or alternative interpretations in a 'Limitations' section]

1. The difference in Ras activity at cell ends and division sites in dividing cells between wild type and rax1Δ is clearly shown. However, the difference in Cdc42 activation between these seems less clear. Is the few-minute difference in CRIB-GFP localization at the ends in rax1Δ versus wild-type cells (Fig 3E) significant? The results shown in Fig. 3F & G seem to be based on 10 cells per strain. Given the high cell-to-cell variation within the same strain and the relatively small differences between the two strains, the sample size is too small. If overall growth or cell cycle progression is slower in rax1Δ cells than in wild type (although Fig 1E indicates there is little difference in total growth between rax1+ and rax1Δ), such differences might be observed even if Rax1 does not specifically affect the timing of the arrival of these polarity factors at the cell ends. Furthermore, it remains unclear how Ras activation at cell ends is linked to Cdc42 activation to promote old end growth.

We agree that a larger sample size would be beneficial due to the variation between cells. We imaged additional replicates, increasing the sample size to 20 cells per strain, and find a clear delay in old-end activation of Cdc42 in *rax1Δ* cells (Fig. 3E). The delay in old-end dominance remains similar to our previous results at ~9 minutes later in *rax1Δ* cells, this delay is statistically significant (Fig. 3G). We do not find any significant difference in the generation time of *rax1Δ* compared to *rax1$^+$* and do not observe any growth defects (Fig. S1), which indicates that the differences in the timing of Cdc42 activation in *rax1Δ* cells are not due to differences in overall growth or the cell cycle.
In fission yeast Ras1 was shown to physically and functionally interact with the Cdc42 GEF and its scaffold Scd1 and Scd2. Ras1 has been shown to interact with Scd1 in complex with its scaffold Scd2 as per two-hybrid assay in Chang et al., 1994.
Furthermore, Ras1 has been shown to promote the recruitment of Scd1 to the cell cortex to facilitate Cdc42 activity in Lamas et al., 2020. To further verify that Ras1 activation promotes Cdc42 activation, we performed timelapse imaging of CRIB-3xGFP in dividing *efc25Δ* cells and see a significant delay in Cdc42 activation at the old end (Fig. 6C,D). We also see early Cdc42 activation at the old end when the GAP of Ras1, Gap1, is lost (Fig. 7C,D). Thus, together with previously published reports our data indicate that Ras1 activation promotes Cdc42 activation at the cell ends.

2. There are some overstatements, and a more precise description would help clarify several confusing concepts. The expressions "history of growth" and "memory of growth" appear multiple times in the manuscript, including the abstract: "we demonstrate the importance of memory of growth in the establishment of polarity and maintaining persistent sites of growth

© 2026. Published by The Company of Biologists under the terms of the Creative Commons Attribution License (https://creativecommons.org/licenses/by/4.0/).

across generations." However, it is unclear whether Rax1-Rax2 are long-lasting proteins that mark the growth site for multiple generations (as in their budding yeast homolog) or whether they are re-established at old ends every cell division cycle. None of the presented data demonstrates that the Rax1-Rax2 complex remains persistently at the growth site across generations. This claim would be more convincing if Rax1 or Rax2 localized to old ends throughout multiple division cycles and if these proteins indeed exhibited slow turnover lasting multiple generations.
Despite referring to "memory of growth", the authors compare only the role of the budding yeast Rax1-Rax2 complex in recruiting the bipolar landmark Bud8 (which is linked to the downstream GTPases Rsr1 and Cdc42). However, the function of Rax1- Rax2 as a 'cytokinesis remnant' in budding yeast is indeed more important as a form of cellular memory transmitted across multiple generations. Rax1-Rax2 (a cytokinesis remnant that persistently remains at old division sites) recruits the negative polarity cues Nba1 and Nis1, which in turn recruit Rga1, a Cdc42 GAP, via direct interactions. Rga1 plays a critical role in inactivating Cdc42 at these old division sites, in addition to clearing Cdc42-GTP at the division site soon after cytokinesis. While Rax1-Rax2 in fission yeast may function via a distinct mechanism, their results do not clearly demonstrate that Rax1 functions as a persistent site marker for cell end growth across generations.

The reviewers raise an important point and highlight how Rax1/2 in fission yeast functions differently from that in budding yeast. While we did investigate whether Rax1/2 in fission yeast may have a similar role in negatively regulating Cdc42 as it does in budding yeast, our data does not support this hypothesis. We have looked at the localization of Cdc42 GAPs in the *rax1Δ* mutant and have not seen any difference in localization from *rax1+*. We also find no evidence that Rax1/2 accumulate in the birth scar which is analogous to the bud scar in budding yeast. Because of the shape and growth pattern of fission yeast, the raised birth scars which mark old division sites are present at the cell sides, where growth is already inhibited. Based on our results, Rax1/2 in fission yeast act as markers for where the site of polarity was located in the previous generation, rather than as markers for the previous division site as they do in budding yeast.
Our data show that Rax1/2 are delivered to the cell ends in a growth dependent manner. We find that Rax1/2 delivery to the ends only occurs in interphase and stops once the cell enters division (Fig. S2C,D). For Rax1/2 to act as a memory of growth, they need to be delivered in a growth dependent manner and persist at that end throughout division so as to promote reactivation of growth at that end when the cell exits division. Our data in Figs 2 and S2 support this model. To avoid confusion, we have replaced the phrase “across generations” with “from one generation to the next” in the abstract as this is a better description of how Rax1/2 function as a memory of growth.

3. Although Rax1 has been suggested to interact with Efc25, the results shown in Fig 7 are not very convincing. Because Tea1-GFP localizes to both cell ends and the division site, the data using GBP-Etc25 and Tea1-GFP (Fig 7A -B) are confusing and do not provide additional insight. While the Rax1-Rax2 interaction is convincing, with higher FRET efficiency and a statistically significant difference compared to the negative control, the Rax1-Efc25 interaction is not compelling. The co-localization data are very weak (Fig 7E), and the FRET efficiency (Fig 7F) is also low. Could the authors use an alternative method to test the physical interaction between Rax1 and Efc25?

We agree with reviewers that the data is not convincing. One of the challenges of this analysis is the fact that both Rax1 and Rax2 are transmembrane proteins with low abundance in the cell. Nevertheless, we have successfully immunoprecipitated Rax2- GFP from membrane fractions and do not see any interaction with Efc25. Thus, based on negative co-immunoprecipitation data, we cannot conclude that Rax1/2 are directly interacting with Efc25 (Fig. S8F). It is possible that Rax1/2 promote Efc25 recruitment via an indirect mechanism and this will be investigated further. As discussed by reviewers, the increased recruitment of GBP-mScarlet-Efc25 to the division site as well as the cell ends prevent us from distinguishing the effect of old-end localization of Efc25 from localization to the division site, and the reason for increased bipolarity in the *rax1*$^{+}$ Tea1-GFP cells was unclear. Because of these issues, the experiment was not able to address the question it was intended to answer, and so has been omitted.

Minor comments
1. Fig. 1D. It is not clear how NETO is determined from these bright-field images. Isn't a fixed

© 2026. Published by The Company of Biologists under the terms of the Creative Commons Attribution License (https://creativecommons.org/licenses/by/4.0/).

reference point necessary to monitor new end growth, such as a fluorescent marker indicating the division site? If these were Calcofluor-stained cells, it would be easier to interpret the data, so including the corresponding fluorescence images would help.

One of the advantages of the fission yeast model in cell morphology investigations is the clear appearance of the birth scar. We use this easily identifiable raised birth scar as the fixed reference point to measure new-end growth. This is the standard in the field (Mitchison & Nurse, 1985).

2. On page 8, the subtitle (Rax1 promotes the timely transition of Cdc42 activity from the division site to the old end of separating cells) and related figures are confusing. Rax1 does not affect Cdc42-GTP levels at the old end but instead affects Cdc42-GTP or polarity factors only at the new end (Fig. 3B). However, in Fig S2A, there is no significant difference in Cdc42-GTP levels at the new ends between rax1+ and rax1Δ cells, despite the clear differences in Scd1 and Scd2 levels at the new end between these strains. In addition, how do the data in Fig 3B differ from those in Fig S2, A-C? Are Cdc42-GTP levels at the new end significantly different between rax1+ and rax1Δ cells when presented as tip fraction, but not when directly compared as mean intensity?
It might be helpful to compare these polarity factors at each end side-by-side: e.g., by comparing CRIB-3XGFP levels at the new and old ends in each strain to determine if there is indeed a more equal distribution between the two ends in rax1Δ cells. There seem still significant higher levels of these polarity factors at the old end in rax1D cells. It is also unclear which specific timepoints were chosen for these comparisons and how cells at the specific stages were identified for analyses.

We agree that Rax1 does not affect Cdc42-GTP levels at the old end. In fact, our data show that Cdc42-GTP levels alone are not always directly correlated to the growth rate at that end. Our data indicates that it is the timing of Cdc42 reactivation at the old end that plays a critical role in old end dominance (Figs 3E-G, 6, 7). While Rax1 does not appear to affect the total level of Cdc42-GTP at the old end in interphase cells, the results in Fig 3E-G show that Cdc42 activation is delayed at the old end and remains longer at the division site in *rax1Δ* cells, which is what the subtitle is referring to. This delay appears to reduce old end dominance thereby leading to increased bipolarity in the *rax1*D cells.
Because CRIB-3xGFP is a fluorescent probe, we observe some variation in overall fluorescence between experiments. Thus, while CRIB-3xGFP is a useful tool to observe where Cdc42 is active and the dynamics of Cdc42 activation within a cell, it is not always the best measure of differences in levels of intensity between cells. For this reason, we measured the tip fraction to compare the distribution of Cdc42-GTP between the two ends of the cell. We further validated our findings by measuring the intensity of the endogenously tagged proteins Scd1-mNG and Scd2-GFP. This explains the difference between Fig. 3B (tip fraction) and the former Fig. S2A (now Fig. S4C, tip intensity).
It is correct that the old ends of *rax1Δ* cells do still have higher levels of polarity factors, however the difference between the old end and new end is smaller than in *rax1+* cells.
To aid in showing this change in distribution, we have included heat maps that plot the tip fraction of intensity versus cell length for both CRIB-3xGFP and Scd2-GFP (Fig. S4A,B). In wildtype cells we see that shorter cells have a more asymmetric distribution of active Cdc42 and become more symmetric as cells grow in length and bipolarity increases, as has been shown previously in Das et al., 2012. However, we see a decrease in asymmetric distribution of Cdc42 activity even at shorter cell lengths in *rax1Δ* cells corresponding to increased bipolarity in these cells.
For Fig. 3A-D and former Fig. S2 (now Fig. S4) asynchronous interphase cells were measured. One of the advantages of fission yeast is that dividing cells can be easily identified through fluorescent signal by the presence of two nuclei or a nucleus undergoing division. In addition, Cdc42-GTP and its regulators disperse from cell ends and localize to the division site during mitosis, providing an additional method to identify mitotic cells. Cells undergoing mitosis, septation, or cell separation were excluded from the measurements.

3. Images are often unclear. In Fig. S3A, the localization of Scd1-mNG to the old end is not very clear. It seems that there are some signals before the arrowhead-marked time points.
If there is an increase in these polarity factors' localization to the new end in rax1Δ (as suggested in Figs 3 & S2), how do those time points compare to Fig S3B?

© 2026. Published by The Company of Biologists under the terms of the Creative Commons Attribution License (https://creativecommons.org/licenses/by/4.0/).

We agree that Scd1-mNG signal in former Fig. S3A (now Fig. S5A) is somewhat unclear. Scd1-mNG is a low abundance protein that can be difficult to image, especially in timelapse due to photobleaching. This difficulty is why we also imaged Scd2-GFP, as it is a better readout for Scd1 levels in cells. This is because Scd2 is the scaffold for Scd1 and they colocalize in cells. Moreover, Scd1 is present in higher amounts in cells according to expression data on PomBase and as (Pascal et al., 2026, Harrell et al., 2024). In addition, because accumulation at the ends is gradual, visually identifying the specific time point where Scd1-mNG or any of Cdc42 and its regulators arrives at the cell end is difficult and prone to human error. To avoid this, we measured intensity over time to observe changes in old end localization with less subjectivity (Fig. S5B and E). For the purposes of marking timepoints in former Fig. S3A (now Fig. S5A) we chose to highlight the point where localization was clearly present across most of the cell tip. The timing in the current Fig. S5C, as well as Figs S5F and 3G, was determined by the point in each cell where the intensity at the old end began to be consistently higher than the new-end intensity.
The cells measured in Figs 3A-D and S2 (now S4) include all the interphase cells post cell separation. A large proportion of the cells measured for those figures would therefore be past the timepoints measured in Fig. S3 (now S5) which only monitor newly separated cells. Moreover, Figs 3A-D and S2 (now S4) show data from still images while Fig. S3 (now S5) show data from timelapse images and thus undergo photobleaching. Given the difference in the nature of imaging and the cell cycle stage of the cells imaged we cannot directly compare the fluorescent intensities at the cells from these different experiments. Regardless, in both experiments we find that the old end is less dominant in *rax1*D cells. The timelapse imaging highlights how the timing of Cdc42 activity at the old end is required for its dominance. The still images show that cells with diminished old-end dominance have higher Cdc42-GTP at the new end.

In Fig. 6A, Efc25-GFP signals at cell ends are very unclear. Although the authors suggest that "the signal in rax1+ cells Efc25-GFP intensity peaks at the tips of the cell ends, and that this peak is severely diminished in rax1Δ cells (Fig 6B)", the signals at cell ends are unclear, even in rax1+ cells, making it somewhat uncomfortable to rely solely on the normalized plots.

We agree with the reviewer that Efc25-GFP levels are unclear. This low abundance of Efc25-GFP led us to express mNG-Efc25 under the *nmt41* promoter, which showed clear localization to dividing cell ends and allowed us to validate what we observed in Efc25-GFP (Fig. 5D-F). As *rax1Δ* cells had an overall increase in both Efc25-GFP and mNG-Efc25 levels, normalization was performed to show the distribution at the cell ends while correcting for this increase in total fluorescence. For transparency, we have included plots showing the non-normalized data in Fig. S7.

*Reviewer 2: The manuscript "Memory of growth promotes early Ras GTPase activation to establish dominance of inherited cell ends in S. pombe" from Vickers and Das, proposes a role for Rax1/Rax2 proteins as landmarks that promote growth from the old cell ends after division in fission yeast. Rax2 had been previously shown to localize to cell poles and promote growth at the old end (Choi et al 2006). In the first three figures, the authors analyse the phenotype of rax1*D *mutants, reporting a subtle polarity defect, and (re-)investigate the localization of Rax1 and Rax2. These data are consistent with previous studies. Indeed, it was previously shown that rax2*D *cells display a mild defect in old end growth after division and that Rax2 localization to the cell tips depends on both actin and microtubule cytoskeletons (Choi et al., 2006). As detailed below, there are however important issues with the methodology and quantifications that need to be addressed.*

In the second part of the paper, the authors show that Rax1/2 promote the accumulation of active Ras1 GTPase and its GEF activator Efc25 at the tips of dividing cells (the old end after division). Because Ras1 acts upstream of Cdc42, the authors propose that Rax1/2-mediated localization of Ras1-GTP facilitates the rapid transition of Cdc42 to the old end following cell division. While this is an interesting idea, it is not well supported by the experimental findings. In fact, both the epistasis data between rax1Δ and efc25Δ and the forced recruitment of Efc25 appear to contradict the proposed model.

In the final figure, the authors perform FRET experiments to test a potential interaction

© 2026. Published by The Company of Biologists under the terms of the Creative Commons Attribution License (https://creativecommons.org/licenses/by/4.0/).

between Rax1/2 and Efc25, suggesting a possible direct interaction between Rax1 and Efc25. However, based on the data presented, no interaction between these proteins can be conclusively demonstrated.

Overall, the manuscript contains some preliminary and potentially interesting observations, but the phenotypes are weak and the experiments too preliminary to support a cohesive, publishable story.

Specific comments:

- Quantification of bipolarity in Figures 1, 5, 6 and 7.
The reported differences are very small, and the current quantification methods lack sufficient sensitivity, due to difficulty with the calcofluor staining. It is very difficult to distinguish bipolar from non-bipolar cells. Take for example Figure 5A, where asterisks indicate bipolar cell. In the bottom-left panel, the right-most cell without asterisk is not -to my eyes - significantly different from the one above, which is marked with an asterisk. Given the very small differences in % of monopolar vs bipolar cells throughout the manuscript, one needs a more reliable, quantitative method to assess the phenotype. Previous studies, including the previous study on Rax2, quantified similar phenotypes using an assay where cells are first stained with FITC-lectin, followed by washout, regrowth and calcofluor staining (Arellano et al., 2002; Choi et al., 2006). This leaves FITC-clear regions at growth sites, which makes quantification more reliable.

At the reviewer's suggestion, we have performed the proposed experiment using lectin-AlexaFluor 488 and still see a significant increase in bipolar cells in *rax1Δ*, as well as increased bipolarity in *efc25Δ* cells and *rax1Δefc25Δ* that is similar to that of *rax1Δ* (Fig. 6A,B).

- A second problem is that almost all measurements are done on non-synchronized cell cultures. Because the transition between monopolar and bipolar growth in fission yeast cells is controlled by the cell cycle, any variation in the length of one cell cycle phase would result in changes in the proportion of bipolar cells, independently of any effect on cell polarity. In fact, previous data had shown that rax2Δ have cell size and cell growth defects (Choi et al, 2006). Thus, it is critical to assess whether the observed differences are not due to cell cycle differences between WT and rax1Δ (or any other mutant investigated). At least some analyses should be repeated in synchronized cell populations.

We have observed no significant growth defect in our *rax1Δ* strain compared to *rax1+*. In addition, our measurement of the timing of NETO and the growth rate at each end was done over the full cell cycle, from the onset of cell separation to the next division, and we observed no significant difference in the length of the cell cycle between *rax1Δ* and *rax1+* cells (Fig. S1).

- Quantifications of signals at growing/non-growing ends after division in Figure 1H, 3 and 5: There is a similar issue here. Old/new ends cannot be reliably defined from still images of unsynchronized cultures. These measurements should be performed using time- lapse movies, to accurately distinguish the two cell ends after division and to reliably identify the new. Given the small differences reported, cell synchronization and precise identification of the new end would significantly strengthen the analysis.
In fission yeast, the new end is identified as the end closest to the birth scar except in the case of some monopolar mutants which have disrupted growth patterns. This method of distinguishing the old end and new end is standard in the field as seen in Mitchison & Nurse, 1985, and continues to be used to identify old ends vs. new ends in images where cell division was not directly observed (Tatebe et al., 2008; Abenza et al., 2015; Revilla-Guarinos et al., 2016; Davì et al., 2018). Moreover, for many of the proteins analyzed, timelapse imaging for the length of time required to observe the daughter cells from division to late into bipolar growth would lead to significant photobleaching that would impair the ability to measure fluorescence intensities accurately. We find that, based on our 8-hour timelapse imaging of *rax1Δ*, identifying the new end with reference to the birth scar is reliable. To further support our findings, we have included heat-maps in Fig. S4A,B plotting tip fraction of CRIB-3xGFP and Scd2-GFP versus cell length that show the distribution of Cdc42 activation without distinguishing between old and new ends. Our results show that *rax1Δ* cells have decreased asymmetric distribution of active Cdc42 and Scd2-GFP localization

© 2026. Published by The Company of Biologists under the terms of the Creative Commons Attribution License (https://creativecommons.org/licenses/by/4.0/).

even in shorter cells (Fig. S4A,B). We have also included heat-maps of CRIB-3xGFP tip fraction versus cell length for *efc25Δ* and *gap1Δ* in Fig. 8G, which show that the majority of *efc25Δ* cells have a symmetric distribution of Cdc42 activity, while *gap1Δ* cells have increased asymmetric distribution of Cdc42 activity even in longer cells.
While the *pak1-ts* mutant in Fig 1H is one of the mutants where the identification of the new end can be more difficult, the phenotype described was identified by the lack of a birth scar near the non-growing end. Because division always creates a birth scar at the new end, and the identified cells contained either a single birth scar or two scars next to each other in the middle of the cell, the non-growing end (as determined by calcofluor staining) without a birth scar could be positively identified as the old end.

- FRAP experiments in Figure 2.
The differences in fluorescence recovery between treated and untreated cells appear convincing. However, it is not always clear whether the entire signal was bleached.
Given the weakness of the signal, we recommend bleaching a larger region (the entire tip), to ensure that recovery does not result from lateral diffusion of residual, unbleached protein at the membrane. Furthermore, the authors interpret their data as Rax1/2 localizing in a growth-dependent manner, whereas previous work, which had shown that LatB and MBC treatments also cause Rax2 mislocalization from cell tips (Choi et al., 2006), interpreted localization dependent on the cytoskeleton. To distinguish between the two, the authors could perform similar experiments in sty1Δ cells treated with CK666 (or LatA), as these cells continue to grow even upon actin depolymerization.

The reviewers raise an important consideration in highlighting that the localization we interpret as growth-dependent could be dependent on the cytoskeleton. To address this, we examined Rax1/2 localization upon disruption of actin and microtubules. Our results differ from those found in Choi et al., 2006, as we do not observe mislocalization of Rax2-GFP or mNG-Rax1 after 30 minutes of treatment with either LatB or MBC (Fig. S3A). In addition, we performed the suggested experiment and repeated FRAP of Rax2-GFP in *sty1Δ* cells treated with CK666 while bleaching a larger region of the cell tip. We find that Rax2-GFP shows recovery after photobleaching in *sty1Δ* cells treated with CK666 that is significantly higher than *sty1*$^+$ cells under the same conditions (Fig. S3B-D). While recovery was decreased compared to the DMSO control, this is likely due to decreased growth rate in CK666 treated cells. Based on our results, we find that localization of Rax1/2 is growth-dependent and is not dependent on the cytoskeleton. This data showing recovery of Rax2-GFP after photobleaching of the entire cell tip combined with our previous data showing a lack of recovery in non-growing cell ends support that this recovery is not due to lateral diffusion of unbleached protein and is the result of growth-dependent delivery of new protein (Figs S2C,D, S3B-D).

- Model that Rax1-dependent maintenance of Ras1-GTP at cell poles during division promotes old end re-start:
As stated, the model is interesting but is not supported by the data provided. First, the epistasis analysis between rax1 and efc25 is not convincing, as efc25Δ and rax1D efc25D double mutants would be expected to phenocopy the rax1D mutant. Second, the forced recruitment of Efc25 to cell poles (by Tea1) - though it seems to yield a functional Efc25 (cells are rod shaped indicating that GBP-Efc25 rescues the efc25Δ) - does not appear to rescue the rax1D phenotype, and in fact causes a rax1Δ-like phenotype in the WT background. This is inconsistent with the proposed model. The authors could also investigate the effect of constitutively active Ras1 mutants on bipolar growth. In any case, in current state, this model is not supported.

We agree with the reviewers that the results of the GBP-Efc25 Tea1-GFP experiment are difficult to interpret. Due to the increased recruitment of GBP-mScarlet-Efc25 to the division site as well as the cell ends, it was not possible to distinguish the effect of old- end localization of Efc25 from localization to the division site, and the reason for increased bipolarity in the *rax1*$^+$ Tea1-GFP cells was unclear. Because of these issues, the experiment was not able to address the question it was intended to answer, and so has been omitted.
At the reviewer's suggestion we performed lectin staining and washout followed by calcofluor staining and found that *efc25Δ* and *efc25Δrax1Δ* do phenocopy *rax1Δ* cells (Fig. 6A,B). Furthermore, *efc25Δ* cells show a significant delay in CRIB-3xGFP localization to the old ends of separating daughter cells even more than that observed in *rax1Δ* (Fig. 6C,D). This supports our

© 2026. Published by The Company of Biologists under the terms of the Creative Commons Attribution License (https://creativecommons.org/licenses/by/4.0/).

model that decreasing Ras1 activity delays Cdc42 activation at the old end thus diminishing its dominance.
As suggested, we also investigated the effect of increased Ras1 activity using the deletion of its only identified GAP, Gap1, as its deletion is known to cause Ras1 to be highly active across the entire cell cortex (Lamas et al., 2020). We find that *gap1Δ* cells have decreased bipolarity compared to the wildtype, and that the double deletion *gap1Δrax1Δ* has a similar decrease in bipolarity as *gap1Δ* alone (Fig. 7A,B). We also find that *gap1Δ* cells activate Cdc42 earlier at the old ends of dividing cells, and the Cdc42 activity is more asymmetrically distributed (Figs 7C,D, 8C-E). This supports our model, as increased Ras1 activity promotes early Cdc42 activation, leading to a more dominant old end. Our data with *efc25*D and *gap1*D together indicate that proper Ras1 activation at the old end is important for old end dominance.

- The FRET experiment does not convince of an interaction between Rax1 and Efc25.
This sort of inconclusive data is not helpful at this stage. If an interaction exists, it will need to be established by other approaches.

We agree with reviewers that the data is not convincing. One of the challenges of this analysis is the fact that both Rax1 and Rax2 are transmembrane proteins with low abundance in the cell. Nevertheless, we have successfully immunoprecipitated Rax2- GFP from membrane fractions and do not see any interaction with Efc25. Thus, based on negative co-immunoprecipitation data, we cannot conclude that Rax1/2 are directly interacting with Efc25 (Fig. S8F). It is possible that Rax1/2 promote Efc25 recruitment via an indirect mechanism and this will be investigated further.

In summary, the absence of Ras1-GTP and Efc25 at the poles of dividing cells in rax1Δ is clear, but the functional outcome of this delocalization remains to be established

Our data shows that Rax1 is required for proper recruitment of Efc25 and subsequent Ras1 activation at the old end of cells as they complete division. This leads to timely Cdc42 activation at the old ends and establishes old end dominance. With new data as suggested by the reviewers, using *efc25*D and *gap1*D mutants, we show that the functional outcome of altering Ras1 activity is corresponding changes in old end dominance.

Second decision letter

MS ID#: jcs.264600R1

MS TITLE: Memory of growth promotes early Ras GTPase activation to establish dominance of inherited cell ends in S. pombe

AUTHORS: Walker H Vickers; Maitreyi Das

ARTICLE TYPE: Research Article

Dear Dr Das,

We have now reached a decision on the above manuscript. It took longer than usual as we had to wait for the reviewer to return from holiday before they could submit their report. Rather than ask a new reviewer, which would have changed the goal posts, I felt I had no option but to wait, especially as the other reviewer declined to look at your paper again.

As you will see, the reviewer is still not convinced your study is worthy of publication in JCS and still raises some points. Given the situation, I think the easiest solution (assuming you would rather not do anymore expts) would be for you to tone things down/rephrase in places and add a limitations section at the end of the discussion. I hope that you will be able to carry these out because I would like to be able to accept your paper.

© 2026. Published by The Company of Biologists under the terms of the Creative Commons Attribution License (https://creativecommons.org/licenses/by/4.0/).

Reviewer 2

The authors have made some effort to make the story more convincing, by repeating some experiments and adding some data, and also removed data that was present in the first submission. However, it remains a rather tenuous story, reporting on quite minor phenotypes. The only really convincing piece of data is the lack of Ras1 activity at the poles of dividing cells in the rax1/2 mutants. The question of how Rax1/2 promote Ras1 activity at poles of dividing cells is completely open, as the authors could not demonstrate interaction with the Ras1 GEF Efc25 and in fact now report negative results. While I would encourage reporting of weak phenotypes, with appropriate phrasing to avoid overstatements, I am not convinced this story is strong enough for publication in JCS.

Specific comments:

- Quantification of bipolarity in Figures 1, 5, 6 and 7:
The authors have improved this quantification by adding lectin staining. I disagree with their interpretation that it is not necessary to examine possible cell cycle differences to firmly conclude on a direct effect on NETO. It would not be very difficult to use a cdc10-ts background to block cells in G1 and check whether rax1/2Δ cells are able to go through NETO. Given that previous published data on these mutants reported cell size and growth defects, it seems a little light to just state that they find otherwise and leave it at that.

- Quantifications of signals at growing/non-growing ends after division in Figure 1H, 3 and 5:
I would remain more cautious. Timelapse is possible with weak signals - one just needs to acquire only the DIC to visualize growth before acquiring a single fluorescence image.

- FRAP experiments in Figure 2:
I remain unconvinced whether Rax1/2 really localize in response to growth rather than in response to the cytoskeleton. The sty1Δ experiment in Fig S3 is not convincing. One cannot see any Rax2-GFP signal at the bleached cell tip in the timelapse provided. Looking at the control, DMSO-treated cell, the cortical signal is equivalent at both cell poles at the end of the experiment, and even from about 70 min. By contrast, there is clear cortical signal in the CK666-treated bottom cell pole, but nothing clear at the bleached poles even after 112 minutes. As the cell is growing (a bit), the authors must have moved the area to quantify, which brings unavoidable imprecision in measurements. I would interpret the increase they measure as background (perhaps vacuolar) signal. It does not look different from the sty1+ cells (in Fig2). Thus, an alternative interpretation is that the change of dynamics is a direct result of CK666 treatment rather than lack of growth. Because distinct interpretations can be made, there cannot be any firm conclusion that Rax1/2 act as memory of growth markers.
An added issue is the confusion between using LatB, which depolymerizes all actin structures, and CK-666, which will only block endocytosis.
Perhaps the issue can be resolved by careful rephrasing. The key point is whether Rax1/2 are localizing during the growth process (whether by the cytoskeleton or other) and whether their localization is stable enough to perdure as growth stops.

- Model that Rax1-dependent maintenance of Ras1-GTP at cell poles during division promotes old end re-start:
The addition of the gap1Δ partial monopolar phenotype and repeat of experiments with lectin staining helps. It remains the case that the now removed data of Efc25 recruitment to Tea1 seemed to cause a rax1/2Δ-like phenotype, which is not in agreement with the model.

- FRET experiment:
The authors now acknowledge that there is no evidence of interaction between Rax1/2 and Efc25. The figure S8 should be removed altogether, and negative data just cited in the text.

Second revision

© 2026. Published by The Company of Biologists under the terms of the Creative Commons Attribution License (https://creativecommons.org/licenses/by/4.0/).

Author response to reviewers' comments

We thank the reviewer for their comments. We have clarified the issues raised in their comments and have discussed the limitations of our data in the discussion. The specific responses are provided below.

Comments from the Reviewers:

Reviewer 2: The authors have made some effort to make the story more convincing, by repeating some experiments and adding some data, and also removed data that was present in the first submission. However, it remains a rather tenuous story, reporting on quite minor phenotypes. The only really convincing piece of data is the lack of Ras1 activity at the poles of dividing cells in the rax1/2 mutants. The question of how Rax1/2 promote Ras1 activity at poles of dividing cells is completely open, as the authors could not demonstrate interaction with the Ras1 GEF Efc25 and in fact now report negative results. While I would encourage reporting of weak phenotypes, with appropriate phrasing to avoid overstatements, I am not convinced this story is strong enough for publication in JCS.

Specific comments:

- Quantification of bipolarity in Figures 1, 5, 6 and 7:
The authors have improved this quantification by adding lectin staining. I disagree with their interpretation that it is not necessary to examine possible cell cycle differences to firmly conclude on a direct effect on NETO. It would not be very difficult to use a cdc10-ts background to block cells in G1 and check whether rax1/2Δ cells are able to go through NETO. Given that previous published data on these mutants reported cell size and growth defects, it seems a little light to just state that they find otherwise and leave it at that.

While measuring new-end growth of *rax1Δ* cells using a *cdc10-ts* background would be a simple experiment, it would not address whether potential cell-cycle differences play a role in premature NETO of *rax1Δ* cells, only whether loss of Rax1/2 allow NETO to bypass the cell-cycle requirement. We believe the proposed experiment would not provide additional information that our measurements of growth at each end over the full cell cycle in Figure 1C-G does not already provide. Our data show no change in generation time between *rax1Δ* and *rax1*$^{+}$ cells (Fig. S1B) and we have also imaged *rax1Δ* cells with the spindle pole bodies and cytokinetic ring labeled and see no difference in the timing of mitosis or cell separation. Since our measurements in Figure 1C-G begin at cell separation and under standard laboratory conditions fission yeast cells are already in G2 phase by the time cell separation is complete (Knutsen et al., 2011), our timelapse imaging should address any potential concerns for differences in the cell cycle. We have also provided an explanation of this in the discussion, page 16, lines 430-435. “The transition from monopolar to bipolar growth is also cell-cycle dependent. Cells arrested in G1 are mostly monopolar. Previous reports have shown that *rax2*D *cdc10-129* cells arrested in G1 show only a minor increase in bipolarity indicating that rax2D does not regulate polarity due to a cell cycle defect (Choi et al., 2006). Indeed, our data with *rax1*D does not show a cell cycle or growth defect.”

To address the reviewer’s point on the difference between previous findings of growth defects in *rax2Δ* cells and our results we have expanded our discussion of these differences in the relevant section. However, it is difficult to directly compare our results and the results in Choi et al., 2006, as our data is in a *rax1Δ* strain while the previous data was from *rax2Δ* cells. It is possible that Rax2 has an additional effect on cell growth distinct from Rax1. Additionally, the lack of statistical information on the data on cell size and growth in Choi et al., 2006 further make direct comparison challenging. We have discussed this on page 17 lines 435-439. “It should be noted that previous work in *rax2Δ* cells showed decreased growth (Choi et al., 2006). Rax2 contains a much larger extracellular region than Rax1 that appears to be heavily glycosylated (Johnson, 2019), so it is possible this difference reflects a *rax2*-specific role in cell growth, though additional research would be required to confirm this difference and identify the mechanism.”

- Quantifications of signals at growing/non-growing ends after division in Figure 1H, 3 and 5:
I would remain more cautious. Timelapse is possible with weak signals - one just needs to acquire

© 2026. Published by The Company of Biologists under the terms of the Creative Commons Attribution License (https://creativecommons.org/licenses/by/4.0/).

only the DIC to visualize growth before acquiring a single fluorescence image.

A discussion of this limitation brought up by the reviewer has been added to the relevant section.

- FRAP experiments in Figure 2:
I remain unconvinced whether Rax1/2 really localize in response to growth rather than in response to the cytoskeleton. The sty1Δ experiment in Fig S3 is not convincing. One cannot see any Rax2-GFP signal at the bleached cell tip in the timelapse provided.
Looking at the control, DMSO-treated cell, the cortical signal is equivalent at both cell poles at the end of the experiment, and even from about 70 min. By contrast, there is clear cortical signal in the CK666-treated bottom cell pole, but nothing clear at the bleached poles even after 112 minutes. As the cell is growing (a bit), the authors must have moved the area to quantify, which brings unavoidable imprecision in measurements. I would interpret the increase they measure as background (perhaps vacuolar) signal. It does not look different from the sty1+ cells (in Fig2). Thus, an alternative interpretation is that the change of dynamics is a direct result of CK666 treatment rather than lack of growth. Because distinct interpretations can be made, there cannot be any firm conclusion that Rax1/2 act as memory of growth markers.
An added issue is the confusion between using LatB, which depolymerizes all actin structures, and CK-666, which will only block endocytosis.
Perhaps the issue can be resolved by careful rephrasing. The key point is whether Rax1/2 are localizing during the growth process (whether by the cytoskeleton or other) and whether their localization is stable enough to perdure as growth stops.

Discussion of the limitations of the experiment discussed by the reviewer has been added to the relevant section and the conclusions drawn from our results have been rephrased to be more qualified and focus on the localization of Rax1/2 during growth and their stability at the ends. Additionally, the montage of Rax2-GFP localization to the end of a *sty1Δ* CK666-treated cell has been exchanged for a representative image that more clearly shows fluorescence recovery at the cell cortex distinct from background signal. While measurement of intensity at the growing cell end did require adjustment of the ROI, fluorescence recovery was measured using a segmented line across the tip of the cell and its position adjusted to align with the cell cortex and so would be minimally affected by increased cytoplasmic or vacuolar signal. Examples of ROI placement have been added to Figure S3 to clarify the methods used to measure recovery. The continued localization of Rax2-GFP to the non-bleached growing end of *sty1Δ* CK666- treated cells noted by the reviewer also supports our interpretation of these results, as the lack of any dispersal of Rax2-GFP from growing ends when Arp2/3 is inhibited suggests that its localization is maintained independently of branched actin.

- Model that Rax1-dependent maintenance of Ras1-GTP at cell poles during division promotes old end re-start:
The addition of the gap1Δ partial monopolar phenotype and repeat of experiments with lectin staining helps. It remains the case that the now removed data of Efc25 recruitment to Tea1 seemed to cause a rax1/2Δ-like phenotype, which is not in agreement with the model.

The goal of the removed experiment of Efc25 recruitment to Tea1 was to determine whether recruitment of Efc25 to the old ends could bypass Rax1/2 to promote old-end dominance. As most proteins that localize to the old ends also localize to the division site, this was a difficult question to investigate. While we hoped that recruitment by Tea1 would allow us to answer this question, the sequestering of GBP-mScarlet-Efc25 to both the old ends and the division site by Tea1-GFP prevented us from being able to address the goal of the experiment. We see increased bipolarity when Efc25 is sequestered to both old and new ends independent of *rax1*. As the reviewers did not seem to like the experiment, and the experiment was unable to effectively test our model, we decided to remove it from the manuscript. While the effect of stably sequestering GBP-mScarlet-Efc25 to Tea1-GFP is worth further investigation, we believe it is beyond the scope of this paper.

- FRET experiment:
The authors now acknowledge that there is no evidence of interaction between Rax1/2 and Efc25. The figure S8 should be removed altogether, and negative data just cited in the text.

We believe that negative data are important to include for the benefit of future research. In

© 2026. Published by The Company of Biologists under the terms of the Creative Commons Attribution License (https://creativecommons.org/licenses/by/4.0/).

addition, JCS guidelines discourage discussing data that is not shown.

Third decision letter

MS ID#: jcs.264600R2

MS Title: Memory of growth promotes early Ras GTPase activation to establish dominance of inherited cell ends in S. pombe

Authors: Walker H Vickers; Maitreyi Das

Article Type: Research Article

Dear Dr Das,

I am happy to tell you that your manuscript has been accepted for publication in Journal of Cell Science, pending standard publication integrity checks.

© 2026. Published by The Company of Biologists under the terms of the Creative Commons Attribution License (https://creativecommons.org/licenses/by/4.0/).
